# Advances in the Regulation of Lipid Metabolism by Non-Coding RNAs

**DOI:** 10.3390/ani15172621

**Published:** 2025-09-07

**Authors:** Yongdong Peng, Anqi Liu, Qifei Zhu, Xiaotong Liu, Bingbing Cai, Zhiyu Yan, Jiwei Gao, Ruchang Zhu, Changfa Wang

**Affiliations:** Liaocheng Research Institute of Donkey High-Efficiency Breeding and Ecological Feeding, College of Agriculture and Biology, Liaocheng University, Liaocheng 522000, China; pengyongdong@lcu.edu.com (Y.P.); 13668648783@163.com (A.L.); 18253004081@163.com (Q.Z.); lxt0188@163.com (X.L.); 13792483706@163.com (B.C.); 13589286792@163.com (Z.Y.); 17564298171@163.com (J.G.); zhuruchang200610@163.com (R.Z.)

**Keywords:** miRNAs, lncRNAs, circRNAs, lipid metabolism, therapeutic

## Abstract

Lipid metabolism is a fundamental biological process that ensures energy homeostasis and cellular integrity. This is an essential and complex biochemical process that is involved in energy storage and the maintaining of normal biological functions. Disorders of lipid metabolism are an urgent health problem, bringing about metabolic syndromes such as obesity, hepatic steatosis, adipose tissue dysfunction, diabetes, cardiovascular diseases, and certain cancers. An increasing body of research has emphasized the pivotal function of ncRNAs, particularly miRNAs, lncRNAs, and circRNAs, in controlling lipid metabolism and associated diseases. To identify novel therapeutic targets, it is necessary to understand the molecular mechanisms by which ncRNAs are involved in the pathobiology of lipid-associated diseases. This review summarizes the research findings on the role of ncRNA in lipid metabolism and the underlying mechanisms, emphasizing their potential as therapeutic targets for lipid-related diseases.

## Simple Summary

Lipid metabolism constitutes a critical and intricate biochemical system essential for energy storage and sustaining normal physiological functions. Disruption to this system has been associated with various diseases, including obesity, hepatic steatosis, diabetes, cardiovascular diseases and certain cancers. Numerous studies have investigated the regulatory role of non-coding RNAs (ncRNAs), particularly microRNAs (miRNAs), long non-coding RNAs (lncRNAs), and circular RNAs (circRNAs), in various biological processes linked to lipid metabolism and associated diseases. Understanding the molecular mechanisms by which ncRNAs are involved in the pathobiology of lipid-associated diseases will be required to identify novel therapeutic targets.

## 1. Introduction

Non-coding RNAs are a category of RNA molecules that do not translate into proteins. They regulate gene expression at the transcriptional, post-transcriptional, and translational levels. Given their regulatory functions, ncRNAs can be categorized as either housekeeping or regulatory [1]. Housekeeping ncRNAs are expressed in abundance and throughout the cell, and primarily regulate generic cellular functions. They include ribosomal RNAs (rRNAs), small nuclear RNAs (snRNAs), small nucleolar RNAs (snoRNAs), transfer RNAs (tRNAs), and telomerase RNAs [2,3]. Regulatory ncRNAs are key regulatory RNA molecules that regulate gene expression at various levels, including the epigenetic, transcriptional, and post-transcriptional levels. They mainly include miRNAs, lncRNAs, circRNAs, small interfering RNAs (siRNAs), and Piwi-interacting RNAs (piRNAs) [4,5,6].

**Figure 1:**
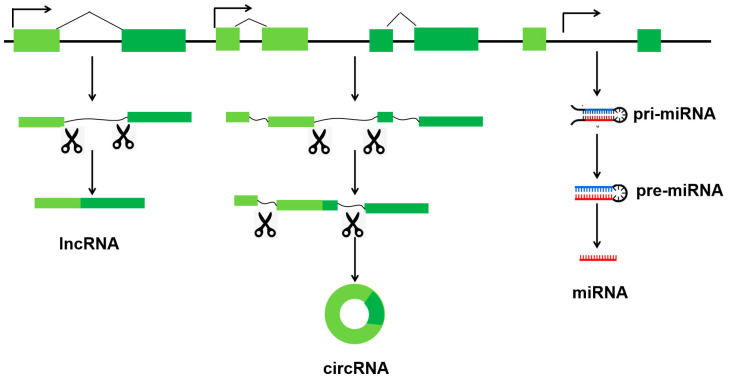
Distinct types of three ncRNAs (miRNAs, lncRNAs, and circRNAs) are transcribed from genomes.

Non-coding RNAs are categorized into two main groups according to their length of transcript. Short non-coding RNAs consist of fewer than 40 nucleotides (nt), while long non-coding RNAs (lncRNAs) consist of more than 200 nt. Short non-coding RNAs comprising the following: miRNAs (18–25 nt), piRNAs (21–36 nt) [7], siRNAs (19–23 nt) [8], and transfer RNA fragments (tRFs, 17–26 nt) [9]. As shown in Figure 1, three ncRNAs (miRNAs, lncRNAs, and circRNAs) are transcribed from genomes.

MiRNAs are a class of small, non-coding RNA molecules that play a key role in gene regulation. They are approximately 22 nt in length. They regulate the expression and stability of mRNAs post-transcriptionally via the RNA-induced silencing complex (RISC) [10]. LncRNAs are transcripts that are >200 nt in length and not translated into proteins. This heterogeneous group includes intergenic transcripts, enhancer RNAs, and sense or antisense transcripts that overlap other genes [11]. The regulatory mechanism of lncRNA is complex and highly organized. In the nucleus, they are involved in epigenetic and transcriptional regulation [12]. In the cytoplasm, they act as competing endogenous RNAs (ceRNAs), absorbing miRNAs and competing with protein-coding genes [13,14]. CircRNAs are a class of non-coding RNAs that have a covalently closed circular structure and lack 5′-caps or 3′-poly(A) tails. They can act as miRNA sponges or decoys to regulate target mRNAs [15]. Similarly, certain circRNAs can bind to particular proteins, effectively acting as ‘sponges’ for them [16]. Additionally, circRNAs can function as protein scaffolds, facilitating the assembly of large RNA–protein complexes, including ribosomal and spliceosomal RNAs [17]. Some circRNAs can also encode functional proteins [18,19].

Lipids not only function as structural components of cell membranes and play a critical role in energy metabolism, but also act as signaling molecules that influence membrane fluidity and mediate post-translational modifications [20,21]. Lipid metabolism is an essential and complex biochemical process that is crucial for energy storage and maintaining normal biological functions. With the help of related enzymes, lipids are digested and absorbed, synthesized, and broken down in living organisms, and then processed into the substances needed by the body to ensure normal physiological functioning. Disorders of lipid metabolism have become an urgent health concern, leading to various metabolic syndromes such as obesity, hepatic steatosis, adipose tissue dysfunction, diabetes, cardiovascular diseases, and certain cancers [15,22,23]. Therefore, it would be beneficial to explore the underlying regulatory mechanisms of lipid metabolism and identify critical regulatory molecules for developing treatments for the disease. Over the past decade, ncRNAs have emerged as genetic factors associated with lipid metabolism and related diseases [24,25,26]. This review primarily focuses on the role of ncRNAs, specifically miRNAs, lncRNAs, and circRNAs, in lipid metabolism and their importance as molecular biomarkers for the diagnosis and treatment of lipid-related diseases.

## 2. Role of ncRNAs in Adipogenesis

**Figure 2:**
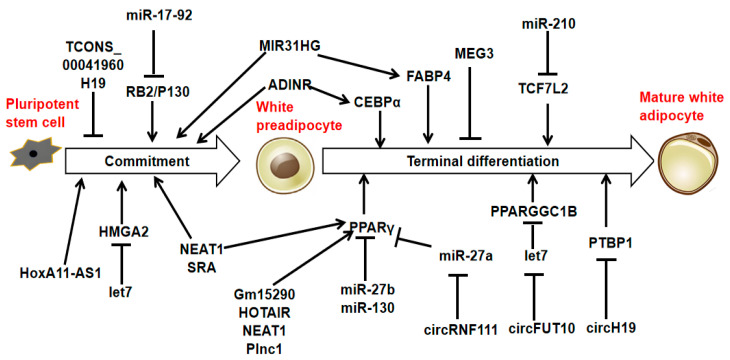
MiRNAs, lncRNAs, and circRNAs are associated with adipogenesis.

Adipocytes originate from multipotent mesenchymal stromal cells (MSCs), which can develop into different cell types, including adipocytes, chondrocytes, myocytes, and osteocytes [27]. In adipogenesis, MSCs are instructed to adopt an adipogenic fate by specific signaling molecules, which then leads to their transformation into preadipocytes. The preadipocytes then undergo mitotic clonal expansion and terminal differentiation, forming mature adipocytes. This process is regulated by key transcription factors [28,29]. Specific ncRNAs such as miRNAs, lncRNAs, and circRNAs promote or inhibit the differentiation process during adipogenesis, depending on the gene targets they regulate (Figure 2, Supplemental Tables S1–S3).

## 3. Role of ncRNAs in Lipid Metabolism

**Figure 3:**
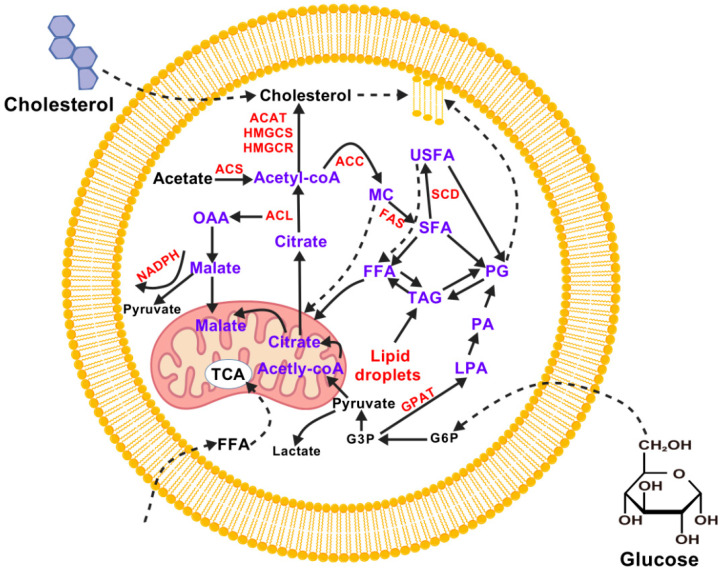
A summary of lipid metabolism in mammalian cells. ACAT (Acyl-CoA cholesteryl acyltransferase), ACC (Acetyl CoA Carboxylase), ACL (ATP citrate lyase), ACS (Acetyl CoA synthase), FAS (Fatty acid synthase), FFA (free FA), G3P (glyceraldehyde 3-phosphate), G6P (Glucose-6-Phosphate), GPAT (Glycerol-3-phosphate acyltransferase), HMGCS (Hydroxymethylglutaryl Coenzyme A Synthase), HMGCR (HMG-CoA reductase), LPA (lisophosphatidic acid), MC (malonyl-CoA), NADPH (Nicotinamide Adenine Dinucleotide Phosphate), OAA (oxaloacetate), PA (phosphatidic acid), PG (phophoglycerates), SCD (Stearoyl-CoA Desaturase), SFA (saturated fatty acid), TAG (triacyl glycerols), TCA (tricarboxylic acid), USFA (unsaturated fatty acid).

Lipids are hydrophobic molecules that are chemically diverse and play a variety of biological roles. These include fatty acids, triacylglycerols, phospholipids, sphingolipids, eicosanoids, cholesterol, bile salts, steroid hormones, and fat-soluble vitamins. They are a major source of energy and a key component of cell membranes. They also play a significant role in digestion and absorption, and participate in numerous signaling and regulatory processes [30] (Figure 3). Cholesterol can be obtained from animal fats in the diet or synthesized de novo from acetyl-CoA. It is an essential component of lipid bilayer membranes and a precursor of bile acids, salts, steroid hormones, and vitamin D, most of which are synthesized in the liver. They are released into the intestine, where they function as detergents to solubilize dietary fats. Steroid hormones are primarily synthesized in the adrenal glands and gonads. These hormones regulate energy metabolism and stress responses (glucocorticoids), salt balance (mineralocorticoids), and sexual development and function (androgens and estrogens) [31]. However, long-term high cholesterol levels have been linked to the progression of atherosclerosis, thereby raising the risk of heart attacks and strokes.

### 3.1. Role of miRNAs in Lipid Metabolism

**Figure 4:**
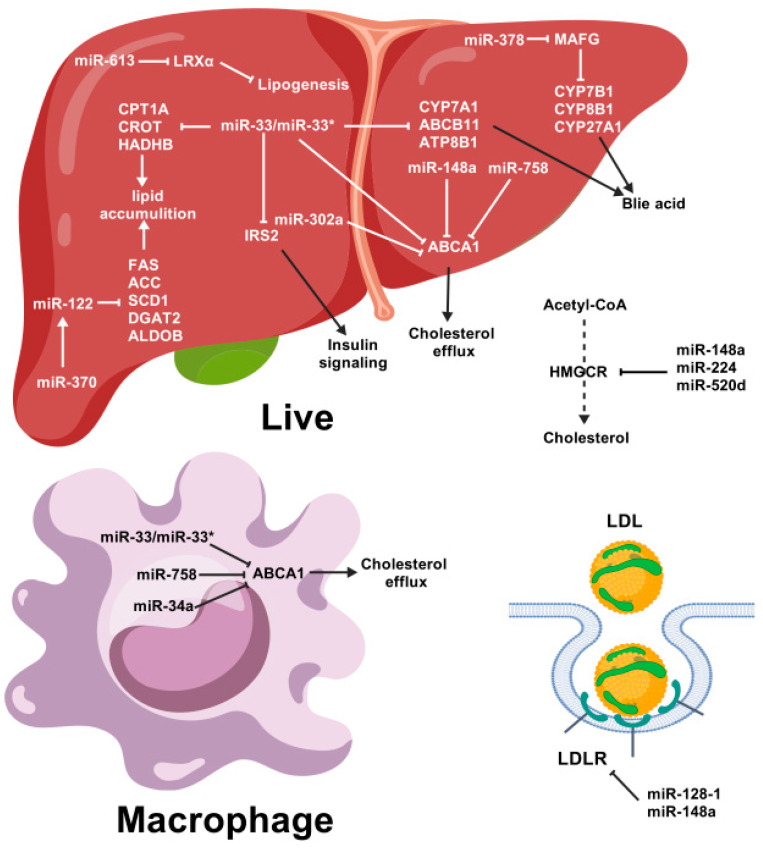
Summary of the possible roles of miRNAs in lipid metabolism. Pointed tip arrows indicate activation of the pathway, and blunted tip arrows indicate blocking of the pathway. miR-33* (passenger strand of miR-33), ABCA1 (ATP-binding cassette transporter A1), CPT1A (carnitine palmitoyltransferase 1A), CROT (carnitine O-octanoyltransferase), HADHB (Hydroxyacyl-CoA Dehydrogenase Trifunctional Multienzyme Complex Subunit Beta), CYP7A1 (cholesterol 7α-hydroxylase), HMGCR (HMG-CoA reductase), LDL (low-density lipoprotein), LDLR (low-density lipoprotein receptor), LPL (high-density lipoprotein).

Several studies have demonstrated that miRNAs play crucial roles in lipid metabolism (Figure 4). The first study to investigate the involvement of miRNA in regulating lipid metabolism was conducted in Drosophila. The deletion of miR-14 in Drosophila led to higher levels of triacylglycerol and diacylglycerol. Conversely, increasing the copy number of miR-14 had the opposite effect [32].

It has been reported that miR-7 stimulates the production of sterol response element-binding proteins 1 and 2 (SREBP1 and SREBP2), thereby upregulating genes involved in sterol biosynthesis. In turn, the activation of miR-7 is regulated by the peroxisome proliferator-activated receptor α (*PPARα*), which controls fatty acid metabolism. Therefore, miR-7 plays a key role in linking the metabolic pathways of sterols and fatty acids in the liver [33]. In both in vitro and in vivo settings, miR-7 controls cholesterol biosynthesis by modulating the activity of 24-dehydrocholesterol reductase (*DHCR24*) at the post-transcriptional level [34].

Compared with control cells, steatotic L02 cells exhibit increased expression of miR-10b. MiR-10b regulates cellular steatosis levels by targeting peroxisome proliferator-activated receptor-alpha (*PPARα*) expression, as *PPARα* has also been found to participate in steatosis [35]. The synthetic inhibition of miR-10b could also suppress β-adrenergic-induced lipolysis in adipocytes [36]. Additionally, the ABO locus exerts a trans-regulatory effect on several blood proteins, coronary artery disease, and total cholesterol (TC), which is mediated by miR-10b-5p [37].

The expression of miR-21 is lower in the livers of mice fed a high-fat diet than in those fed a standard diet. Overexpression of miR-21 significantly reduces the accumulation of intracellular lipids caused by stearic acid (SA). This is achieved by preventing the expression of *FABP7* [38]. The expression of a group of lipid metabolic genes differs in mice lacking the miR-21 gene compared to wild-type mice. Furthermore, miR-21 directly targets both *PPARα* and *IGFBP3* [39,40]. MiR-21 also targets programmed cell death 4 (*PDCD4*), thereby controlling the accumulation of lipids via the Toll-like receptor 4 (TLR4) and NF-κB pathways in macrophages stimulated by lipopolysaccharide [41]. The deletion of miR-21/miR-21* in hepatocytes increases insulin sensitivity and prevents fatty acid uptake, lipogenesis, gluconeogenesis, and glucose output [42]. Additionally, the expression of LDL receptor-related protein 6 (*LRP6*) is inhibited in HepG2 cells by miR-21, thereby inducing lipid production [43]. Overexpression of miR-21-5p reduces lipid content and lipid peroxidation in H9C2 cells. The abundance of miR-21-5p within the cell can modulate reliance on glycolytic and fatty acid oxidation pathways. The miR-21 antagomir increases the expression level of tissue inhibitor of metalloproteinases 3 (*TIMP3*), thereby improving insulin resistance and disordered lipid metabolism in STZ-induced T2DM rats [44]. Furthermore, miR-21-5p induces hepatic lipogenesis in chickens by targeting the nuclear factor I B (*NFIB*) and Kruppel-like factor 3 (*KLF3*) proteins, thereby suppressing the PI3K/AKT signaling pathway [45]. Furthermore, it has been found that miR-21-5p promotes differentiation and reduces the accumulation of lipid droplets induced by oleic acid in C2C12 myoblasts by targeting F-box protein 11 (*FBXO11*) [46].

MiR-30c is part of the relevant and complex miR-30 family. This family plays a variety of roles in physiological and pathological processes of living organisms, including the regulation of lipoprotein metabolism. It targets the microsomal triglyceride transfer protein (*MTP*), which facilitates the lipidation of newly synthesized apolipoprotein B (APOB) in the liver and is necessary for the production of very low-density lipoprotein (VLDL) and low-density lipoprotein (LDL). In mice, overexpressing miR-30c reduces the production and secretion of APOB-containing lipoproteins. This leads to lower plasma LDL levels. Furthermore, miR-30c inhibits hepatic lipid synthesis by targeting lysophosphatidylglycerol acyl-transferase 1 (*LPGAT1*) [47]. MiR-30c reduces microsomal triglyceride transfer protein expression and lipoprotein production, thereby lowering plasma cholesterol and mitigating atherosclerosis. It also prevents steatosis by reducing lipid synthesis [48]. In mice lacking the leptin receptor (db/db), miR-30c-5p improves hepatic steatosis by reducing the activity of *FAS*, a key enzyme in fatty acid biosynthesis. [49]. Circulating miR-30c shows a significant positive correlation with total and LDL cholesterol, suggesting a regulatory role in lipid homeostasis. It is transported in both exosomes and high-density lipoprotein 3 (HDL3), and its expression is significantly increased by pravastatin therapy, adding to the pleiotropic dimensions of statins [50]. Another study has shown that miR-30c decreases the activity of hepatic microsomal triglyceride transfer protein, as well as lipid synthesis and plasma cholesterol, in mouse models of both homozygous familial hypercholesterolaemia and type 2 diabetes [51]. Elevated levels of miR-122 and reduced levels of miR-30c in a high postprandial response (HPR) could be critical factors in the development of elevated or prolonged postprandial lipaemia. The miR-122/30c ratio was strongly correlated with microsomal triglyceride transfer protein (MTTP), apolipoprotein B-48 (apo B-48), and triglyceride (TG) levels, as well as chylomicron (CM) particle size [52].

MiR-33a and miR-33b (miR-33a/b) are intronic, meaning their encoding regions are embedded in the *SREBP2* and *SREBP1* genes, respectively. The overexpression of miR-33 downregulates the expression of the *ABCA1* and *ABCG1*, thereby decreasing plasma levels of HDL-C. Antisense oligonucleotides (ASOs) can be used to antagonize miR-33, which is sufficient to increase the expression of hepatic *ABCA1* and *ABCG1*, thereby raising circulating HDL-C levels [53,54]. These findings were further confirmed in mice and non-human primates lacking the miR-33 gene, which were treated with microRNA inhibitors [55,56,57,58,59]. Both miR-33a and miR-33b target genes involved in the β-oxidation of fatty acids. These genes include carnitine palmitoyltransferase 1A (*CPT1A*), carnitine O-octanoyltransferase (*CROT*), hydroxyacyl-CoA dehydrogenase/3-ketoacyl-CoA thiolase/enoyl-CoA hydratase (*HADHB*), 5′ adenosine monophosphate-activated protein kinase (AMPK), and sirtuin 6 (*SIRT6*). Furthermore, they target insulin receptor substrate 2 (IRS2), which is a vital part of the insulin signaling pathway in the liver [60]. Additionally, miR-33 directly targets cholesterol 7α-hydroxylase (*CYP7A1*), which mediates the transformation of cholesterol into bile salts. It also targets the bile salt exporter *ABCB11* and the aminophospholipid transporter *ATP8B1*, thereby controlling the final excretion of cholesterol from the body via fecal bile acids [61,62]. Experiments conducted using macrophages derived from mice deficient in miR-33 revealed that this microRNA is crucial for regulating cholesterol efflux in both in vivo and in vitro settings [63]. Additionally, miR-33 plays a crucial role in regulating lipid metabolism in chicken livers. It achieves this by negatively affecting the expression of two genes (*CROT* and *HADHB*), which encode essential enzymes for lipid oxidation [64]. Furthermore, the inhibition of cholesterol efflux in vascular endothelial cells by miR-33-5p is mediated through the targeting of citrate synthase and *ABCA1* [65].

The presence of elevated levels of miR-34a in obese individuals reduces the levels of NAD+ and sirtuin 1 (SIRT1) deacetylase activity by targeting *NAMPT* [66]. MiR-34a negatively regulates retinoid X receptor alpha (*RXRα*) by binding to its 3′-untranslated region (3′-UTR) and decreasing the induction of cytochrome P450 family 26 (*CYP26*) and cytochrome P450 family 3 subfamily A polypeptide 4 (*CYP3A4*) [67]. The use of lentivirus to downregulate miR-34a in obese mice was shown to reduce blood lipid levels, as well as increase the number of mitochondria and enhance their oxidative function in adipose tissue. It also significantly increased the expression of the beige fat marker proteins CD137 and UCP-1. Downregulation of miR-34a increases the expression of the FGF21 receptor complex (*FGFR1-βKL*) and *SIRT1*. This contributes to activation of the browning transcriptional program via FGF21/SIRT1-dependent deacetylation of PGC-1α [68]. Additionally, miR-34a reduces the expression of hepatocyte nuclear factor 4 alpha (*HNF4A*), a key transcription factor involved in lipid metabolism. This occurs in response to metabolic stress caused by the accumulation of excess fat in the liver [69]. The expression of miR-34a in visceral fat increases progressively as dietary obesity develops. However, selectively ablating miR-34a in adipose tissue mitigates the metabolic dysfunction induced by obesity and protects obese individuals from the exacerbated meta-inflammation and insulin resistance caused by dietary stress [70]. Like miR-33, miR-34a suppresses the expression of *ABCA1*/*ABCG1* in macrophages, thereby controlling cholesterol efflux and reverse cholesterol transport (RCT). Furthermore, miR-34a regulates macrophage polarization, thereby reducing inflammation and atherosclerosis. It does this by repressing the liver X receptor (*LXR*) and the Krüppel-like factor 4 (*KLF4*). A global loss of miR-34a reduces the absorption of cholesterol or fat in the intestine by inhibiting cytochrome P450 enzymes, such as *CYP7A1* and *CYP8B1* [71]. Furthermore, in cases of non-alcoholic fatty liver disease (NAFLD) accompanied by iron overload, miR-34a is responsible for regulating lipid metabolism by targeting *SIRT1* [72]. Additionally, miR-34a directly targets acyl-CoA synthetase long-chain family member 4 (*ACSL4*), thereby regulating the deposition of intramuscular fat in porcine adipocytes [73]. Pterostilbene has been shown to improve hepatic lipid accumulation in fructose-fed rats via the miR-34a/SIRT1/SREBP-1 pathway [74]. MiR-34a plays a pivotal role in the development and progression of NAFLD, inducing lipid absorption, lipogenesis, inflammation, and apoptosis. It also inhibits fatty acid oxidation [75]. Neochlorogenic acid (5-caffeoylquinic acid, or 5-CQA) alleviates lipid accumulation by downregulating miR-34a and activating the sirtuin 1 (SIRT1)/AMP-activated protein kinase (AMPK) pathway [76]. In addition, miR-34a encourages the deposition of fat in adipocytes and myoblasts by targeting *LEF1* via the Wnt signaling pathway [77]. Artemether (ATM) is a promising therapeutic agent for metabolic dysfunction-associated fatty liver disease (MAFLD), reducing lipid deposition by suppressing miR-34a-5p and upregulating PPARα [78]. Artemisia capillaris Thunb. Water extract (ACTE) stimulates SIRT1 by suppressing miR-34a-5p. This reduces liver lipid accumulation and improves diet-induced metabolic dysfunction-associated steatotic liver disease (MASLD) [79].

MiR-106b decreases the expression of *ABCA1* and impairs cellular cholesterol efflux in neurons. Furthermore, it was found that levels of secreted Aβ increased dramatically in Neuro2a cells transfected with miR-106b [80].

MiR-122 is an abundantly expressed microRNA that is found in the liver [81,82,83]. Inhibiting miR-122 in normal mice lowers their plasma cholesterol levels and accelerates the rate at which their livers oxidize and synthesize fatty acids and cholesterol. In a mouse model of diet-induced obesity, inhibiting miR-122 was found to reduce plasma cholesterol levels and significantly improve hepatic steatosis. This is accompanied by reductions in several lipogenic genes, including *FAS*, *ACC2*, and *SCD1* [84]. MiR-122a and miR-422a target *CYP7A1*, thereby inhibiting its expression. This process suppresses bile acid synthesis [85]. MiR-122 also increases the expression of lipogenic genes by initially activating *SREBP-1c* and *DGAT2*, which subsequently activate *FAS* and *ACC1* [86]. In addition, miR-122 plays a vital role in lipid accumulation (particularly triglycerides) in the liver by reducing Yin Yang 1 (*YY1*) mRNA to upregulate FXR-SHP signaling [87]. It is also crucial in goose fatty liver disease, targeting genes involved in lipid metabolism, such as aldolase B (*ALDOB*) and pyruvate kinase M2 (*PKM2*) [88]. Inhibiting miR-122 can protect hepatocytes against lipid metabolic disorders, such as NAFLD, by suppressing lipogenesis and increasing *SIRT1* levels. This activates the AMPK pathway [89]. Inhibiting MiR-122 reduces the accumulation of lipids and inflammation caused by oleic acid (OA) in L02 cells, potentially through the inhibition of the TLR4/MyD88/NF-κBp65 pathway [90].

The expression levels of miR-128-1 are high in adipose tissue, muscle, and the liver. It plays a key role in regulating the homeostasis of circulating lipoproteins [91,92]. There is a strong link between miR-128-1 and abnormal changes in total cholesterol (TC) and low-density lipoprotein cholesterol (LDL-C) levels. It targets *LDLR* and *ABCA1* directly, thereby controlling circulating lipoprotein metabolism. In human hepatoma cells and mouse macrophages, MiR-128-1 interacts with *ABCA1* to regulate cellular cholesterol efflux to ApoA1 [91]. Furthermore, inhibiting miR-128-1 using antisense technology in Apoe^-/-^ mice decreases circulating triglycerides (TGs) and hepatic steatosis, while improving insulin sensitivity and glucose tolerance [91,93]. Additionally, it has been suggested that miR-128-1 regulates genes involved in lipogenesis, including *FAS*, *SIRT1*, the NAD+-dependent energy sensor, and lysine deacetylase [91].

MiR-148a inhibits the expression of *LDLR* and *ABCA1*. This suppresses cellular cholesterol uptake and efflux, respectively [91,94]. Several laboratories independently found that SNPs in the promoter region of miR-148a (rs4722551, rs4719841, and rs6951827) were associated with altered blood levels of TC, LDL-C, and TAGs [91,95,96,97]. MiR-148a deletion promotes hepatic lipid accumulation and increases serum and hepatic total cholesterol (TC) levels in mice [98]. Additionally, miR-148a targets the pro-renin receptor (*PRR*), reducing its expression in human hepatic cells. This decreases LDL receptor (LDLR) protein abundance and affects LDL metabolism by suppressing *PRR* expression [99].

MiR-223 inhibits the expression of the scavenger receptor *SR-BI*, thereby repressing the uptake of HDL-C in human hepatocytes [100]. Another study shows that miR-223 also directly targets *SR-BI*, repressing its expression and regulating HDL-C uptake. Meanwhile, it suppresses cholesterol biosynthesis by directly inhibiting the enzymes responsible for sterol synthesis: 3-hydroxy-3-methylglutaryl-CoA synthase 1 (*HMGCS1*) and methylsterol monooxygenase 1 (*MSMO1*). At the same time, miR-223 indirectly increases the expression of *ABCA1* via *Sp3*. This enhances cellular cholesterol efflux [101]. Furthermore, the process of lipid deposition and inflammation is suppressed by miR-223, which functions by hindering the signaling of Toll-like receptor 4 in macrophages [102]. Additionally, miR-223 targets the gene for diacylglycerol lipase alpha (*DAGLA*), which is involved in lipid metabolism, and regulates its expression in chicken hepatocytes [103]. Extracellular vesicles derived from adipose-derived mesenchymal stem cells (ADSC-EVs) containing miR-223-3p can reduce lipid accumulation and liver fibrosis by repressing *E2F1*, thereby alleviating NAFLD [104].

MiR-224-5p regulates fatty acid metabolism by targeting *ACSL4* during the final stages of adipocyte differentiation [105]. Additionally, miR-224 influences the apoptosis of mammary epithelial cells and the production of triglycerides by downregulating the *ACADM* and *ALDH2* genes [106]. Both PCSK9 and IDOL are chaperone proteins that restrict the expression of LDLR at the cell surface. Meanwhile, HMGCR is the rate-limiting enzyme in cholesterol synthesis. MiR-224 and miR-520d target these three proteins and reduce their expression. This results in increased LDLR cell surface expression and LDL binding [107]. Bta-miR-224 targeting fatty acid-binding protein 4 (*FABP4*) may encourage biological processes such as the synthesis of triglycerides (TGs) and the formation of lipid droplets via *PPARγ* [108].

The development of nonalcoholic fatty liver in LDL receptor knockout mice fed a Western-type diet (WTD) is associated with a fivefold decrease in miR-302a expression. Decreased levels of microRNA-302a were found to increase the expression of its target genes, *ABCA1* and *ELOVL6*, in hepatocytes [109]. In primary macrophages, miR-302a targets *ABCA1* to regulate cellular cholesterol efflux [110].

MiR-370 directly targets *CPT1A*, thereby reducing the rate of fatty acid β-oxidation. Interestingly, transfecting the human hepatic cell line HepG2 with miR-370 upregulates the expression of miR-122, which leads to increased expression of lipogenic genes, including *SREBP1c* and *DGAT2*. This suggests that miR-370 provides an additional point of regulation in this pathway [86].

MiR-378/378*, which is located within the genomic sequence of peroxisome proliferator-activated receptor gamma coactivator-1 alpha (*PGC1α*), plays a key role in regulating lipid metabolism. During adipogenesis, the overexpression of miR-378/378* increases triacylglycerol accumulation through enhanced de novo lipogenesis. Conversely, knocking down miR-378 and/or miR-378* decreases this process. It has been shown that miR-378/378* specifically increases the transcriptional activity of *C/EBPα* and C/EBPβ [111]. Mice with a genetic deficiency in both miR-378 and miR-378* are resistant to obesity induced by a high-fat diet. They also exhibit enhanced mitochondrial fatty acid metabolism and increased oxidative capacity in insulin-targeted tissues. MiR-378 and miR-378* suppress *CRAT* (a mitochondrial enzyme involved in fatty acid metabolism) and *MED13* (a component of the Mediator complex that controls nuclear hormone receptor activity), respectively. The levels of these increase in the livers of mice lacking miR-378/378* [112]. In addition, miR-378 inhibits insulin signaling by targeting *p110α*. Increasing hepatic levels of miR-378/378* in ob/ob mice improves hepatic steatosis without exacerbating fasting hyperglycemia [113]. Furthermore, miR-378 has been shown to prevent and treat obesity in mice by activating the pyruvate-phosphoenolpyruvate (pyruvate-PEP) futile cycle in muscle tissue and enhancing lipolysis in adipose tissue [114]. The expression of miR-378 increases in the livers of mice and human hepatocytes with accumulated lipids. MiR-378 targets nuclear receptor factor 1 (*Nrf1*), which acts as a transcription repressor of miR-378. The negative feedback loop between miR-378 and *Nrf1* is associated with the pathogenesis of NAFLD [115]. Hepatic miR-378 regulates the expression of key enzymes in bile acid synthetic pathways via *MAFG*. This modulates bile acid and cholesterol metabolism [116].

MiR-483-5p targets *PCSK9*, thereby influencing cholesterol homeostasis. This increases LDLR expression, thereby decreasing LDL-C levels and the prevalence of cardiovascular events. Overexpression of miR-483 in hypercholesterolemic mouse models has been shown to significantly reduce plasma cholesterol and LDL-C levels [117]. Post-natal overexpression of miR-483 leads to stunted growth by repressing *IGF1* and increasing hepatic lipid production. This results in excessive adiposity [118].

Several targets are shared by miR-520d and miR-224, including *PCSK9* and *IDOL*, both of which are involved in LDLR degradation. In vitro studies using hepatocyte cell lines have demonstrated that both miRNAs reduce the levels of *PCSK9*, as well as *IDOL* and *HMGCR*. This increases LDLR expression and LDL binding [107].

Hsa-miR-613 targets *LXRα*, thereby reducing its expression. Hsa-miR-613 expression can be transcriptionally activated by *LXRα*, a process mediated by *SREBP-1c*, which is a known *LXRα* target gene [119]. In HepG2 cells, miR-613 suppresses lipogenesis by directly targeting *LXRα* [120]. Furthermore, an inverse correlation has been observed between miR-613 and LXRα/ABCA1 in PPARγ-activated macrophages. Treatment with miR-613 downregulates *LXRα* and *ABCA1*, thereby inhibiting cholesterol efflux [121].

The expression of miR-758 is reduced by the amount of cholesterol in the cells of macrophages and in the livers of mice fed a high-fat diet. It regulates cholesterol efflux by post-transcriptionally repressing *ABCA1* [122]. Furthermore, miR-758-5p reduces the accumulation of lipids in foam cells by regulating the uptake of cholesterol via *CD36* [123]. Glucagon-like peptide-1 increases *ABCA1* expression by downregulating miR-758, thereby regulating cholesterol homeostasis [124].

In summary, miRNAs play a key role in the regulation of lipid metabolism. They act as key regulators of this process by precisely targeting the genes involved. They bind to the 3′ UTR of target genes, inducing gene degradation or translational repression and affecting downstream regulatory networks, thereby ultimately influencing lipid metabolism. For instance, miR-370 directly targets the 3′ UTR of *CPT1A*, thereby reducing the rate of fatty acid β-oxidation.

### 3.2. Role of lncRNAs in Lipid Metabolism

**Figure 5:**
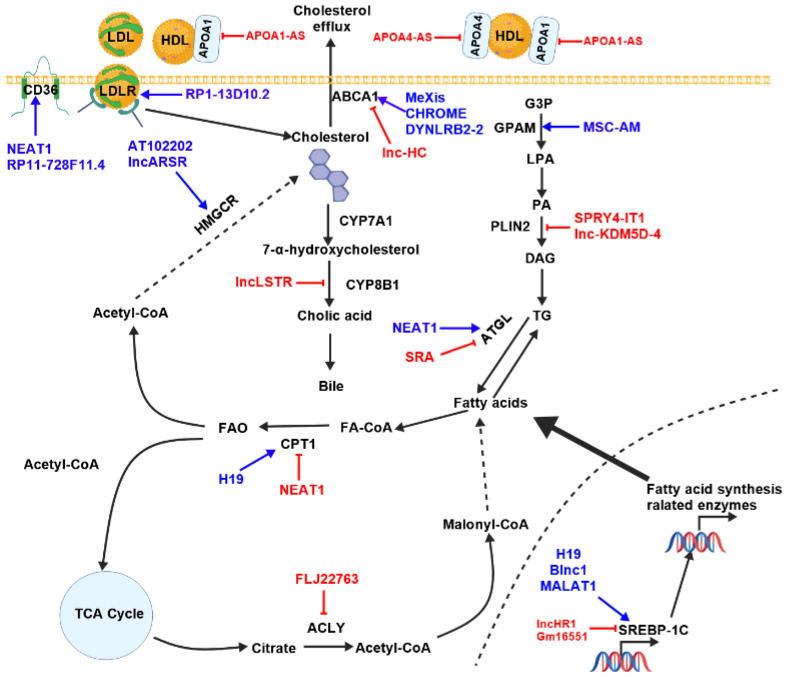
The summary of the potential functions of lncRNAs in lipid metabolism. Blue arrows indicate activation of the pathway, red symbols indicate blocking of the pathway. ACLY (ATP Citrate Lyase), ATGL (adipose triglyceride lipase), CPT1 (Carnitine palmitoyltransferase1), CYP7A1 (cholesterol 7α- hydroxylase), HMGCR (HMG-CoA reductase), LDL (low-density lipoprotein), LDLR (low-density lipoprotein receptor), LPL (high-density lipoprotein), FA-CoA (Fatty acid-CoA), GPAM (Glycerol-3-phosphate acyltransferase, mitochondrial), LPA (lisophospha-tidic acid), PA (phosphatidic acid), PLIN2 (Perilipin 2), TAG (triacyl glycerols), TG (triglyceride), TCA (tricarboxylic acid).

Several studies have demonstrated that lncRNAs play crucial roles in lipid metabolism (Figure 5).

ApoA1-AS is a lncRNA located in the apolipoprotein (APO) gene cluster. This cluster contains several APO genes, including *ApoA1*, *ApoA4*, *ApoA5*, and *ApoC3*. It negatively regulates *ApoA1* expression by controlling the methylation of histones in regions of chromatin that flank the *ApoA1* gene. By influencing the binding of LSD1 and SUZ12 to the APO gene cluster, ApoA1-AS epigenetically modifies the cluster and inhibits the expression of *ApoA1*, *ApoA4*, and *ApoC3* [125].

Similar to *ApoA1*, the expression of *ApoA4* is regulated by its antisense lncRNA APOA4-AS. APOA4-AS interacts directly with HuR (Hu antigen R or ELAV-like protein 1), which stabilizes *APOA4* mRNA. Silencing APOA4-AS in living organisms did not affect liver triglyceride levels, but significantly reduced triglyceride and cholesterol levels in the blood [126].

The lncRNA AT102202 is located in the *HMGCR* gene locus. Epigallocatechin-3-gallate (EGCG), which is found in green tea, can increase AT102202 expression while reducing *HMGCR* expression. AT102202 controls *HMGCR* expression, thereby influencing cholesterol accumulation in HepG2 cells [127].

Blnc1 (Brown fat-enriched long non-coding RNA 1) was first identified in adipose tissue. It plays a key role in regulating the differentiation and thermogenesis of brown adipose tissue [128,129,130]. The hepatic expression of Blnc1 is significantly increased in obese and NAFLD mice. Blnc1 promotes the assembly of the LXR transcription complex by binding to endothelial differentiation-related factor 1 (EDF1). Disrupting Blnc1 weakens the interaction between EDF1 and LXR, reducing LXR recruitment to the *SREBP-1c* promoter. This decreases the expression of genes involved in lipid synthesis [131].

CDKN2B-AS1 (cyclin-dependent kinase inhibitor 2B antisense RNA 1) is known to play a crucial role in various diseases, including atherosclerosis, diabetes, and cancer. Research has revealed that the expression of CDKN2B-AS1 is reduced in atherosclerotic plaque tissue and THP-1 macrophage-derived foam cells. CDKN2B-AS1 can bind to DNA methyltransferase 1 (DNMT1), which in turn enhances the methylation of the *ADAM10* promoter and promotes cholesterol efflux [132].

CHROME (Cholesterol-induced regulator of metabolism RNA) is a primate-specific lncRNA. Elevated levels of CHROME expression are frequently observed in the plasma and arterial plaques of patients with cardiovascular disease. Interestingly, knocking down CHROME increases the levels of miR-27b, miR-33a, miR-33b, and miR-128, whereas reducing the expression of *ABCA1*, a gene repressed by these miRNAs. This disrupts cholesterol efflux and HDL biogenesis [133].

The expression of DYNLRB2-2 in human macrophages is induced by oxidized LDL (Ox-LDL). The expression of *ABCA1* and *GPR119* is increased by the upregulation of DYNLRB2-2 in macrophage foam cells. This process promotes cholesterol efflux and reduces the accumulation of neutral lipids [134]. Furthermore, DYNLRB2-2 enhances cholesterol efflux by reducing *TLR2* expression, thereby increasing ABCA1 protein expression [135].

Knockdown of ENST00000416361 was found to significantly reduce levels of IL-6 and TNF-α, both of which are strongly associated with the progression of coronary artery disease (CAD). This also resulted in decreased levels of *SREBP1* and *SREBP2*, which play an important role in lipid synthesis [136].

The lncRNA ENST00000602558.1 is differentially expressed in individuals with CAD compared to healthy controls. Overexpression of ENST00000602558.1 reduces *ABCG1* expression, whereas knocking down ENST00000602558.1 has the opposite effect. Consistently, overexpression of ENST00000602558.1 resulted in a 30.38% reduction in ABCG1-mediated cholesterol efflux to HDL from vascular smooth muscle cells (VSMCs), whereas knockdown of ENST00000602558.1 increased ABCG1-mediated cholesterol efflux by 30.41%. In addition to affecting cholesterol efflux, ENST00000602558.1 overexpression increases lipid accumulation and TC/TG levels in VSMCs, whereas knockdown decreases them. Furthermore, ENST00000602558.1 regulates *ABCG1* expression and ABCG1-mediated cholesterol efflux from VSMCs by binding to p65 [137].

The lncRNA GAS5 (growth-arrest-specific 5) is crucial for the development of atherosclerosis. Overexpression of GAS5 increased levels of TC, LDL, cholesterol ester (CE), and free cholesterol (FC) in ApoE^−/−^ mice with atherosclerosis. Knocking down GAS5 stimulates reverse cholesterol transport and reduces lipid accumulation, inhibiting the transcriptional repression of *ABCA1* by EZH2. This prevents the progression of atherosclerosis [138].

The lncRNA Gm16551 is significantly downregulated in the livers of starved mice. This is restored after refeeding. Gm16551 is robustly induced by the lipogenesis master transcription factor *SREBP1c*, after which it functions to negatively regulate the SREBP1c-induced lipogenesis pathway in hepatocytes and the liver. The expression of *ACLY*, *FAS*, and *SCD1* increases following intervention in Gm16551 in mice. Knocking down *SREBP1c* suppresses the levels of genes associated with lipid synthesis following intervention in Gm16551. Lipid synthesis is activated by SREBP-1c [139].

H19 RNA interacts with PTBP1, which facilitates its binding to SREBP-1c mRNA and protein. This increases the transcriptional activity of *SREBP-1c* [140]. H19 binds to miR-130a, which counteracts its inhibitory effect on *PPARγ* and promotes hepatic lipid synthesis [141]. H19 promotes hepatic steatosis. This is achieved through the upregulation of both mTORC1 and MLXIPL [142].

Microarray analysis and RT-PCR revealed that HOXC cluster antisense RNA 1 (HOXC-AS1) and homeobox C6 (HOXC6) are expressed at lower levels in human atherosclerotic plaques than in normal intima tissue. HOXC-AS1 promotes *HOXC6* gene expression and inhibits cholesterol induced by Ox-LDL [143].

The lncRNA LASER (Lipid Associated Single Nucleotide Polymorphism Gene Region) is initiated by LXR. LASER then binds to LSD1, which results in the demethylation of H3K4me at the *HNF-1α* promoter, thereby activating HNF-1α expression. Subsequently, HNF-1α promotes PCSK9 expression. Increased PCSK9 levels reduce the liver’s ability to remove cholesterol from the blood, leading to hypercholesterolemia [144].

Linc-GALNTL6-4 is an adipocyte-specific lncRNA, with expression being lower in obese individuals. While the expression of linc-MALAT1 is compromised in obese subjects due to inflammation, adipogenesis requires the transcriptional upregulation of linc-GALNTL6-4. Its expression peaks in terminally differentiated adipocytes. Knockdown of linc-GALNTL6-4 inhibits adipogenesis by inducing lipidome alterations and downregulating genes related to the cell cycle. This process also promotes inflammation, impaired fatty acid metabolism. Conversely, increased linc-GALNTL6-4 improves differentiation and the adipocyte phenotype, potentially by restraining APOC1 and promoting triglyceride metabolism [145].

Patients suffering from hypercholesterolemia, as well as mice fed a high-cholesterol diet, have higher levels of lncARSR. Overexpression of lncARSR increases the expression of cholesterol-related genes, including *HMGCR*, *HMGCS*, and squalene synthase (*SQS*). Further studies have demonstrated that lncARSR enhances SREBP-2 expression, thereby promoting cholesterol synthesis via activation of the PI3K/Akt signaling pathway [146].

The lncRNA lnc-KDM5D-4 locus is located on the Y chromosome. Under expression of this lncRNA in HepG2 cells leads to the overexpression of PLIN2 and increased lipid droplet formation [147].

The level of lncRNA lncSHGL (lncRNA suppressor of hepatic gluconeogenesis and lipogenesis) is notably high in liver tissue. The expression levels of the mouse lncRNA SHGL are reduced in the livers of obese mice. Similarly, levels of the human homolog B4GALT1-AS1 are reduced in the livers of patients with NAFLD. LncSHGL recruits hnRNPA1 to increase the translation of calmodulin, thereby activating the PI3K/Akt pathway and repressing the mTOR/SREBP1c pathway. This leads to the suppression of gluconeogenesis and lipogenesis [148].

Liver X Receptor (LXR) plays a key role in regulating cholesterol metabolism in the body. It promotes cholesterol efflux and esterification, as well as inhibiting cholesterol absorption [149]. In mouse models subjected to either a Western diet or pharmacological LXR activation, the expression of the liver-specific lncRNA LeXis, which depends on LXR, was found to be significantly increased. This resulted in decreased cholesterol levels in the serum and liver. In the presence of high cholesterol levels, LXR activates the expression of LeXis in the liver. This reduces cholesterol synthesis by stopping RNA polymerase II from being recruited to Srebf2 and its transcriptional targets. LeXis is predominantly localized in the nucleus and is associated with the heterogeneous ribonucleoprotein RALY, which has previously been linked to SREBP-2. RALY acts as a transcriptional coactivator that promotes the expression of genes involved in cholesterol biosynthesis. LeXis combines with RALY to inhibit its transcriptional regulatory function, thereby decreasing the production of cholesterol [150].

LIPTER (lipid droplet transporter) plays a crucial role in transporting lipid droplets (LDs) within human cardiomyocytes. It has been found that a deficiency of LIPTER impairs the transport and metabolism of LD, resulting in impaired mitochondrial function and reduced cardiomyocyte viability. LIPTER binds to both PA and PI4P, facilitating LD transfer. LIPTER also binds to the MYH10 protein, which connects LDs with the cytoskeleton, thereby promoting LD transport. Overexpression of LIPTER reduces cardiac lipotoxicity and protects cardiac function, mitigating cardiomyopathies in mice fed a high-fat diet and in Lepr^db/db^ mice [151].

The expression of lnc-HC is induced by the accumulation of cholesterol. Lnc-HC then forms a complex with hnRNPA2B1, which inhibits the expression of *ABCA1* and consequently the reverse cholesterol transport (RCT) process [152]. Lnc-HC regulates hepatic lipid metabolism mediated by *PPARγ* via miR-130b-3p; however, there is a positive correlation between lnc-HC and its expression [153].

The expression of lncHLEF is significantly higher in the livers of peak-laying hens than in those of pre-laying hens. LncHLEF promotes hepatocyte lipid droplet formation and increased levels of triglycerides and total cholesterol. LncHLEF acts as a competing endogenous RNA, modulating the miR-2188-3p/GATA6 axis. It also encodes three small functional polypeptides that directly interact with the ACLY protein, stabilizing it. Notably, overexpression of lncHLEF promotes hepatic lipid synthesis and intramuscular fat deposition, but does not affect abdominal fat deposition. Furthermore, hepatocyte-derived lncHLEF can be delivered to preadipocytes in muscle and abdominal tissue via exosomes secreted by hepatocytes. This process enhances the differentiation of intramuscular preadipocytes without impacting the differentiation of those in the abdominal region [154].

LncHR1 (HCV-regulated 1) is a lncRNA that can be activated by the hepatitis C virus (HCV). The overexpression of lncHR1 inhibits the expression of *SREBP-1c* and *FAS*, both of which are essential for lipogenesis. This subsequently suppresses triacylglycerol (TG) and lipid droplet accumulation in hepatocytes. In vivo, transgenic mice expressing lncHR1 exhibited reduced hepatic expression of *SREBP-1c*, *FAS*, and *ACCα*, as well as decreased hepatic and plasma TG levels, when fed a high-fat diet (HFD) [155]. In the process of HCV promoting liver lipid synthesis, lncHR1 did not facilitate this development; instead, it reduced lipid accumulation by inhibiting the expression of *SREBP-1c*. This inhibitory effect is mediated by the regulation of the pyruvate dehydrogenase kinase 1 (PDK1)/AKT/FoxO1 pathway [156].

LncLSTR is a liver-specific lncRNA. Its expression diminishes during fasting, whereas substantial upregulation is observed upon refeeding. LncLSTR was found to bind to the nucleic acid-binding protein TDP-43. This reduces the inhibitory effect of TDP-43 on Cyp8b1 expression. Inhibiting lncLSTR decreases Cyp8b1 expression, which alters the body’s bile acid ratio and activates the bile acid receptor FXR. This promotes the expression of Apoc2, which is a positive regulator of LPL. Increased Apoc2 expression boosts the activity of lipolytic enzymes. This increases the rate at which triglycerides are cleared from the blood [157].

Treatment with oleic acid (OA) and palmitic acid increased the expression of LncNONMMUG027912 (Lnc027912) in the AML12 murine hepatocyte cell line. This increased expression resulted in significant reductions in lipid accumulation and triacylglycerol levels, as well as decreased expression of genes involved in lipid biosynthesis. LncNONMMUG027912 increases the expression of p-AMPKα and reduces the levels of p-mTOR. It also decreases *SREBP-1c* expression in the nucleus and its promoter activity, and inhibits the expression of genes involved in lipid synthesis. In a mouse model of NAFLD, the accumulation of lipids and liver inflammation can be reduced by LncNONMMUG027912, via the AMPKα/mTOR signaling axis [158].

LncRHPL (long non-coding RNA regulator of hyperlipidaemia) is mainly found in the liver, and its expression is reduced in mice fed a HFD and in primary hepatocytes treated with oleic acid. LncRHPL binds directly to hnRNPU, enhancing its stability and enabling it to activate Bmal1 transcription. This, in turn, inhibits the secretion of VLDL in hepatocytes. LncRHPL deficiency promotes the degradation of the hnRNPU protein, without affecting its mRNA level. This suppresses the expression of Bmal1 and other genes involved in hepatic VLDL secretion [159].

The lncRNA LOC286367 and *ABCA1* are located on the same chromosome, but undergo transcription in opposite directions. Overexpression of LOC286367 inhibits *ABCA1* expression. This leads to lipid accumulation [160].

The expression of MALAT1 (metastasis-associated lung adenocarcinoma transcript 1) increases in hepatocytes when they are exposed to palmitate, and also in the livers of ob/ob mice. Inhibiting MALAT1 reduces the stability of the nuclear SREBP-1c protein in hepatocytes, thereby suppressing hepatic lipid accumulation and attenuating hepatic steatosis [161]. MALAT1 also regulates cholesterol efflux via the MALAT1-miR-17-ABCA1 axis. Knocking down the MALAT1 gene promotes the accumulation of cholesterol in Ox-LDL [162].

The lncRNA MEG3 (Maternal Embryonic Lethal-Like 3) binds to PTBP1, recruiting it to the small heterodimeric partner (*SHP*) mRNA. This process promotes the degradation of SHP mRNA, resulting in weakened bile acid homeostasis. Conversely, SHP inhibits the activation of MEG3 expression mediated by CREB in a feedback-regulatory manner [163]. MEG3 competitively binds to miR-21, thereby inhibiting the mTOR pathway and inducing intracellular lipid accumulation [164].

MeXis is induced by LXR in macrophages. MeXis is located in the nucleus and exercises control over the architecture of chromosomes within the *ABCA1* locus. MeXis interacts directly with DDX17, an established nuclear receptor coactivator, which is enriched at the LXR binding sites in the *ABCA1* enhancer region in macrophages. Higher MeXis levels subsequently amplify *ABCA1* expression, enhancing macrophage cholesterol efflux. It was found that mice lacking the MeXis gene exhibited significantly lower tissue-specific expression of *ABCA1*. This led to a twofold increase in the likelihood of vascular occlusion compared to normal mice. Of note, a similar transcript (TCONS_00016111) is located near the *ABCA1* locus in humans and influences *ABCA1* levels and cholesterol efflux when overexpressed or inhibited in the THP-1 cell line. These observations are consistent with the substantial correlation between a SNP that overlaps the TCONS_0001611 transcript and coronary disease. This suggests that the LXR–MeXis–ABCA1 axis may be involved in regulating macrophage sterol metabolism in humans [165].

The lncRNA MSC-AS interacts with miRNA-33b-5p, thereby promoting the expression of glycerol-3-phosphate acyltransferase, mitochondrial (*GPAM*), and facilitating the synthesis of triglycerides, which in turn promotes lung adenocarcinoma [166].

The lncRNA NEAT1 (nuclear paraspeckle assembly transcript 1) increases *ATGL* levels by binding to miR-124-3p, thereby disrupting lipolysis in HCC cells [167]. NEAT1 can also promote the expression of *ACC* and *FAS* in hepatocytes by activating the mTOR/S6K1 signaling pathway [168]. Oxidized low-density lipoprotein triggers the formation of paraspeckles via NEAT1 in the THP-1 human monocyte cell line. The process of cholesterol uptake is partially suppressed by paraspeckles. This is achieved by the stabilization of *CD36* mRNA and the inhibition of its expression. [169]. Inhibiting NEAT1 in vitro can reduce the expression of miR-140. Conversely, inhibiting miR-140 can also reduce NEAT1 expression. However, interfering with either can increase AMPK phosphorylation and reduce the expression levels of the *SREBP1*, *FAS*, and *ACC* genes, thereby playing a role in lowering lipids [170]. In the HepG2 cell line, NEAT1 acted as a sponge for miR-146a-5p, thereby activating Rho-kinase isoform 1, decreasing *CPT1* expression, and facilitating lipid accumulation [171]. NEAT1 is crucial for the development of atherosclerosis. Blocking it decreases the uptake of lipids by THP-1 human macrophages [172]. Rapamycin promotes NEAT1 expression. NEAT1 then sponges has-mir-372-3p, which induces the expression of *APOG4* in both the Huh7 and HepG2 cell lines, as well as in mouse livers. This process facilitates the accumulation of triglycerides and hypertriglyceridemia [173].

NFIA antisense RNA 1 (NFIA-AS1/RP5-833A20.1) is found in intron 2 of the *NFIA* gene. In human THP-1 macrophage-derived foam cells, Ox-LDL or acetylated low-density lipoprotein (Ac-LDL) increases the expression of NFIA-AS1. NFIA-AS1 then triggers the expression of hsa-miR-382-5p, which leads to the upregulation of *NF1A*. *NF1A* is a key regulator of lipid homeostasis and adipocyte differentiation. Decreased *NF1A* reduces reverse cholesterol transport (RCT) and affects cholesterol homeostasis [174].

The lncRNA RP1-13D10.2 increases the expression of *LDLR*, thereby enhancing LDL-C uptake in the plasma of human hepatocyte cell lines (Huh7 and HepG2). Furthermore, patients with a high response to LDL-C treatment with statins had increased levels of RP1-13D10.2. Interestingly, a SNP (rs6924995) within RP1-13D10.2 has been linked to responses to statin therapy in terms of both LDL-C levels and expression levels [175].

The lncRNA RP11-728F11.4 has been linked to levels of the CD36 protein in human monocyte-derived macrophages. RP11-728F11.4 binds to the RNA recognition domain of the EWSR1 protein, thereby alleviating its repressive activity and enabling FXYD6 expression. This interaction increases FXYD6 levels, which subsequently stimulate CD36 expression and lead to intracellular cholesterol accumulation [176].

Increased levels of lncRNA SPRY4-IT1 have been observed in the A375 melanoma cell line. It binds directly to LPIN2, thereby downregulating triglyceride production and inducing cellular lipotoxicity [177].

The lncRNA SRA (steroid receptor RNA activator) promotes lipid metabolism by suppressing the promoter activity of *ATGL* through targeting the transcription factor FoxO1. This downregulates *ATGL* expression, thereby promoting hepatic steatosis [178].

The lncRNA TUG1 is strongly associated with diabetes and related diseases. TUG1 impairs the expression of *ApoM* by competing with FXR1 for the regulation of the expression of miR-92a. This inhibits RCT and promotes the progression of atherosclerosis [179]. Downregulation of TUG1 mitigates the dysfunction caused by ox-LDL by impairing cell proliferation and inflammation, while encouraging apoptosis in mouse VSMCs and macrophages. This process was achieved by regulating the miR-133a/FGF1 axis [180].

The expression of Uc.372 increases in the livers of db/db mice, mice fed a high-fat diet, and patients with NAFLD. It binds to pri-miRNA-195/4668, thereby inhibiting the maturation of miRNA-195/4668 and regulating the expression of genes involved in lipid synthesis and uptake. These genes include *ACC*, *FAS*, *SCD1*, and *CD36* [181].

In summary, lncRNAs play critical roles in regulating lipid metabolism. LncRNAs control lipid metabolism through multifaceted epigenetic mechanisms. Some lncRNAs scaffold chromatin-modifying complexes to specific genomic loci, such as ADINR. ADINR recruits MLL3/4 to deposit H3K4me3 at the *C/EBPα* promoter, creating an open chromatin configuration permissive for transcription. Some lncRNAs stabilize mRNAs of lipid-synthesis enzymes, such as MALAT1. MALAT1 enhances *SREBP-1c* mRNA stability, amplifying hepatic triglyceride and cholesterol synthesis. Some lncRNAs act as competing endogenous RNAs (ceRNAs) by sequestering microRNAs, such as H19. H19 binds to miR-130a, which counteracts its inhibitory effect on *PPARγ*, thereby promoting hepatic lipid synthesis. Some lncRNAs orchestrate lipid metabolism through chromatin remodeling, such as GAS5. GAS5 overexpression inhibits the enhancer of EZH2-mediated *ABCA1* expression via histone methylation in THP-1, thereby increasing lipid accumulation.

### 3.3. Role of circRNAs in Lipid Metabolism

**Figure 6:**
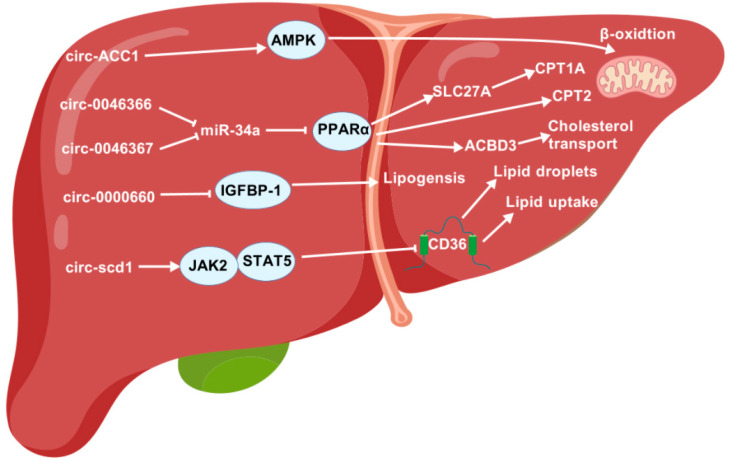
The regulatory roles of circRNAs in lipid metabolism. ACBD3, acyl-CoA-binding domain-containing 3; AMPK, AMP-activated protein kinase; CPT1A, carnitine palmitoyltransferase 1A; CPT2, carnitine palmitoyltransferase 2; IGFBP-1, insulin-like growth factor-binding protein-1; JAK2, Janus kinase 2; LD, lipid droplet; PPARα, peroxisome proliferator-activated receptor α; SLC27A, solute-carrier family 27A; STAT5, signal transducer and activator of transcription 5.

Research has demonstrated the involvement of circRNAs in the process of lipid metabolism (Figure 6).

Under serum deprivation conditions, the transcription factor c-Jun induces the expression of CircACC1 more readily than *ACC1*. Further research shows that circACC1 enhances the assembly and activity of the AMPK holoenzyme by forming a ternary complex with the regulatory β- and γ-subunits. This process accelerates β-oxidation and glycolysis [182].

CircFUT10 is significantly more abundant in adult subcutaneous fat than in the calf group. Further research has shown that circFUT10 binds to let-7c, thereby promoting cell proliferation and hindering differentiation in cattle adipocytes by targeting *PPARGC1B* [183].

It has been shown that circH19 inhibits lipogenesis via the PTBP1/SREBP1 pathway in hADSCs. Knocking down circH19 increases the expression of adipogenic genes (including *PPARγ*, *CEBPα*, and *SREBP-1c*) and the formation of lipid droplets [184].

CircHIPK3 is significantly upregulated in HepG2 cells when treated with oleate. This enhances oleate’s stimulatory effect on lipid droplet accumulation. Additionally, circHIPK3 targets miR-192-5p. *FOXO1* is a downstream regulator of miR-192-5p, whereas circHIPK3 significantly increases *FOXO1* expression. Furthermore, *FOXO1* affects the expression of *SREBP-1c* and *FAS*, both of which are involved in adipogenesis [185].

In AML12 cells, inhibition of circRNA_0000660 has been shown to increase lipid accumulation and reduce *IGFBP-1* mRNA and protein levels. *IGFBP-1* plays a role in obesity resistance and lipid metabolism. CircRNA_0000660 targets miR-693. Combined inhibition of circRNA_0000660 and miRNA 693 reduced lipid accumulation and increased *IGFBP-1* levels [186].

CircRNA_0046367 is significantly downregulated in HepG2 cells when exposed to high levels of fat. Further research shows that restoring circRNA_0046367 abolishes the inhibitory effect of miR-34a on *PPARα* by blocking the interaction between microRNA and mRNA with microRNA response elements (MREs). Restoring PPARα activates genes involved in lipid metabolism, such as *CPT2* and *ACBD3*. This subsequently resolves steatosis [187]. Another study shows that miR-33a is a direct target of circFASN (also known as circRNA_0046367). The study also found that overexpressing circFASN or silencing miR-33a decreases tacrolimus’s ability to promote triglyceride accumulation [188]. Additionally, circRNA_0046366 terminates the inhibition of *PPARα* by miR-34a and activates the expression of downstream genes such as *CPT1A* and the solute carrier family 27 member 1 (*SLC27A1*) proteins, which play a role in triglyceride breakdown. Rebalancing lipid homeostasis in this way led to a dramatic reduction in triglyceride content and an improved phenotype of hepatocellular steatosis [189].

The expression of circRNA_021412 is significantly reduced following high-fat stimulation. Reduced circRNA expression impairs its ability to competitively inhibit miR-1972. Consequently, reactivated miR-1972 decreases the transcriptional and/or translational levels of LPIN1, inducing steatosis-related gene expression via *PPARα* [190].

CircSAMD4A is significantly more abundant in obese individuals than in lean individuals. Further research has demonstrated that circSAMD4A promotes adipogenesis by activating the miR-138-5p/EZH2 pathway [191].

Mice fed a high-fat diet had significantly lower levels of CircScd1 expression in their livers compared to those fed a control diet. Overexpression of circScd1 inhibits the formation of lipid droplets and increases JAK2 and STAT5 protein levels. JAK2 and STAT5 both play a key role in lipid metabolism [192].

In summary, circRNAs play key roles in regulating lipid metabolism by acting as epigenetic master regulators. CircRNAs absorb microRNAs, such as circRNA_021412, which regulates lipid metabolism by absorbing miR-1972. Reduced expression of circRNA_021412 reduces its ability to competitively inhibit miR-1972. Consequently, reactivated miR-1972 induces reductions in the transcriptional and/or translational levels of LPIN1, thereby inducing the expression of steatosis-related genes via the activation of the *PPARα*. CircRNAs also act as epigenetic master regulators of lipid metabolism by scaffolding protein complexes, such as circRNA_0046367. Restoring circRNA_0046367 eliminates the inhibitory effect of miR-34a on *PPARα* by preventing the interaction between microRNA and mRNA via microRNA response elements (MREs). This subsequently leads to the transcriptional activation of genes involved in lipid metabolism, such as *CPT2* and *ACBD3*. Ultimately, this results in the resolution of steatosis.

## 4. Therapeutic Potentials of ncRNAs

Analyzing the concentrations of lipid components in the blood, such as total cholesterol, HDL, LDL, and triglycerides, can help diagnose lipid-related diseases and associated risks [193,194]. However, this method requires 9–12 h of fasting to produce accurate results and only provides limited information. Therefore, more precise diagnostic measures are needed [195]. Therefore, it is necessary to search for better diagnostics and novel biomarkers for lipid-related diseases in order to overcome these disadvantages. NcRNA is currently being developed as a diagnostic and prognostic biomarker, with the aim of enabling more accurate and detailed diagnoses [196,197].

MiRNAs, such as miR-33 and miR-122, play a crucial role in regulating the metabolism of fatty acids and cholesterol. This suggests that they could be used as therapeutic biomarkers for obesity [53,60,84,198]. A study of an Egyptian cohort of patients, designed to evaluate the relationship between miR-33a and miR-122, obesity indices, and glycemic parameters, found that patients had significantly higher levels of these miRNAs in their serum than those in the control group. Statistically significant correlations were observed between both miR-33a and miR-122 and BMI, waist circumference, weight, height, total cholesterol, and triglyceride levels [199]. Consequently, serum miR-33a and miR-122 could serve as non-invasive biomarkers for diagnosing metabolic syndrome. One group of researchers introduced four synthetic microRNA mimics—namely, miR-27a-3p, miR-192, miR-122, and miR-27b-3p—into exosomes derived from lean mice. These microRNA expression levels are notably higher in exosome samples derived from obese mice [200].

The lean animals were given a standard diet and injected with modified exosomes. This resulted in the development of glucose intolerance. In mice that were fed a high-fat diet, knocking out miR-155 globally reduced insulin resistance and glucose intolerance [201]. Another potential therapeutic target involves studying animal models lacking adipocyte miR-34a. Such models exhibit resistance to obesity caused by a high-fat diet and are protected against insulin resistance [202]. Furthermore, reducing levels of miR-143-3p in mice through anti-miR-143-3p injection into the tail vein was found to mitigate obesity-induced insulin resistance by modulating the expression of the insulin-like growth factor-2 receptor (*Igf2r*) [203]. These findings together suggest that microRNAs, such as miR-143-3p, miR-192, miR-122, miR-27a-3p, miR-155, miR-34a, and miR-27b-3p, can be targeted using either miRNA mimics or antimiRs in order to postpone the development of metabolic syndrome in individuals who are overweight or obese. A recent analysis has clarified the functions of microRNAs such as miR-122, miR-223, miR-21, miR-194/192, miR-155, and miR-29 in regulating liver physiology and pathology, as well as their potential for targeting various liver diseases therapeutically. It also revealed that, while miR-122 and miR-223 hinder the development of NAFLD, miR-21 promotes its progression [204]. An increase in miR-223 was detected in hepatocytes due to their preferential uptake of extracellular vesicles (EVs) that were enriched in miR-223 and derived from neutrophils. Once internalized by the hepatocytes, the EV-derived miR-223 inhibited the expression of genes involved in hepatic inflammation and fibrosis. This played a beneficial role in slowing the progression of NAFLD [205]. Therefore, developing a small molecule to enhance the expression of miR-223 could be a promising NAFLD treatment strategy.

Several lncRNAs, including GYG2P1, H19, linc-GALNTL6-4, lncRNA-p19461, lncRNA-p21015, lncRNA-p5549, lncSHGL, MIST, ROIT, and SNHG9, are lower in fat tissue and other tissues taken from obese humans and animals. Conversely, Blnc1, linc-ADAL, Lnc-leptin, MALAT1, Plnc1, PVT1, RP11-20G13.3, and Uc. 372 are among the lncRNAs that are up-regulated in obese individuals [206]. The lncRNA H19 was found to be present at lower levels in the brown adipose tissue of obese mice. It was also found to be negatively correlated with BMI in humans. Overexpression of H19 was found to protect against diet-induced obesity in animals, increasing insulin sensitivity and mitochondrial synthesis [207]. The expression levels of the lncRNAs p19461, p21015, and p5549 were lower in obese patients than in the control group. Additionally, the levels of these three long non-coding RNAs (lncRNAs) in the blood were found to be strongly linked to BMI, waist circumference, and waist-to-hip ratio. Furthermore, circulating levels of lncRNA-p19461 were found to be negatively associated with insulin resistance, increasing with diet-induced weight loss [208]. The lncRNA MIST was found to be present at lower levels in peritoneal macrophages and adipose tissue macrophages taken from mice with diet-induced obesity. It was also found to be downregulated in the human stromal vascular fraction taken from the adipose tissue of obese, metabolically unhealthy donors, when compared to controls. Silencing MIST increased the expression of inflammatory genes and altered LDL uptake in macrophages. Conversely, forced overexpression of this lncRNA reduced the transcription of immune-related genes, both at baseline and in response to LPS, and diminished modified LDL uptake by macrophages. Human studies verified MIST downregulation by proinflammatory stimuli, as well as an inverse correlation between its expression and obesity and insulin resistance [209]. The lncRNA ROIT was found to be present at lower levels in the pancreatic islets of obese mice and the blood serum of obese type 2 diabetic patients. Its expression was inhibited by HNF1 homeobox B. Furthermore, overexpressing ROIT improved insulin secretion and glucose homeostasis. This demonstrates that the link between obesity and β-cell dysfunction is mediated by ROIT [210]. Adipocyte-derived exosomal SNHG9 is down-regulated in obese individuals with endothelial dysfunction. SNHG9 can prevent endothelial dysfunction in obese patients by reducing inflammation and apoptosis [211]. Increased expression of the lncRNA Lnc-leptin was found in the fat tissue of ob/ob mice. Lnc-leptin exhibits a high correlation with *leptin* expression. Leptin is a hormone that regulates energy balance and body weight. Studies in vivo and in vitro have revealed that Lnc-leptin regulates leptin expression, and that dysregulation of this process is significantly linked to obesity [212]. The lncRNA linc-ADAL is upregulated in the adipose tissue of obese individuals, with significant induction during in vitro human adipocyte differentiation. Further research has revealed that linc-ADAL interacts with hnRNPU and IGF2BP2 in different parts of the cell, thereby controlling adipocyte differentiation and lipogenesis [213]. The lncRNA PVT1 is expressed at high levels in adipose tissue. The expression levels of PVT1 are higher in obese mice than in non-obese mice. PVT1’s role in adipocyte differentiation has been confirmed; this lncRNA increases lipid accumulation by inducing the expression of *PPARγ*, *C/EBP-α*, and *aP2* [214]. The lncRNA RP11-20G13.3 was found to be present at lower levels in the adipose tissue of obese children. This was also positively correlated with waist circumference, waist-to-hip ratio, BMI, LDL-C, leptin, and fasting insulin levels. In contrast, downregulation of the lncRNA GYG2P1 was found to be negatively correlated with waist circumference, BMI, triglyceride levels, and fasting insulin levels [215]. Therefore, these lncRNAs can serve as diagnostic markers for the development of obesity therapies.

Deregulation of lncRNAs, including H19 [216], TUG1 [217], GAS5 [218], RAPIA [219], MIAT [220], CASC11 [221], NEXN-AS1 [222], and lnc00113 [223], has been observed in individuals with atherosclerosis. LncRNAs, including H19, TUG1, MIAT, and CASC11, can be detected in serum and could serve as potential diagnostic markers for atherosclerosis. In addition to identifying the functional role of lncRNAs in diagnosis, it has been discovered that certain lncRNAs, including AL117190.1, COL4A2-AS1, LINC00184, MEG3, and MIR22HG, could serve as important prognostic indicators for patients [224].

The lncRNA CHROME is present at higher levels in plasma and atherosclerotic plaques. Consequently, it has been proposed as a potential biomarker for the progression of coronary artery disease (CAD) [133]. In contrast, LeXis—a molecule found in plasma that plays a role in cholesterol metabolism and hepatic steatosis—has been identified as a non-invasive diagnostic biomarker for non-alcoholic steatohepatitis (NASH) [225]. Developing lncRNA biomarkers to diagnose lipid-related diseases is showing promise.

In addition, the progression of various cancers is influenced by lncRNAs, which can be used as diagnostic and prognostic markers. Examples include H19 [226], MEG3 [227], PVT1 [228], FAM83H antisense RNA 1 (FAM83H-AS1) [229], SNHG1 [230], and LUCAT1 [12]. Therefore, the dysregulated lncRNAs could serve as biomarkers for diagnosing and predicting lipid-related diseases, and for therapeutic applications.

Human visceral pre-adipocytes (HPA-v) exhibit high levels of hsa_circ_0017650, hsa_circ_0136134, and hsa_circRNA9227_1. This suggests that these circRNAs could be used as diagnostic and therapeutic targets for visceral obesity [231]. As mentioned above, circSAMD4A is an important molecule in the development of obesity. Knocking down circSAMD4A using siRNAs has been shown to significantly reduce food intake and lower body fat. This also reversed weight gain and improved energy expenditure, glucose tolerance, and insulin sensitivity in obese mice fed a high-fat diet. These findings clarify the pathological role of circSAMD4A, highlighting its potential as a therapeutic target for obesity treatment [191].

## 5. Conclusions

Multiple studies have investigated the regulatory role of ncRNAs in various biological processes associated with lipid metabolism, particularly focusing on miRNAs, lncRNAs, and circRNAs. These ncRNAs can influence gene expression in several ways by interacting with proteins, DNA, and RNA. Furthermore, many ncRNAs are dysregulated in metabolic tissues with abnormal lipid metabolism, which could contribute to the development of lipid-associated diseases. In order to identify novel therapeutic targets, it is essential to comprehend the role of ncRNAs in the molecular pathobiology of these diseases. This review will help scientists to explore the potential of ncRNAs for diagnosis and therapy in lipid-associated diseases.

## Data Availability

All the data are included in the manuscript.

## Supplementary Materials

The following supporting information can be downloaded at: https://www.mdpi.com/article/10.3390/ani15172621/s1, Table S1: MiRNAs are associated with adipogenesis; Table S2: LncRNAs are associated with adipogenesis; Table S3: CircRNAs are associated with adipogenesis; Table S4: MiRNAs are involved in lipid metabolism; Table S5: LncRNAs are involved in lipid metabolism; Table S6: CircRNAs are involved in lipid metabolism; Table S7: The proposed roles of ncRNAs as diagnostic and prognostic markers for obesity and dyslipidemia/lipid profile abnormalities.
