# Supplementary material for: Advances in the Regulation of Lipid Metabolism by Non-Coding RNAs
Source: Animals (Basel). 2025 Sep 7;15(17):2621. doi: 10.3390/ani15172621 (PMC12427337; doi:10.3390/ani15172621)
Supplement: Supplementary file 1 [file animals-15-02621-s001.zip › animals-3724499-supplementary.pdf]

**Supplemental Table S1.** MiRNAs are associated with adipogenesis

| MiRNA | Target | Function | Experiment model | References |
|---|---|---|---|---|
| **miR-8** | TCF | Proadipogenic | MSCs, ST2 | [1] |
| **miR-10b-5p** | APOL6 | Proadipogenic | 3T3-L1 | [2] |
| **miR-17-5p** | BMPR2, BMP2 | Proadipogenic | ADSCs | [3] |
| **miR-17-92** | Rb2/p130 | Proadipogenic | 3T3L1 | [4] |
| **miR-21** | TGFBR2 | Proadipogenic | MSC, 3T3-L1, hASCs | [5] |
| **miR-23b** | SESN3 | Proadipogenic | porcine preadipocytes | [6] |
| **miR-30** | RUNX2 | Proadipogenic | hMADS | [7] |
| **miR-103** | MEF2D | Proadipogenic | 3T3L1 | [8] |
| **miR-106a** | BMP2 | Proadipogenic | ADSCs | [3] |
| **miR-122-5p** | FABP5 | Proadipogenic | chicken abdominal preadipocytes | [9] |
| **miR-125a-3p** | FSTL1 | Proadipogenic | mouse subcutaneous preadipocyte | [10] |
| **miR-128-1** | KLF11 | Proadipogenic | SVFs | [11] |
| **miR-129** | | Proadipogenic | yak intramuscular preadipocytes | [12] |
| **miR-133a-3p** | | Proadipogenic | goat subcutaneous preadipocytes | [13] |
| **miR-140-5p** | PDGFR$\alpha$ | Proadipogenic | 3T3-L1 | [14] |
| **miR-143** | ERK5 | Proadipogenic | 3T3L1, MSCs | [15,16] |
| **miR-146a-5p** | ErbB4 | Proadipogenic | 3T3-L1 | [17] |
| **miR-146b** | SIRT1 | Proadipogenic | 3T3L1 | [18] |
| **miR-183** | LRP6 | Proadipogenic | 3T3L1 | [19] |
| **miR-196a** | MAP3K1 | Proadipogenic | goat intramuscular preadipocytes | [20] |
| **miR-204** | RUNX2, DVL3 | Proadipogenic | ADSCs | [21,22] |
| **miR-210** | TCF7L2 | Proadipogenic | 3T3L1 | [23] |
| **miR-214** | CPT2 | Proadipogenic | duck preadipocyte | [24] |
| **miR-302** | CDKN1A | Proadipogenic | ASDCs | [25] |
| **miR-302b** | CDK2 | Proadipogenic | bovine preadipocyte | [26] |
| **miR-330** | SESN3 | Proadipogenic | bovine intramuscular preadipocytes | [27] |
| **miR-335** | MEST | Proadipogenic | 3T3L1, MSCs | [28] |
| **miR-375** | ERK1/2 | Proadipogenic | 3T3L1 | [29] |
| **miR-378a-3p** | MAPK1 | Proadipogenic | 3T3L1 | [30] |
| **miR-422a** | MeCP2 | Proadipogenic | hBMSCs | [31] |
| **miR-450b-3p** | SIRT1 | Proadipogenic | porcine intramuscular and subcutaneous adipocytes | [32] |

| | | | | |
|---|---|---|---|---|
| **miR-448** | KLF5 | Proadipogenic | 3T3L1, MSCs | [33] |
| **miR-519d** | PPARα | Proadipogenic | primary human visceral preadipocytes | [34] |
| **miR-548d-5p** | PPARγ | Proadipogenic | hBMSCs | [35] |
| **miR-574-5p** | Hdac9 | Proadipogenic | 3T3L1 | [36] |
| **miR-669a-5p** | | Proadipogenic | 3T3L1, C3H10T1/2 | [37] |
| **Let-7** | HMGA2 | Antiadipogenic | 3T3L1 | [38] |
| **miR-14** | P38MAPK | Antiadipogenic | Adipose tissue | [39] |
| **miR-18b-3p** | ACOT13 | Antiadipogenic | chicken intramuscular preadipocytes | [40] |
| **miR-19a, miR-19b** | | Antiadipogenic | 3T3L1 | [41] |
| **miR-21** | PTEN | Antiadipogenic | BMSCs | [42] |
| **miR-22** | HDAC6 | Antiadipogenic | ADSCs | [43] |
| **miR-23b-3p** | PDE4B | Antiadipogenic | goat intramuscular adipocytes | [44] |
| **miR-24-3p** | ANXA6 | Antiadipogenic | chicken intramuscular preadipocytes | [45] |
| **miR-26a-5p** | ACSL3 | Antiadipogenic | porcine preadipocyte | [46] |
| **miR-27a** | PPARγ | Antiadipogenic | 3T3L1 | [47] |
| **miR-27b** | PPARγ | Antiadipogenic | hMADS | [48] |
| **miR-29** | AKT | Antiadipogenic | 3T3L1 | [49] |
| **miR-29c** | IGF1 | Antiadipogenic | BMSCs | [50] |
| **miR-31** | C/EBPα | Antiadipogenic | MSCs | [51] |
| **miR-33a** | IRS2 | Antiadipogenic | bovine preadipocytes | [52] |
| **miR-33b** | EBF1 | Antiadipogenic | PSPA | [53] |
| **miR-92a-3p** | APOL6 | Antiadipogenic | goat intramuscular preadipocytes | [20] |
| **miR-107** | APOC2 | Antiadipogenic | bovine primary adipocytes | [54] |
| **miR-128-3p** | FDPS | Antiadipogenic | chicken intramuscular adipocyte | [55] |
| **miR-130** | PPARγ | Antiadipogenic | human preadipocytes | [56] |
| **miR-130b** | | Antiadipogenic | rat intramuscular preadipocytes | [57] |
| | KLF3 | Antiadipogenic | goat intramuscular adipocyte | [58] |
| **miR-135a-5p** | APC | Antiadipogenic | 3T3L1 | [59] |
| **miR-136** | HSD17B12 | Antiadipogenic | ovine adipose-derived stromal vascular fractions | [60] |
| | PPARGC1B | Antiadipogenic | sheep preadipocytes | [61] |
| **miR-137** | CDC42 | Antiadipogenic | ADSCs | [62] |
| **miR-138** | EID-1 | Antiadipogenic | MSCs | [63] |
| **miR-139-** | NOTCH1, | Antiadipogenic | 3T3L1 | [64] |

| | | | | |
|---|---|---|---|---|
| **5p** | IRS1 | | | |
| **miR-145** | IRS1 | Antiadipogenic | porcine preadipocytes | [65] |
| **miR-149-5p** | ATP7A | Antiadipogenic | porcine intramuscular preadipocytes | [66] |
| **miR-155** | CREB | Antiadipogenic | 3T3L1 | [67] |
| | C/EBPβ | Antiadipogenic | Adipose tissue | [67] |
| **miR-196b-3p** | CD47 | Antiadipogenic | porcine intramuscular and subcutaneous adipocytes | [32] |
| **miR-223** | GPAM | Antiadipogenic | chicken intramuscular preadipocytes | [68] |
| **miR-224** | EGR2 | Antiadipogenic | 3T3L1 | [69] |
| **miR-320** | PI3K | Antiadipogenic | 3T3L1 | [70] |
| **miR-326** | C/EBPα | Antiadipogenic | MSCs | [51] |
| **miR-328-5p** | FAS | Antiadipogenic | hMSCs | [71] |
| **miR-363** | E2F3 | Antiadipogenic | ADSCs | [72] |
| **miR-365-3p** | FKBP5 | Antiadipogenic | bovine preadipocytes | [73] |
| **miR-369-3p** | | Antiadipogenic | sheep preadipocytes | |
| **miR-421** | FGF13 | Antiadipogenic | goat intramuscular preadipocytes | [74] |
| **miR-484** | SFRP1<br>MAP3K9 | Antiadipogenic | bovine preadipocytes, bovine intramuscular adipocytes | [75,76] |
| **miR-486-5p** | SIRT1 | Antiadipogenic | ADSCs | [77] |
| **miR-540** | PPARγ | Antiadipogenic | ADSCs | [78] |
| **miR-4769-3p** | USP18 | Antiadipogenic | 3T3-L1 | [79] |
| **miR-6402** | BMPR2 | Antiadipogenic | 3T3-L1 | [80] |
| **miR-6517** | PFKL | Antiadipogenic | bovine preadipocyte | [81] |
| **miR-10167-3p** | TCF7L1 | Antiadipogenic | bovine preadipocyte | [82] |

**Supplemental Table S2.** LncRNAs are associated with adipogenesis

| LncRNA | Target | Function | Experiment model | References |
|---|---|---|---|---|
| **AC092159.2** | TMEM18 | Proadipogenic | HPA-v | [83] |
| **AC092834.1** | DKK1 | Proadipogenic | hADSCs | [84] |
| **ACART** | | Proadipogenic | 3T3L1 | [85] |
| **ADINR** | C/EBPα | Proadipogenic | human MSCs | [86] |
| **BIANCR** | | Proadipogenic | bovine intramuscular adipocytes | [87] |
| **Blnc1** | EBF2 | Proadipogenic | primary preadipocytes from mice | [88,89] |

| | | | | |
|---|---|---|---|---|
| **BlncAD1** | MYH10 | Proadipogenic | bovine preadipocytes | [90] |
| **Gm15290** | miR-27b, PPARγ | Proadipogenic | murine primary preadipocytes, HEK293 | [91] |
| **HOTAIR** | PPARγ | Proadipogenic | primary preadipocytes from Human | [92] |
| **HoxA-AS3** | Ezh2, Runx2 | Proadipogenic | BMSCs | [93] |
| **HOXA11-AS1** | | Proadipogenic | hADSCs | [94] |
| **IMFlnc1** | miR-199a-5p | Proadipogenic | porcine intramuscular adipocyte | [95] |
| **IMFNCR** | miR-128-3p, miR-27b-3p | Proadipogenic | chicken intramuscular adipocyte | [96] |
| **KCNQ1OT1** | miR-138 | Proadipogenic | TSCs | [97] |
| **lnc210** | | Proadipogenic | buffalo intramuscular adipocytes | [98] |
| **lnc_000368** | | Proadipogenic | porcine intramuscular adipocytes | [99] |
| **LNC6302** | SLC22A16 | Proadipogenic | chicken abdominal adipocytes | [100] |
| **lnc13728** | ZBED3 | Proadipogenic | hADSC | [101] |
| **lncAD** | TXNRD1 | Proadipogenic | chicken IMF and AbF preadipocytes | |
| **lncCCPG1** | miR-93 | Proadipogenic | bovine adipocytes | [102] |
| **lncFABP4** | | Proadipogenic | buffalo intramuscular preadipocytes | [103] |
| **lncIMF1** | miR-187 | Proadipogenic | porcine intramuscular preadipocyte | [104] |
| **lncIMF2** | miR-217 | Proadipogenic | porcine intramuscular preadipocyte | [105] |
| **lncMYOZ2** | AHCY/MYOZ2 | Proadipogenic | porcine preadipocytes | [106] |
| **lnc-OAD** | β-catenin | Proadipogenic | 3T3L1 | [107] |
| **lnc-ORA** | | Proadipogenic | 3T3L1 | [108] |
| **lncPLAAT3-AS** | miR-503-5p/PLAAT3 | Proadipogenic | porcine primary preadipocytes | [109] |
| **lncRNA-Adi** | miR-449a | Proadipogenic | rat ADSCs | [110] |
| **lncSAMM50** | | Proadipogenic | 3T3L1, buffalo primary | [111] |

| | | | | |
|---|---|---|---|---|
| | | | adipocytes | |
| **lncSHGL** | miR-149/Mospd3 | | 3T3L1 | [112] |
| **LOC646762** | | Proadipogenic | BMSCs | [113] |
| **LYPLAL1-AS1** | DSP | Proadipogenic | hAMSCs | [114] |
| **MEK6-AS1** | NAT10/MEK6 | Proadipogenic | MSCs | [115] |
| **MIR31HG** | FABP4 | Proadipogenic | ADSCs | [116] |
| **MIR99AHG** | miR-29b-3p | Proadipogenic | 3T3L1 | [117] |
| **mLas-V3** | | Proadipogenic | OP9 pre-adipocyte cell | [118] |
| **MSTRG4710** | miR-29b-3p/IGF1 | Proadipogenic | rabbit preadipocytes | [119] |
| **MSTRG.12568.2** | FOXO3 | Proadipogenic | porcine preadipocytes | [120] |
| **NDUFC2-AS** | | Proadipogenic | buffalo primary adipocyte | [121] |
| **NEAT1** | PPARγ2 | Proadipogenic | 3T3L1, ADSCs | [122,123] |
| **Paral1** | RBM14 | Proadipogenic | 3T3L1 | [124] |
| **Plnc1** | PPAR-γ2 | Proadipogenic | ST2 cells, BMSCs | [125] |
| **PU.1 AS** | PU.1 | Proadipogenic | 3T3L1 | [126] |
| **PVT1** | STAT3 | Proadipogenic | 3T3L1 | [127] |
| **RP11-290L1.3** | | Proadipogenic | human preadipocytes-visceral | [128] |
| **RP11-142A22.4** | miR-587 | Proadipogenic | preadipocytes from visceral adipose tissue | [129] |
| **SERPINE1AS2** | PAI1 | Proadipogenic | bovine intramuscular adipocytes | [130] |
| **SlincRAD** | PPARγ | Proadipogenic | 3T3L1 | [131] |
| **SNHG1** | PTBP1 | Proadipogenic | mouse BMSCs | [132] |
| **SRA** | PPARγ | Proadipogenic | 3T3L1 | [133,134] |
| **AdipoQ AS** | AdipoQ | Antiadipogenic | mouse primary preadipocytes | [135] |
| **ADNCR** | miRNA-204 | Antiadipogenic | 3T3L1, ADSCs | [136] |
| **BADLNCR1** | GLRX5 | Antiadipogenic | Bovine primary pre-adipocytes | [137] |
| **CAAlnc1** | HuR | Antiadipogenic | C3H10 | [138] |
| **G8110** | | Antiadipogenic | BMSCs | [139] |
| **GAS5** | miR-21a-5p/PTEN, miR-18a/CTGF | Antiadipogenic | 3T3L1, MSCs | [140,141] |
| **GM13133** | | Antiadipogenic | mouse white pre-adipocytes | [142] |
| **H19** | miR-188 | Antiadipogenic | BMSCs | [143] |
| **HCG11** | miR-204-5p/SIRT1 | Antiadipogenic | hAdMSCs | [144] |

| | | | | |
|---|---|---|---|---|
| **lincRNA-ROFM** | miR-133b/AdipoQ | Antiadipogenic | porcine preadipocytes | [145] |
| **lncBNIP3** | | Antiadipogenic | bovine intramuscular preadipocyte | [146] |
| **lnc-FR332443** | Runx1 | Antiadipogenic | 3T3L1 | [147] |
| **lncIMF4** | | Antiadipogenic | porcine intramuscular preadipocyte | [148] |
| **lncRNA-NEF** | miR-155/PTEN | Antiadipogenic | ADSCs | [149] |
| **lnc-U90926** | | Antiadipogenic | 3T3L1 | [150] |
| **MEG3** | miR-140-5p, miR-217/Dkk3 | Antiadipogenic | ADSCs | [151,152] |
| **MIR221HG** | | Antiadipogenic | bADSCs | [153] |
| **NR_015556** | Wnt10b, β-catenin | Antiadipogenic | C3H10T1/2, 3T3L1 | [154] |
| **ROA** | PTX3-ERK | Antiadipogenic | MSC | [155] |
| **TCONS_00041960** | miR-204-5p, miR-125a-3p | Antiadipogenic | BMSCs | [156] |

**Supplemental Table S3.** CircRNAs are associated with adipogenesis

| CircRNA | miRNA/ Protein | Effector Target | Function | Experiment model | References |
|---|---|---|---|---|---|
| **circ_0006511** | miR-87 | CD36 | Proadipogenic | goat intramuscular preadipocyte | [157] |
| **circ_0006859** | miR-431-5p | ROCK1 | Proadipogenic | hBMSCs | [158] |
| **circ-ATXN2** | | | Proadipogenic | rat adipose tissue-derived stromal cells | [159] |
| **circBDP1** | miR-181b, miR-204 | SIRT1, TRARG1 | Proadipogenic | bovine preadipocytes, 3T3L1 | [160] |
| **circCDR1as** | miR-7-5p | WNT5B | Proadipogenic | BMSCs | [161] |
| **circDOCK7** | miR-301b-3p | ACSL1 | Proadipogenic | chicken abdominal preadipocytes | [162] |
| **circFLT1** | miR-93 | lncSLC30A9 | Proadipogenic | bovine adipocytes | [102] |
| **circMARK3** | | | Proadipogenic | buffalo adipocytes, 3T3-L1 | [163] |
| **circMAPK9** | miR-1322 | FTO | Proadipogenic | human primary preadipocytes | [164] |
| **circNDUFA13** | | STAT3 | Proadipogenic | BMSCs | [165] |
| **circ-PLXNA1** | miR-214 | CTNNB1 | Proadipogenic | duck preadipocytes | [166] |
| **circPPARA** | miR-429, | | Proadipogenic | porcine | [167] |

| | | | | | |
|---|---|---|---|---|---|
| | miR-200b | | | intramuscular preadipocytes | |
| **circPPARγ** | miR-92a-3p | YY1 | Proadipogenic | bovine primary adipocytes | [168] |
| **circRNF111** | miR-27a-3p | PPARγ | Proadipogenic | bovine preadipocyte | [169] |
| **circSAMD4A** | miR-138-5p | EZH2 | Proadipogenic | human preadipocyte | [170] |
| **circSETBP1** | miR-149-5p | CRTC1/ CRTC2 | Proadipogenic | Porcine intramuscular preadipocytes | [171] |
| **circTIAM1** | miR-485-3p | PLCB1 | Proadipogenic | sheep adipocytes | [172] |
| **circTshz2-1, circArhgap5-2** | | | Proadipogenic | primary mouse preadipocytes; human white adipocytes | [173] |
| **circ_0011446** | miR-27a-5p | FAM49 B | Antiadipogenic | goat intramuscular preadipocytes | [174] |
| **circADAMTS 16** | miR-10167-3p | | Antiadipogenic | bovine preadipocytes | [175] |
| **circBTBD7** | miR-183 | SMAD4 | Antiadipogenic | bovine primary adipocytes | [176] |
| **circFUT10** | let-7c | PPARG CIB | Antiadipogenic | bovine adipocytes | [177] |
| **circH19** | PTBP1 | SREBP1 | Antiadipogenic | hADSCs | [178] |
| **circHOMER1** | miR-23b | SIRT1 | Antiadipogenic | porcine preadipocytes | [179] |
| **circINSR** | miR-15/16 | FOXO1, EPT1 | Antiadipogenic | bovine intramuscular preadipocytes | [180] |
| | miR-152 | MEOX2 | Antiadipogenic | ovine SVFs | [181] |
| **circITGB1** | miR-23a | ARRB1 | Antiadipogenic | sheep adipocytes | [182] |
| **circITGB5** | miR-181b-5p | CPT1A | Antiadipogenic | chicken intramuscular preadipocytes | [183] |
| **circMEF2C(2, 3)** | miR-383/671-3p | MEF2C | Antiadipogenic | porcine intramuscular preadipocytes | [184] |
| **circPAPPA2** | miR-2366 | GK | Antiadipogenic | porcine preadipocytes | [185] |

**Supplemental Table S4.** MiRNAs are involved in lipid metabolism

| miRNA | Function | Targets | Reference |
|---|---|---|---|
| **miR-7** | Activates the expression of SREBP1 and SREBP2 to upregulate genes involved in sterol biosynthesis; controls cholesterol biosynthesis | SREBP1, SREBP2, DHCR24 | [186,187] |

| | | | |
|---|---|---|---|
| **miR-10b** | Regulates cellular steatosis levels by targeting PPAR-$\alpha$ expression; promotes β-adrenergic-induced lipolysis in adipocytes; mediates the trans-regulatory effect of the ABO locus on several blood proteins, coronary artery disease, and total cholesterol (TC) | PPAR-$\alpha$ | [188-190] |
| **miR-14** | Regulates the levels of organismal DAG and TAG | | [39] |
| **miR-21** | Blocks stearic acid (SA) induced intracellular lipid accumulation; targets PPAR$\alpha$; regulates lipid accumulation; promotes lipid and glucose metabolic disorders associated with high-fat diet consumption, inhibits the expression of LRP6 in HepG2 cells, thereby inducing lipid production; miR-21 antagomir improved insulin resistance and lipid metabolism disorder in STZ-induced T2DM rats; induces chicken hepatic lipogenesis; promotes differentiation and reduces oleic acid-induced lipid droplet accumulation in C2C12 myoblasts | FABP7, PPAR$\alpha$, TLR4-NF-κB, LRP6, TIMP3, NFIB, KLF3, FBXO11 | [42,191-197] |
| **miR-30c** | Reduces hyperlipidemia and atherosclerosis in mice by decreasing lipid synthesis and lipoprotein secretion; lowers plasma cholesterol and mitigates atherosclerosis by reducing microsomal triglyceride transfer protein expression and lipoprotein production and avoids steatosis by diminishing lipid synthesis; meliorates hepatic steatosis in leptin receptor-deficient (db/db) mice; circulating miR-30c is positively correlated with total- and LDL-cholesterol implicating regulatory functions in lipid homeostasis; reduces plasma cholesterol in homozygous familial hypercholesterolemic and type 2 diabetic mouse models; the miR122/30c ratio exhibits good association with MTTP, Apo B-48, and TG levels, and with chylomicron (CM) particle size | MTP, FAS | [198-203] |
| **miR-33** | Regulates cholesterol homeostasis, regulates cholesterol transport; involved in the β-oxidation of fatty acids and insulin signaling | ABCA1, ABCG1, CYP7A1, ABCB11, ATP8B1, CPT1A, CROT, HADHB, AMPK, SIRT6, IRS2 | [204-215] |

| | | | |
|---|---|---|---|
| **miR-34a** | Reduces SIRT1 expression and activity in many ways during obesity; regulates RXR$\alpha$ through binding to its 3′-UTR; inhibits beige and brown fat formation in obesity; mediates the hepatic response to metabolic stress associated with lipid overload; inhibits ABCA1/ABCG1 expression in macrophages regulating cholesterol efflux and reverse cholesterol transport (RCT); regulates lipid metabolism by targeting SIRT1; regulates intramuscular fat deposition in porcine adipocytes by targeting ACSL4; regulates the development and progression of NAFLD; promotes fat deposition in adipocytes and myoblasts by targeting LEF1 | NAMPT, RXR$\alpha$, FGF21, SIRT1, HNF4$\alpha$, ABCA1, ABCG1, ACSL4, LEF1 | [216-224] |
| **miR-106b** | Decrease ABCA1 expression and impair cellular cholesterol efflux in neuronal cells | ABCA1 | [225] |
| **miR-122** | Regulates lipid metabolism, reduces CYP7A1 mRNA stability to inhibit bile acid synthesis; upregulates the expression of the lipogenic genes; inhibits lipid droplet formation and hepatic triglyceride accumulation via Yin Yang 1; plays important roles in goose fatty liver; promotes hepatic lipogenesis; inhibition alleviates lipid accumulation and inflammation in NAFLD cell model | FAS, ACC2, SCD1, CYP7A1, SREBP-1c, DGAT2, ACC1, YY1, ALDOB, PKM2, SIRT1, TLR4/MyD88/NF-κBp65 | [226-232] |
| **miR-128-1** | Controls circulating lipoprotein metabolism; involved in lipogenesis | LDLR, ABCA1, FAS, SIRT1 | [233] |
| **miR-148a** | Regulates LDLR and ABCA1 expression to control circulating lipoprotein levels; exerts crucial roles on lipid metabolism and hepatocarcinogenesis; regulates low-density lipoprotein metabolism by repressing the (pro)renin receptor | LDLR, ABCA1, Hmgcr, Pgc1$\alpha$, Sirt7, Ybx1, (P)RR | [233-236] |
| **miR-223** | represses selective high-density lipoprotein cholesterol uptake through posttranscriptional inhibition; coordinates cholesterol homeostasis; inhibits lipid deposition and inflammation; targets the lipid metabolic gene DAGLA; attenuates lipid accumulation and liver fibrosis | SR-BI, HMGCS1, MSMO1, TLR4, DAGLA, E2F1 | [237-239] |
| **miR-224** | Regulates fatty acid metabolism; affects mammary epithelial cell apoptosis and triglyceride production; miR-224 and miR-520d control LDLR cell surface expression and LDL homeostasis; promote TG synthesis and lipid droplet formation | ACSL4, ACADM, ALDH2, PCSK9, IDOL, HMGCR, FABP4 | [69,240,241] |
| **miR-302a** | Involves hepatic cholesterol and fatty acid | ABCA1, ELOVL6 | [242,243] |

| | | | |
|---|---|---|---|
| | lipids; regulates cholesterol efflux | | |
| **miR-370** | Mitochondrial oxidation of fatty acids Controls the expression of miR-122 and Cpt1$\alpha$ and affects lipid metabolism | Cpt1$\alpha$ | [228] |
| **miR-378/378*** | Involves fatty acid and triglyceride biosynthesis; inhibits insulin signaling through targeting p110$\alpha$; prevents and treats obesity in mice by activating the pyruvate-PEP futile cycle in the muscle and enhancing lipolysis in adipose tissues; modulates bile acid and cholesterol metabolism; promotes the pathogenesis of nonalcoholic fatty liver disease | CRAT, Med13, p110$\alpha$, Nrf1, MAFG | [244-249] |
| **miR-483** | Affects cholesterol homeostasis by targeting PCSK9; post-natal miR-483 overexpression induces growth restriction and excessive adiposity | PCSK9 | [250,251] |
| **miR-520d** | MiR-224 and miR-520d control LDLR cell surface expression and LDL homeostasis | PCSK9, IDOL, HMGCR | [252] |
| **miR-613** | Regulates cholesterol homeostasis | LXR$\alpha$, ABCA1 | [253-255] |
| **miR-758** | Regulates cholesterol homeostasis; decreases lipid accumulation in foam cells by regulating CD36-mediated cholesterol uptake | ABCA1, CD36 | [256-258] |

**Supplemental Table S5.** LncRNAs are involved in lipid metabolism

| **LncRNA** | **Function** | **Targets** | Reference |
|---|---|---|---|
| **ApoA1-AS** | Recruiting SUZ12 to the APO gene cluster and promoting apolipoprotein expression | ApoA1, ApoA4, ApoC3 | [259] |
| **APOA4-AS** | Interacting with HuR and stabilizing APOA4 mRNA | APOA4 | [260] |
| **AT102202** | Promote cholesterol metabolism by decreasing the expression of HMGCR | HMGCR | [261] |
| **Blnc1** | Endothelial differentiation-related factor 1 can interact with Blnc1, constituting a ribonucleoprotein transcriptional complex with LXR | LXR | [262] |
| **CDKN2B-AS1** | Promotes cholesterol efflux by inhibiting ADAM10 | ADAM10 | [263] |
| **CHROME** | Promotes cholesterol efflux and hepatic HDL biogenesis | miR-27b, miR-33a, miR-33b, miR-128 | [264] |
| **DYNLRB2-2** | Upregulates cholesterol efflux by ABCA1 expression | ABCA1, TLR2 | [265,266] |
| **ENST00000416361** | Associated with CAD-induced lipid metabolism | SREBP-1, SREBP-2 | [267] |
| **ENST00000602558.1** | Regulates ABCG1 expression through binding to p65 | ABCG1 | [268] |
| **GAS5** | | | [269] |

| | | | |
|---|---|---|---|
| **Gm16551** | Represses lipid biosynthesis | SREBP-1c | [270] |
| **H19** | Stabilisation of the SREBP-1c protein, promotion of hepatic lipogenesis | SREBP-1c, miR-130a | [271-273] |
| **HOXC-AS1** | Decreased expression in human atherosclerotic plaques, limits ox-LDL-induced cholesterol accumulation | HOXC6 | [274] |
| **LASER** | Enhances the expression of cholesterol metabolism genes | LSD1 | [275] |
| **linc-GALNTL6-4** | Regulates the commitment of adipocytes towards the control of APOC1 levels and appropriate lipid patterns | APOC1 | [276] |
| **lncARSR** | Promotes hepatic cholesterol biosynthesis | SREBP-2, HMGCR | [277] |
| **lnc-KDM5D-4** | Increases lipid droplet formation in hepatocytes | PLIN2 | [278] |
| **lncSHGL** | Recruits hnRNPA1 to Suppress Hepatic Gluconeogenesis and Lipogenesis | hnRNPA1 | [279] |
| **LeXis** | LXR activation increases the level of LeXis to reduce serum and hepatic cholesterol levels | RALY | [280] |
| **LIPTER** | Preserves cardiac lipid metabolism | MYH10 | [281] |
| **lnc-HC** | Forms a complex with hnRNPA2B1 and inhibits ABCA1 expression, suppresses PPARγ expression through increasing the level of miR-130b-3p, leading to the triglyceride decrease | hnRNPA2B1, miR-130b-3p | [282,283] |
| **lncHLEF** | promotes hepatic lipid synthesis, enhances intramuscular fat deposition | miR-2188-3p/GATA6, ACLY | [284] |
| **lncHR1** | Inhibits SREBP 1c levels through the phosphorylation of the PDK1/AKT/FoxO1 axis | SREBP-1c | [285] |
| **lncLSTR** | Suppresses APOC2 expression | APOC2 | [286] |
| **LncNONMMUG027912** | Reduce lipid synthesis, promote lipid transport | SREBP-1c | [287] |
| **lncRHPL** | Regulates Hepatic VLDL Secretion by Modulating hnRNPU/ BMAL1/MTTP Axis | hnRNPU | [288] |
| **LOC286367** | Reduces the expression of ABCA1, leading to intracellular lipid accumulation | ABCA1 | [289] |
| **MALAT1** | Promotes hepatic steatosis and insulin resistance by increasing nuclear SREBP-1c protein stability, regulates cholesterol accumulation in ox-LDL-induced macrophages via the miRNA-17-5p/ABCA1 axis | SREBP-1c, miRNA-17-5p/ABCA1 | [290,291] |
| **MEG3** | Helps to alleviate lipid over-deposition, causes cholestasis by serving as a guide RNA scaffold to recruit PTBP1 to destabilize Shp mRNA | miR-21, PTBP1 | [292,293] |
| **MeXis** | Specific regulator of LXR-induced ABCA1 | DDX17 | [294] |

| | | | |
|---|---|---|---|
| | expression—amplifies Abca1 expression, enhancing macrophage cholesterol efflux | | |
| **MSC-AS** | Interacts with miR-33b-5p to up-regulate glycerol-3-phosphate acyltransferase, mitochondrial (GPAM), and promote triglyceride synthesis | miR-33b-5p | [295] |
| **NEAT1** | Influences TG and modulates ATGL expression, promotes the expression of ACC and FAS in hepatocytes, NEAT1-induced paraspeckles inhibit CD36 expression, promotes hepatic lipid accumulation via regulating miR-146a-5p/ROCK1, represses inflammation response and lipid uptake via modulating miR-342-3p, exacerbates nonalcoholic fatty liver through interrupting AMPK/SREBP-1 signaling | miR-124-3p, rapamycin (mTOR)/S6 K1, CD36, miR-146a-5p, miR-140, miR-342-3p, miR-372–3p | [296-302] |
| **NFIA-AS1** | Increases the circulation of HDL-C, reduces levels of LDL-C, and VLDL-C | miR-382-5p | [303] |
| **RP1-13D10.2** | Facilitates the expression of low-density lipoprotein receptors, thus increasing the uptake of plasma LDL-C | LDLR | [304] |
| **RP11-728F11.4** | Binding to the RNA recognition domain of EWSR1, increasing FXYD6 transcription and promoting CD36 expression | EWSR1 | [305] |
| **SPRY4-IT1** | Binds lipin 2 directly to downregulate the production of triglycerides | LPIN2 | [306] |
| **SRA** | Represses ATGL expression by inhibiting the transcriptional activity of forkhead protein O1 | FoxO1 | [307] |
| **TUG1** | Negatively regulates miR-92R to induce the expression of FXR1, affectes proliferation, apoptosis, and inflammatory cytokine expression via miR-133a/FGF1 regulatory axis | FXR1, miR-133a | [308,309] |
| **Uc.372** | Drives hepatic lipid accumulation by suppressing miR-195/miR4668 maturation | pri-miR-195/pri-miR-4668 | [310] |

**Supplemental Table S6.** CircRNAs are involved in lipid metabolism

| **circRNA** | **Function** | **Targets** | Reference |
|---|---|---|---|
| **CircACC1** | Assembles and stabilizes the AMPK complex and maintains basal activity, functions as an economic regulator under metabolic stress to promote β-oxidation and glycolysis | AMPK | [311] |
| **CircFUT10** | Plays a role in adipocyte proliferation and inhibits adipocyte differentiation via sponging let-7 | let-7c/let-e | [177] |
| **circH19** | Promotes the hADSCs differentiation and lipid accumulation via regulation of PTBP1 and SREBP1 | PTBP1 | [178] |

| | | | |
|---|---|---|---|
| **CircHIPK3** | Contributes to hyperglycemia and insulin resistance by sponging miR-192-5p and up-regulating FOXO1 | miR-192-5p | [312] |
| **circRNA_0000660** | Regulates lipid metabolism through the circRNA_0000660-miR_693-Igfbp1 regulatory pathway | miR_693 | [313] |
| **CircRNA_0046367** | Prevents hepatoxicity of lipid peroxidation, participates in tacrolimus-induced dysregulation of hepatic triglyceride homeostasis | miR-34a, miR-33a | [314,315] |
| **circRNA_0046366** | Inhibits hepatocellular steatosis by normalization of PPAR signaling | miR-34a | [316] |
| **CircSAMD4A** | Controls adipogenesis in obesity through the miR-138-5p/EZH2 axis | miR-138-5p | [170] |
| **circScd1** | Promotes Fatty Liver Disease via the Janus Kinase 2/Signal Transducer and Activator of Transcription 5 Pathway | JAK2/STAT5 | [317] |

**Supplemental Table S7.** The proposed roles of ncRNAs as diagnostic and prognostic markers for obesity and dyslipidemia/lipid profile abnormalities

| ncRNA | Function | models | Reference |
|---|---|---|---|
| **miR-33a, miR-122** | Both miR-33a and miR-122 are statistically significant links to BMI, waist circumference, weight, and height, as well as total cholesterol and triglycerides | obesity | [318] |
| **miR-155** | Reduces insulin resistance and glucose intolerance | high-fat diet (HFD) | [319] |
| **miR-34a** | Exhibits resistance to HFD obesity-encouraged glucose intolerance and protects against insulin resistance | HFD obesity | [320] |
| **miR-143-3p** | Reduce insulin resistance induced by obesity by controlling the target gene insulin-like growth factor-2 receptor (Igf2r) | obesity | [321] |
| **miR-21** | Promotes the advancement of NAFLD | NAFLD | [322] |
| **miR-122** | Ameliorate the development of NAFLD | NAFLD | [322] |
| **miR-223** | Ameliorate the development of NAFLD; EV-derived miR-223 inhibited the expression of genes involved in hepatic inflammation and fibrosis, thus playing a beneficial role in mitigating the progression of NAFLD | NAFLD | [322,323] |
| **H19** | protected animals against diet-induced obesity, and increased insulin sensitivity and mitochondrial synthesis | diet-induced obesity | [324] |
| **lncRNA-p19461, lncRNA-p21015, lncRNA-** | There was a significant correlation between circulating levels of these three lncRNAs and BMI, waist circumference, and waist-to-hip ratio; the circulating level of lncRNA-p19461 was negatively associated with insulin | obese patients | [325] |

| | | | |
|---|---|---|---|
| **p5549** | resistance and upregulated by diet-induced weight loss | | |
| **MIST** | The expression of MIST is inversely correlated with obesity and insulin resistance. | diet-induced obesity mice | [326] |
| **ROIT** | Improves insulin secretion and glucose homeostasis | obese mice, obese patients with type 2 diabetes | [327] |
| **SHNG9** | Prevent endothelial dysfunction in obese patients by suppressing inflammation and apoptosis | obese persons with endothelial dysfunction | [328] |
| **lnc-leptin** | Its dysregulation is significantly associated with obesity | ob/ob mice | [329] |
| **linc-ADAL** | The lncRNA linc-ADAL was up-regulated in adipose depots of obese individuals, and markedly induced during in vitro human adipocyte differentiation | obese individuals | [330] |
| **PVT1** | The obese mice had higher PVT1 expression in adipose tissue than that of nonobese mice, enhancing lipid amassing | obese mice | [127] |
| **RP11-20G13.3** | It is positively correlated with waist circumference, waist-to-hip ratio, body mass index (BMI), low-density lipoprotein cholesterol, leptin, and fasting insulin levels. | obese children | [331] |
| **H19, TUG1, MIAT, CASC11** | Could be detected in serum samples as a potential diagnostic marker in patients with atherosclerosis | patients with atherosclerosis | [332-335] |
| **CHROME** | The lncRNA CHROME was found to be present at higher levels in plasma and atherosclerotic plaques. | Individuals with coronary artery disease | [264] |
| **LeXis** | Plasma LeXis, has been identified as a non-invasive diagnostic biomarker for NASH | NASH | [336] |
| **hsa_circ_0017650, hsa_circ_0136134, hsa-circRNA9227-1** | The induced expression of hsa_circ_0017650, hsa_circ_0136134, and hsa-circRNA9227-1 was reported in human pre-adipocytes-visceral (HPA-v), predicting their potential as diagnostic and therapeutic targets for visceral obesity | human preadipocytes from visceral fat tissue (HPA-v) | [337] |
| **circSAMD4A** | circSAMD4A is an important molecule for the development of obesity, it has the potential to be used as a therapeutic target for treating obesity. | obese patients | [170] |

## References


1. Kennell, J.A.; Gerin, I.; MacDougald, O.A.; Cadigan, K.M. The microRNA miR-8 is a conserved negative regulator of Wnt signaling. *Proc Natl Acad Sci U S A* **2008**, *105*, 15417-15422.

2. Tan, Y.; Gan, M.; Fan, Y.; Li, L.; Zhong, Z.; Li, X.; Bai, L.; Zhao, Y.; Niu, L.; Shang, Y., et al. miR-10b-5p regulates 3T3-L1 cells differentiation by targeting Apol6. *Gene* **2019**, *687*, 39-46.
3. Li, H.; Li, T.; Wang, S.; Wei, J.; Fan, J.; Li, J.; Han, Q.; Liao, L.; Shao, C.; Zhao, R.C. miR-17-5p and miR-106a are involved in the balance between osteogenic and adipogenic differentiation of adipose-derived mesenchymal stem cells. *Stem Cell Res* **2013**, *10*, 313-324.
4. Wang, Q.; Li, Y.C.; Wang, J.; Kong, J.; Qi, Y.; Quigg, R.J.; Li, X. miR-17-92 cluster accelerates adipocyte differentiation by negatively regulating tumor-suppressor Rb2/p130. *Proc Natl Acad Sci U S A* **2008**, *105*, 2889-2894.
5. Kim, Y.J.; Hwang, S.J.; Bae, Y.C.; Jung, J.S. MiR-21 regulates adipogenic differentiation through the modulation of TGF-beta signaling in mesenchymal stem cells derived from human adipose tissue. *Stem Cells* **2009**, *27*, 3093-3102.
6. Li, M.; Zhang, N.; Li, J.; Zhang, W.; Hei, W.; Ji, M.; Yang, Y.; Cao, G.; Guo, X.; Li, B. MiR-23b Promotes Porcine Preadipocyte Differentiation via SESN3 and ACSL4. *Cells* **2022**, *11*, 2339.
7. Zaragosi, L.E.; Wdziekonski, B.; Brigand, K.L.; Villageois, P.; Mari, B.; Waldmann, R.; Dani, C.; Barbry, P. Small RNA sequencing reveals miR-642a-3p as a novel adipocyte-specific microRNA and miR-30 as a key regulator of human adipogenesis. *Genome Biol* **2011**, *12*, R64.
8. Li, M.; Liu, Z.; Zhang, Z.; Liu, G.; Sun, S.; Sun, C. miR-103 promotes 3T3-L1 cell adipogenesis through AKT/mTOR signal pathway with its target being MEF2D. *Biol Chem* **2015**, *396*, 235-244.
9. Zhai, B.; Li, H.; Li, S.; Gu, J.; Zhang, H.; Zhang, Y.; Li, H.; Tian, Y.; Li, G.; Wang, Y. Transcriptome analysis reveals FABP5 as a key player in the development of chicken abdominal fat, regulated by miR-122-5p targeting. *BMC Genomics* **2023**, *24*, 386.
10. Liu, H.; Wen, J.; Tian, X.; Li, T.; Zhao, J.; Cheng, J.; Huang, L.; Zhao, Y.; Cao, Q.; Jiang, J. miR-125a-3p regulates the expression of FSTL1, a pro-inflammatory factor, during adipogenic differentiation, and inhibits adipogenesis in mice. *FASEB J* **2023**, *37*, e23146.
11. Liu, J.; Liang, Y.; Qiao, L.; Xia, D.; Pan, Y.; Liu, W. MiR-128-1-5p regulates differentiation of ovine stromal vascular fraction by targeting the KLF11 5'-UTR. *Domest Anim Endocrinol* **2022**, *80*, 106711.
12. Qin, C.; Wang, H.; Zhong, J.; Ran, H.; Peng, W. miR-129 Regulates Yak Intramuscular Preadipocyte Proliferation and Differentiation through the PI3K/AKT Pathway. *International Journal of Molecular Sciences* **2024**, *25*, 632.
13. Li, X.; Zhang, H.; Wang, Y.; Li, Y.; Wang, Y.; Zhu, J.; Lin, Y. Screening of key miRNAs related with the differentiation of subcutaneous adipocytes and the validation of miR-133a-3p functional significance in goats. *Anim Biosci* **2023**, *36*, 144-155.
14. Yan, Y.; Yuan, J.; Luo, X.; Yu, X.; Lu, J.; Hou, W.; He, X.; Zhang, L.; Cao, J.; Wang, H. microRNA-140 Regulates PDGFRalpha and Is Involved in Adipocyte Differentiation. *Front Mol Biosci* **2022**, *9*, 907148.
15. Esau, C.; Kang, X.; Peralta, E.; Hanson, E.; Marcusson, E.G.; Ravichandran, L.V.; Sun, Y.; Koo, S.; Perera, R.J.; Jain, R., et al. MicroRNA-143 regulates adipocyte differentiation. *J Biol Chem* **2004**, *279*, 52361-52365.

16. Chen, L.; Hou, J.; Ye, L.; Chen, Y.; Cui, J.; Tian, W.; Li, C.; Liu, L. MicroRNA-143 regulates adipogenesis by modulating the MAP2K5-ERK5 signaling. *Sci Rep* **2014**, *4*, 3819.
17. Wang, Y.; Zhang, J.; Chu, X.; Wang, M.; Xin, Y.; Liu, S. MiR-146a-5p, targeting ErbB4, promotes 3T3-L1 preadipocyte differentiation through the ERK1/2/PPAR-$\gamma$ signaling pathway. *Lipids Health Dis* **2022**, *21*, 54.
18. Ahn, J.; Lee, H.; Jung, C.H.; Jeon, T.I.; Ha, T.Y. MicroRNA-146b promotes adipogenesis by suppressing the SIRT1-FOXO1 cascade. *EMBO Mol Med* **2013**, *5*, 1602-1612.
19. Chen, C.; Xiang, H.; Peng, Y.L.; Peng, J.; Jiang, S.W. Mature miR-183, negatively regulated by transcription factor GATA3, promotes 3T3-L1 adipogenesis through inhibition of the canonical Wnt/beta-catenin signaling pathway by targeting LRP6. *Cell Signal* **2014**, *26*, 1155-1165.
20. Yang, Y.; Zhang, W.; Li, H.; Xiang, H.; Zhang, C.; Du, Z.; Huang, L.; Zhu, J. MiR-196a Promotes Lipid Deposition in Goat Intramuscular Preadipocytes by Targeting MAP3K1 and Activating PI3K-Akt Pathway. *Cells* **2024**, *13*, 1459.
21. He, H.; Chen, K.; Wang, F.; Zhao, L.; Wan, X.; Wang, L.; Mo, Z. miR-204-5p promotes the adipogenic differentiation of human adipose-derived mesenchymal stem cells by modulating DVL3 expression and suppressing Wnt/beta-catenin signaling. *Int J Mol Med* **2015**, *35*, 1587-1595.
22. Huang, J.; Zhao, L.; Xing, L.; Chen, D. MicroRNA-204 regulates Runx2 protein expression and mesenchymal progenitor cell differentiation. *Stem Cells* **2010**, *28*, 357-364.
23. Qin, L.; Chen, Y.; Niu, Y.; Chen, W.; Wang, Q.; Xiao, S.; Li, A.; Xie, Y.; Li, J.; Zhao, X., et al. A deep investigation into the adipogenesis mechanism: profile of microRNAs regulating adipogenesis by modulating the canonical Wnt/beta-catenin signaling pathway. *BMC Genomics* **2010**, *11*, 320.
24. Wang, L.; Hu, X.; Wang, S.; Yuan, C.; Wang, Z.; Chang, G.; Chen, G. MicroRNA analysis reveals the role of miR-214 in duck adipocyte differentiation. *Anim Biosci* **2022**, *35*, 1327-1339.
25. Kim, J.Y.; Shin, K.K.; Lee, A.L.; Kim, Y.S.; Park, H.J.; Park, Y.K.; Bae, Y.C.; Jung, J.S. MicroRNA-302 induces proliferation and inhibits oxidant-induced cell death in human adipose tissue-derived mesenchymal stem cells. *Cell Death Dis* **2014**, *5*, e1385.
26. Lei, Z.; Wei, D.; Ma, Y.; Tang, L.; Wang, S.; Wang, P.; Pan, C.; Hu, C.; Wang, X.; Ma, Y. miR-302b promotes bovine preadipocyte differentiation and inhibits proliferation by targeting CDK2. *Anim Biotechnol* **2023**, *34*, 1447-1454.
27. Yang, Z.; Ma, X.; Zhang, D.; Li, B.; Gao, N.; Li, X.; Mei, C.; Zan, L. Bta-miR-330 promotes bovine intramuscular pre-adipocytes adipogenesis via targeting SESN3 to activate the Akt-mTOR signaling pathway. *Int J Biol Macromol* **2024**, *275*, 133650.
28. Zhu, L.; Chen, L.; Shi, C.M.; Xu, G.F.; Xu, L.L.; Zhu, L.L.; Guo, X.R.; Ni, Y.; Cui, Y.; Ji, C. MiR-335, an adipogenesis-related microRNA, is involved in adipose tissue inflammation. *Cell Biochem Biophys* **2014**, *68*, 283-290.

29. Ling, H.Y.; Wen, G.B.; Feng, S.D.; Tuo, Q.H.; Ou, H.S.; Yao, C.H.; Zhu, B.Y.; Gao, Z.P.; Zhang, L.; Liao, D.F. MicroRNA-375 promotes 3T3-L1 adipocyte differentiation through modulation of extracellular signal-regulated kinase signalling. *Clin Exp Pharmacol Physiol* **2011**, *38*, 239-246.
30. Huang, N.; Wang, J.; Xie, W.; Lyu, Q.; Wu, J.; He, J.; Qiu, W.; Xu, N.; Zhang, Y. MiR-378a-3p enhances adipogenesis by targeting mitogen-activated protein kinase 1. *Biochem Biophys Res Commun* **2015**, *457*, 37-42.
31. Giuliani, A.; Sabbatinelli, J.; Amatori, S.; Graciotti, L.; Silvestrini, A.; Matacchione, G.; Ramini, D.; Mensà, E.; Prattichizzo, F.; Babini, L., et al. MiR-422a promotes adipogenesis via MeCP2 downregulation in human bone marrow mesenchymal stem cells. *Cellular and Molecular Life Sciences* **2023**, *80*, 75.
32. Wu, W.; Liu, K.; You, Z.; Zhang, J. MiR-196b-3p and miR-450b-3p are key regulators of adipogenesis in porcine intramuscular and subcutaneous adipocytes. *BMC Genomics* **2023**, *24*, 360.
33. Kinoshita, M.; Ono, K.; Horie, T.; Nagao, K.; Nishi, H.; Kuwabara, Y.; Takanabe-Mori, R.; Hasegawa, K.; Kita, T.; Kimura, T. Regulation of adipocyte differentiation by activation of serotonin (5-HT) receptors 5-HT2AR and 5-HT2CR and involvement of microRNA-448-mediated repression of KLF5. *Mol Endocrinol* **2010**, *24*, 1978-1987.
34. Martinelli, R.; Nardelli, C.; Pilone, V.; Buonomo, T.; Liguori, R.; Castano, I.; Buono, P.; Masone, S.; Persico, G.; Forestieri, P., et al. miR-519d overexpression is associated with human obesity. *Obesity (Silver Spring)* **2010**, *18*, 2170-2176.
35. Sun, J.; Wang, Y.; Li, Y.; Zhao, G. Downregulation of PPARgamma by miR-548d-5p suppresses the adipogenic differentiation of human bone marrow mesenchymal stem cells and enhances their osteogenic potential. *J Transl Med* **2014**, *12*, 168.
36. Li, Y.; Li, J.; Yu, H.; Liu, Y.; Song, H.; Tian, X.; Liu, D.; Yan, C.; Han, Y. HOXA5-miR-574-5p axis promotes adipogenesis and alleviates insulin resistance. *Molecular Therapy Nucleic Acids* **2022**, *27*, 200-210.
37. Tan, X.; Zhu, T.; Zhang, L.; Fu, L.; Hu, Y.; Li, H.; Li, C.; Zhang, J.; Liang, B.; Liu, J. miR-669a-5p promotes adipogenic differentiation and induces browning in preadipocytes. *Adipocyte* **2022**, *11*, 120-132.
38. Sun, T.; Fu, M.; Bookout, A.L.; Kliewer, S.A.; Mangelsdorf, D.J. MicroRNA let-7 regulates 3T3-L1 adipogenesis. *Mol Endocrinol* **2009**, *23*, 925-931.
39. Xu, P.; Vernooy, S.Y.; Guo, M.; Hay, B.A. The Drosophila microRNA Mir-14 suppresses cell death and is required for normal fat metabolism. *Curr Biol* **2003**, *13*, 790-795.
40. Sun, G.; Li, F.; Ma, X.; Sun, J.; Jiang, R.; Tian, Y.; Han, R.; Li, G.; Wang, Y.; Li, Z., et al. gga-miRNA-18b-3p Inhibits Intramuscular Adipocytes Differentiation in Chicken by Targeting the ACOT13 Gene. *Cells* **2019**, *8*, 556.
41. Juiz-Valiña, P.; Varela-Rodríguez, B.M.; Outeiriño-Blanco, E.; García-Brao, M.J.; Mena, E.; Cordido, F.; Sangiao-Alvarellos, S. MiR-19 Family Impairs Adipogenesis by the Downregulation of the PPARγ Transcriptional Network. *International Journal of Molecular Sciences* **2022**, *23*, 15792.
42. Li, C.P.; Li, H.J.; Nie, J.; Chen, X.; Zhou, X. Mutation of miR-21 targets endogenous lipoprotein receptor-related protein 6 and nonalcoholic fatty liver disease. *Am J Transl Res* **2017**, *9*, 715-721.

43. Huang, S.; Wang, S.; Bian, C.; Yang, Z.; Zhou, H.; Zeng, Y.; Li, H.; Han, Q.; Zhao, R.C. Upregulation of miR-22 promotes osteogenic differentiation and inhibits adipogenic differentiation of human adipose tissue-derived mesenchymal stem cells by repressing HDAC6 protein expression. *Stem Cells Dev* **2012**, *21*, 2531-2540.
44. Zhang, L.; Li, X.; Xu, Q.; Huang, X.; Li, Y.; Liu, W.; Wang, Y.; Lin, Y. [miR-23b-3p regulates the differentiation of goat intramuscular preadipocytes by targeting the PDE4B gene]. *Sheng Wu Gong Cheng Xue Bao* **2023**, *39*, 4887-4900.
45. Lin, Z.; Tang, Y.; Li, Z.; Li, J.; Yu, C.; Yang, C.; Liu, L.; Wang, Y.; Liu, Y. miR-24-3p Dominates the Proliferation and Differentiation of Chicken Intramuscular Preadipocytes by Blocking ANXA6 Expression. *Genes* **2022**, *13*, 635.
46. Ding, N.; Wang, W.; Teng, J.; Zeng, Y.; Zhang, Q.; Dong, L.; Tang, H. miR-26a-5p Regulates Adipocyte Differentiation via Directly Targeting ACSL3 in Adipocytes. *Adipocyte* **2023**, *12*, 1-10.
47. Kim, S.Y.; Kim, A.Y.; Lee, H.W.; Son, Y.H.; Lee, G.Y.; Lee, J.W.; Lee, Y.S.; Kim, J.B. miR-27a is a negative regulator of adipocyte differentiation via suppressing PPARgamma expression. *Biochem Biophys Res Commun* **2010**, *392*, 323-328.
48. Karbiener, M.; Fischer, C.; Nowitsch, S.; Opriessnig, P.; Papak, C.; Ailhaud, G.; Dani, C.; Amri, E.Z.; Scheideler, M. microRNA miR-27b impairs human adipocyte differentiation and targets PPARgamma. *Biochem Biophys Res Commun* **2009**, *390*, 247-251.
49. He, A.; Zhu, L.; Gupta, N.; Chang, Y.; Fang, F. Overexpression of micro ribonucleic acid 29, highly up-regulated in diabetic rats, leads to insulin resistance in 3T3-L1 adipocytes. *Mol Endocrinol* **2007**, *21*, 2785-2794.
50. Zhang, A.; Lu, L.; Yang, F.; Luo, T.; Yang, S.; Yang, P.; Li, X.; Deng, X.; Qiu, Y.; Chen, L., et al. Effects of miR-29c on proliferation and adipogenic differentiation of porcine bone marrow mesenchymal stromal cells. *Adipocyte* **2024**, *13*, 2365211.
51. Tang, Y.F.; Zhang, Y.; Li, X.Y.; Li, C.; Tian, W.; Liu, L. Expression of miR-31, miR-125b-5p, and miR-326 in the adipogenic differentiation process of adipose-derived stem cells. *OMICS* **2009**, *13*, 331-336.
52. Zhang, W.; Raza, S.H.A.; Li, B.; Sun, B.; Wang, S.; Pant, S.D.; Al-Abbas, N.S.; Shaer, N.A.; Zan, L. miR-33a Inhibits the Differentiation of Bovine Preadipocytes through the IRS2–Akt Pathway. *Genes* **2023**, *14*, 529.
53. Taniguchi, M.; Nakajima, I.; Chikuni, K.; Kojima, M.; Awata, T.; Mikawa, S. MicroRNA-33b downregulates the differentiation and development of porcine preadipocytes. *Mol Biol Rep* **2014**, *41*, 1081-1090.
54. Wei, X.; Zhao, X.; Shan, X.; Zhu, Y.; Wang, S.; Chen, H.; Li, H.; Ma, Y. MiR-107 Regulates Adipocyte Differentiation and Adipogenesis by Targeting Apolipoprotein C-2 (APOC2) in Bovine. *Genes* **2022**, *13*, 1467.
55. Zhu, S.; Zhang, B.; Zhu, T.; Wang, D.; Liu, C.; Liu, Y.; He, Y.; Liang, W.; Li, W.; Han, R., et al. miR-128-3p inhibits intramuscular adipocytes differentiation in chickens by downregulating FDPS. *BMC Genomics* **2023**, *24*, 540.

56. Lee, E.K.; Lee, M.J.; Abdelmohsen, K.; Kim, W.; Kim, M.M.; Srikantan, S.; Martindale, J.L.; Hutchison, E.R.; Kim, H.H.; Marasa, B.S., et al. miR-130 suppresses adipogenesis by inhibiting peroxisome proliferator-activated receptor gamma expression. *Mol Cell Biol* **2011**, *31*, 626-638.
57. Yu, Y.; Chen, Y.; Wang, L.; Cheng, J.; Du, M.; Pan, S. Rno-miR-130b Attenuates Lipid Accumulation Through Promoting Apoptosis and Inhibiting Differentiation in Rat Intramuscular Adipocytes. *Int J Mol Sci* **2025**, *26*.
58. Li, Y.; He, C.; Ran, L.; Wang, Y.; Xiong, Y.; Wang, Y.; Zhu, J.; Lin, Y. miR-130b duplex (miR-130b-3p/miR-130b-5p) negatively regulates goat intramuscular preadipocyte lipid droplets accumulation by inhibiting Krüppel-like factor 3 expression. *J Anim Sci* **2023**, *101*.
59. Chen, C.; Peng, Y.; Peng, Y.; Peng, J.; Jiang, S. miR-135a-5p inhibits 3T3-L1 adipogenesis through activation of canonical Wnt/beta-catenin signaling. *J Mol Endocrinol* **2014**, *52*, 311-320.
60. Liu, J.; Che, Y.; Cai, K.; Zhao, B.; Qiao, L.; Pan, Y.; Yang, K.; Liu, W. miR-136 Regulates the Proliferation and Adipogenic Differentiation of Adipose-Derived Stromal Vascular Fractions by Targeting HSD17B12. *International Journal of Molecular Sciences* **2023**, *24*, 14892.
61. Luo, M.; Wang, L.; Xiao, C.; Zhou, M.; Li, M.; Li, H. miR136 regulates proliferation and differentiation of small tail han sheep preadipocytes. *Adipocyte* **2023**, *12*, 2173966.
62. Shin, K.K.; Kim, Y.S.; Kim, J.Y.; Bae, Y.C.; Jung, J.S. miR-137 controls proliferation and differentiation of human adipose tissue stromal cells. *Cell Physiol Biochem* **2014**, *33*, 758-768.
63. Yang, Z.; Bian, C.; Zhou, H.; Huang, S.; Wang, S.; Liao, L.; Zhao, R.C. MicroRNA hsa-miR-138 inhibits adipogenic differentiation of human adipose tissue-derived mesenchymal stem cells through adenovirus EID-1. *Stem Cells Dev* **2011**, *20*, 259-267.
64. Mi, L.; Chen, Y.; Zheng, X.; Li, Y.; Zhang, Q.; Mo, D.; Yang, G. MicroRNA-139-5p Suppresses 3T3-L1 Preadipocyte Differentiation Through Notch and IRS1/PI3K/Akt Insulin Signaling Pathways. *J Cell Biochem* **2015**, *116*, 1195-1204.
65. Guo, Y.; Chen, Y.; Zhang, Y.; Zhang, Y.; Chen, L.; Mo, D. Up-regulated miR-145 expression inhibits porcine preadipocytes differentiation by targeting IRS1. *Int J Biol Sci* **2012**, *8*, 1408-1417.
66. Liu, Y.; Wei, Y.; Dou, Y.; Li, C.; Song, C.; Zhang, Z.; Qi, K.; Li, X.; Qiao, R.; Wang, K., et al. Effect of miR-149-5p on intramuscular fat deposition in pigs based on metabolomics and transcriptomics. *BMC Genomics* **2023**, *24*, 293.
67. Liu, S.; Yang, Y.; Wu, J. TNFalpha-induced up-regulation of miR-155 inhibits adipogenesis by down-regulating early adipogenic transcription factors. *Biochem Biophys Res Commun* **2011**, *414*, 618-624.
68. Li, F.; Li, D.; Zhang, M.; Sun, J.; Li, W.; Jiang, R.; Han, R.; Wang, Y.; Tian, Y.; Kang, X., et al. miRNA-223 targets the GPAM gene and regulates the differentiation of intramuscular adipocytes. *Gene* **2019**, *685*, 106-113.
69. Peng, Y.; Xiang, H.; Chen, C.; Zheng, R.; Chai, J.; Peng, J.; Jiang, S. MiR-224 impairs adipocyte early differentiation and regulates fatty acid metabolism. *Int J Biochem Cell Biol* **2013**, *45*, 1585-1593.

70. Ling, H.Y.; Ou, H.S.; Feng, S.D.; Zhang, X.Y.; Tuo, Q.H.; Chen, L.X.; Zhu, B.Y.; Gao, Z.P.; Tang, C.K.; Yin, W.D., et al. CHANGES IN microRNA (miR) profile and effects of miR-320 in insulin-resistant 3T3-L1 adipocytes. *Clin Exp Pharmacol Physiol* **2009**, *36*, e32-39.
71. Li, X.; He, S.; Wu, P.; Zhou, Y.; Long, K.; Wang, T. MiR-328-5p inhibits the adipogenic differentiation of hMSCs by targeting fatty acid synthase. *Folia Histochem Cytobiol* **2022**, *60*, 292-300.
72. Chen, L.; Cui, J.; Hou, J.; Long, J.; Li, C.; Liu, L. A novel negative regulator of adipogenesis: microRNA-363. *Stem Cells* **2014**, *32*, 510-520.
73. Chen, M.; Zhang, C.; Wu, Z.; Guo, S.; Lv, W.; Song, J.; Hao, B.; Bai, J.; Zhang, X.; Xu, H., et al. Bta-miR-365-3p-targeted FK506-binding protein 5 participates in the AMPK/mTOR signaling pathway in the regulation of preadipocyte differentiation in cattle. *Anim Biosci* **2024**, *37*, 1156-1167.
74. Du, Y.; Jieqiong, M.; Yong, W.; Jiangjiang, Z.; Yanyan, L.; Qingyong, M.; and Lin, Y. MiR-421 regulates goat intramuscular preadipocytes differentiation via targeting FGF13. *Anim Biotechnol* **2022**, *33*, 1333-1343.
75. Ma, X.; Zhang, D.; Yang, Z.; Sun, M.; Gao, N.; Mei, C.; Zan, L. bta-miR-484 Inhibits Bovine Intramuscular Adipogenesis by Regulating Mitotic Clonal Expansion via the MAP3K9/JNK/CCND1 Axis. *J Agric Food Chem* **2025**, *73*, 1062-1074.
76. Yang, M.; Gao, X.; Hu, C.; Wang, S.; Sheng, H.; Ma, Y. Bta-miR-484 Targets SFRP1 and Affects Preadipocytes Proliferation, Differentiation, and Apoptosis. *International Journal of Molecular Sciences* **2023**, *24*, 12710.
77. Kim, Y.J.; Hwang, S.H.; Lee, S.Y.; Shin, K.K.; Cho, H.H.; Bae, Y.C.; Jung, J.S. miR-486-5p induces replicative senescence of human adipose tissue-derived mesenchymal stem cells and its expression is controlled by high glucose. *Stem Cells Dev* **2012**, *21*, 1749-1760.
78. Chen, L.; Chen, Y.; Zhang, S.; Ye, L.; Cui, J.; Sun, Q.; Li, K.; Wu, H.; Liu, L. MiR-540 as a novel adipogenic inhibitor impairs adipogenesis via suppression of PPARgamma. *J Cell Biochem* **2015**, *116*, 969-976.
79. Tang, B.; Yu, J.; Tang, R.; He, X.; Liu, J.; Liu, L.; Song, Z.; Shi, Y.; Zeng, Z.; Zhan, Y., et al. MiR-4769-3p suppresses adipogenesis in systemic sclerosis by negatively regulating the USP18/VDAC2 pathway. *iScience* **2024**, *27*, 110483.
80. Elsheikh, M.; Sano, T.; Mizokami, A.; Nakatsu, Y.; Asano, T.; Kanematsu, T. miR-6402 targets Bmpr2 and negatively regulates mouse adipogenesis. *Adipocyte* **2025**, *14*, 2474114.
81. Yang, M.; Gao, X.; Ma, Y.; Wang, X.; Lei, Z.; Wang, S.; Hu, H.; Tang, L.; Ma, Y. Bta-miR-6517 promotes proliferation and inhibits differentiation of pre-adipocytes by targeting PFKL. *J Anim Physiol Anim Nutr (Berl)* **2022**, *106*, 1197-1207.
82. Hu, C.; Yang, M.; Feng, X.; Wang, S.; Ma, Y.; Ma, Y. miR-10167-3p targets TCF7L1 to inhibit bovine adipocyte differentiation and promote bovine adipocyte proliferation. *Genomics* **2024**, *116*, 110903.
83. Yuan, Y.; Cao, X.; Hu, J.; Li, J.; Shen, D.; You, L.; Cui, X.; Wang, X.; Zhou, Y.; Gao, Y., et al. The role and possible mechanism of lncRNA AC092159.2 in modulating adipocyte differentiation. *J Mol Endocrinol* **2019**, *62*, 137-148.

84. Fan, L.; Xu, H.; Li, D.; Li, H.; Lu, D. A novel long noncoding RNA, AC092834.1, regulates the adipogenic differentiation of human adipose-derived mesenchymal stem cells via the DKK1/Wnt/beta-catenin signaling pathway. *Biochem Biophys Res Commun* **2020**, *525*, 747-754.
85. Huang, R.; Shi, C.; Liu, G. Long noncoding RNA ACART knockdown decreases 3T3-L1 preadipocyte proliferation and differentiation. *Open Life Sci* **2023**, *18*, 20220552.
86. Xiao, T.; Liu, L.; Li, H.; Sun, Y.; Luo, H.; Li, T.; Wang, S.; Dalton, S.; Zhao, R.C.; Chen, R. Long Noncoding RNA ADINR Regulates Adipogenesis by Transcriptionally Activating C/EBPalpha. *Stem Cell Reports* **2015**, *5*, 856-865.
87. Ma, X.; Yang, X.; Zhang, D.; Zhang, W.; Wang, X.; Xie, K.; He, J.; Mei, C.; Zan, L. RNA-seq analysis reveals the critical role of the novel lncRNA BIANCR in intramuscular adipogenesis through the ERK1/2 signaling pathway. *J Anim Sci Biotechnol* **2023**, *14*, 21.
88. Mi, L.; Zhao, X.Y.; Li, S.; Yang, G.; Lin, J.D. Conserved function of the long noncoding RNA Blnc1 in brown adipocyte differentiation. *Mol Metab* **2017**, *6*, 101-110.
89. Zhao, X.Y.; Li, S.; Wang, G.X.; Yu, Q.; Lin, J.D. A long noncoding RNA transcriptional regulatory circuit drives thermogenic adipocyte differentiation. *Mol Cell* **2014**, *55*, 372-382.
90. Li, X.; Hou, Z.; Meng, S.; Jia, Q.; Xing, S.; Wang, Z.; Chen, M.; Xu, H.; Li, M.; Cai, H. LncRNA BlncAD1 Modulates Bovine Adipogenesis by Binding to MYH10, PI3K/Akt Signaling Pathway, and miR-27a-5p/CDK6 Axis. *J Agric Food Chem* **2024**, *72*, 11094-11110.
91. Liu, W.; Ma, C.; Yang, B.; Yin, C.; Zhang, B.; Xiao, Y. LncRNA Gm15290 sponges miR-27b to promote PPARgamma-induced fat deposition and contribute to body weight gain in mice. *Biochem Biophys Res Commun* **2017**, *493*, 1168-1175.
92. Divoux, A.; Karastergiou, K.; Xie, H.; Guo, W.; Perera, R.J.; Fried, S.K.; Smith, S.R. Identification of a novel lncRNA in gluteal adipose tissue and evidence for its positive effect on preadipocyte differentiation. *Obesity (Silver Spring)* **2014**, *22*, 1781-1785.
93. Zhu, X.X.; Yan, Y.W.; Chen, D.; Ai, C.Z.; Lu, X.; Xu, S.S.; Jiang, S.; Zhong, G.S.; Chen, D.B.; Jiang, Y.Z. Long non-coding RNA HoxA-AS3 interacts with EZH2 to regulate lineage commitment of mesenchymal stem cells. *Oncotarget* **2016**, *7*, 63561-63570.
94. Nuermaimaiti, N.; Liu, J.; Liang, X.; Jiao, Y.; Zhang, D.; Liu, L.; Meng, X.; Guan, Y. Effect of lncRNA HOXA11-AS1 on adipocyte differentiation in human adipose-derived stem cells. *Biochem Biophys Res Commun* **2018**, *495*, 1878-1884.
95. Wang, J.; Chen, M.Y.; Chen, J.F.; Ren, Q.L.; Zhang, J.Q.; Cao, H.; Xing, B.S.; Pan, C.Y. LncRNA IMFlnc1 promotes porcine intramuscular adipocyte adipogenesis by sponging miR-199a-5p to up-regulate CAV-1. *BMC Mol Cell Biol* **2020**, *21*, 77.
96. Zhang, M.; Li, F.; Sun, J.W.; Li, D.H.; Li, W.T.; Jiang, R.R.; Li, Z.J.; Liu, X.J.; Han, R.L.; Li, G.X., et al. LncRNA IMFNCR Promotes Intramuscular Adipocyte Differentiation by Sponging miR-128-3p and miR-27b-3p. *Front Genet* **2019**, *10*, 42.
97. Yu, Y.; Chen, Y.; Zhang, X.; Lu, X.; Hong, J.; Guo, X.; Zhou, D. Knockdown of lncRNA KCNQ1OT1 suppresses the adipogenic and osteogenic differentiation of tendon stem cell via downregulating miR-138 target genes PPARgamma and RUNX2. *Cell Cycle* **2018**, *17*, 2374-2385.

98. Zhu, R.; Guo, D.; Li, R.; Feng, Y.; Yang, X.; Huang, Q.; Zheng, Y.; Shi, D.; Huang, J. A long non-coding RNA lnc210 promotes adipogenic differentiation of buffalo intramuscular adipocytes. *Anim Biotechnol* **2023**, *34*, 2736-2744.
99. Yue, Y.; Ge, Z.; Guo, Z.; Wang, Y.; Yang, G.; Sun, S.; Li, X. Screening of lncRNA profiles during intramuscular adipogenic differentiation in longissimus dorsi and semitendinosus muscles in pigs. *Anim Biotechnol* **2023**, *34*, 4616-4626.
100. Ma, X.; He, Y.; Liu, C.; Zhu, T.; Li, D.; Li, W.; Sun, G.; Kang, X. Long Noncoding RNA 6302 Regulates Chicken Preadipocyte Differentiation by Targeting SLC22A16. *Genes (Basel)* **2024**, *15*.
101. Xu, H.; Yang, Y.; Fan, L.; Deng, L.; Fan, J.; Li, D.; Li, H.; Zhao, R.C. Lnc13728 facilitates human mesenchymal stem cell adipogenic differentiation via positive regulation of ZBED3 and downregulation of the WNT/beta-catenin pathway. *Stem Cell Res Ther* **2021**, *12*, 176.
102. Kang, Z.; Zhang, S.; Jiang, E.; Wang, X.; Wang, Z.; Chen, H.; Lan, X. circFLT1 and lncCCPG1 Sponges miR-93 to Regulate the Proliferation and Differentiation of Adipocytes by Promoting lncSLC30A9 Expression. *Mol Ther Nucleic Acids* **2020**, *22*, 484-499.
103. Li, R.; Zhu, R.; Yang, X.; Feng, Y.; He, Q.; Wang, H.; Liu, Q.; Shi, D.; Huang, J. The role of lncFABP4 in modulating adipogenic differentiation in buffalo intramuscular preadipocytes. *Anim Sci J* **2024**, *95*, e13951.
104. Feng, M.; Yi, X.; Zhang, Z.; Zhu, J.; Yu, H.; Ming, L.; Pang, W. LncIMF1 Promotes Adipogenesis of Porcine Intramuscular Preadipocyte by Sponging miR-187. *Biochem Genet* **2025**, 10.1007/s10528-025-11061-y.
105. Yi, X.; He, Z.; Tian, T.; Kou, Z.; Pang, W. LncIMF2 promotes adipogenesis in porcine intramuscular preadipocyte through sponging MiR-217. *Anim Biotechnol* **2023**, *34*, 268-279.
106. Yang, Y.; Wu, Y.; Ji, M.; Rong, X.; Zhang, Y.; Yang, S.; Lu, C.; Cai, C.; Gao, P.; Guo, X., et al. The long non-coding RNA lncMYOZ2 mediates an AHCY/MYOZ2 axis to promote adipogenic differentiation in porcine preadipocytes. *BMC Genomics* **2022**, *23*, 700.
107. Wang, Z.; Luo, Z.; Dai, Z.; Zhong, Y.; Liu, X.; Zuo, C. Long non-coding RNA lnc-OAD is required for adipocyte differentiation in 3T3-L1 preadipocytes. *Biochem Biophys Res Commun* **2019**, *511*, 753-758.
108. Cai, R.; Tang, G.; Zhang, Q.; Yong, W.; Zhang, W.; Xiao, J.; Wei, C.; He, C.; Yang, G.; Pang, W. A Novel lnc-RNA, Named lnc-ORA, Is Identified by RNA-Seq Analysis, and Its Knockdown Inhibits Adipogenesis by Regulating the PI3K/AKT/mTOR Signaling Pathway. *Cells* **2019**, *8*.
109. Wang, Z.; Chai, J.; Wang, Y.; Gu, Y.; Long, K.; Li, M.; Jin, L. LncPLAAT3-AS Regulates PLAAT3-Mediated Adipocyte Differentiation and Lipogenesis in Pigs through miR-503-5p. *Genes (Basel)* **2023**, *14*.
110. Chen, Y.; Li, K.; Zhang, X.; Chen, J.; Li, M.; Liu, L. The novel long noncoding RNA lncRNA-Adi regulates adipogenesis. *Stem Cells Transl Med* **2020**, *9*, 1053-1067.

111. Zhu, R.; Feng, X.; Wei, Y.; Guo, D.; Li, J.; Liu, Q.; Jiang, J.; Shi, D.; Huang, J. lncSAMM50 Enhances Adipogenic Differentiation of Buffalo Adipocytes With No Effect on Its Host Gene. *Front Genet* **2021**, *12*, 626158.
112. Huang, X.; Liu, X.; Lin, J. Methylation of lncSHGL promotes adipocyte differentiation by regulating miR-149/Mospd3 axis. *Cell Cycle* **2023**, *22*, 2361-2380.
113. Shao, Y.; Xiong, M.; Liu, J.; Gu, Z.; Wu, Z.; Cao, L. LOC646762 Is Involved in Adipogenic Differentiation of Bone Marrow-Derived Mesenchymal Stem Cells. *ACS Omega* **2024**, *9*, 8464-8470.
114. Yang, Y.; Fan, J.; Xu, H.; Fan, L.; Deng, L.; Li, J.; Li, D.; Li, H.; Zhang, F.; Zhao, R.C. Long noncoding RNA LYPLAL1-AS1 regulates adipogenic differentiation of human mesenchymal stem cells by targeting desmoplakin and inhibiting the Wnt/beta-catenin pathway. *Cell Death Discov* **2021**, *7*, 105.
115. Li, D.; Chen, Y.; Zhu, X.; Yang, Y.; Li, H.; Zhao, R.C. A novel human specific lncRNA MEK6-AS1 regulates adipogenesis and fatty acid biosynthesis by stabilizing MEK6 mRNA. *J Biomed Sci* **2025**, *32*, 6.
116. Huang, Y.; Jin, C.; Zheng, Y.; Li, X.; Zhang, S.; Zhang, Y.; Jia, L.; Li, W. Knockdown of lncRNA MIR31HG inhibits adipocyte differentiation of human adipose-derived stem cells via histone modification of FABP4. *Sci Rep* **2017**, *7*, 8080.
117. Zhang, L.; Ma, J.; Pan, X.; Zhang, M.; Huang, W.; Liu, Y.; Yang, H.; Cheng, Z.; Zhang, G.; Qie, M., et al. LncRNA MIR99AHG enhances adipocyte differentiation by targeting miR-29b-3p to upregulate PPARγ. *Molecular and Cellular Endocrinology* **2022**, *550*, 111648.
118. Thunen, A.; La Placa, D.; Zhang, Z.; Shively, J.E. Role of lncRNA LIPE-AS1 in adipogenesis. *Adipocyte* **2022**, *11*, 11-27.
119. Tang, T.; Jiang, G.; Shao, J.; Wang, M.; Zhang, X.; Xia, S.; Sun, W.; Jia, X.; Wang, J.; Lai, S. lncRNA MSTRG4710 Promotes the Proliferation and Differentiation of Preadipocytes through miR-29b-3p/IGF1 Axis. *Int J Mol Sci* **2023**, *24*.
120. Lin, W.; Chen, L.; Meng, W.; Yang, K.; Wei, S.; Wei, W.; Chen, J.; Zhang, L. C/EBPalpha promotes porcine pre-adipocyte proliferation and differentiation via mediating MSTRG.12568.2/FOXO3 trans-activation for STYX. *Biochim Biophys Acta Mol Cell Biol Lipids* **2022**, *1867*, 159206.
121. Huang, J.; Zheng, Q.; Wang, S.; Wei, X.; Li, F.; Ma, Y. High-Throughput RNA Sequencing Reveals NDUFC2-AS lncRNA Promotes Adipogenic Differentiation in Chinese Buffalo (Bubalus bubalis L). *Genes (Basel)* **2019**, *10*.
122. Cooper, D.R.; Carter, G.; Li, P.; Patel, R.; Watson, J.E.; Patel, N.A. Long Non-Coding RNA NEAT1 Associates with SRp40 to Temporally Regulate PPARgamma2 Splicing during Adipogenesis in 3T3-L1 Cells. *Genes (Basel)* **2014**, *5*, 1050-1063.
123. Gernapudi, R.; Wolfson, B.; Zhang, Y.; Yao, Y.; Yang, P.; Asahara, H.; Zhou, Q. MicroRNA 140 Promotes Expression of Long Noncoding RNA NEAT1 in Adipogenesis. *Mol Cell Biol* **2016**, *36*, 30-38.
124. Firmin, F.F.; Oger, F.; Gheeraert, C.; Dubois-Chevalier, J.; Vercoutter-Edouart, A.S.; Alzaid, F.; Mazuy, C.; Dehondt, H.; Alexandre, J.; Derudas, B., et al. The RBM14/CoAA-interacting, long intergenic non-coding RNA Paral1 regulates adipogenesis and coactivates the nuclear receptor PPARgamma. *Sci Rep* **2017**, *7*, 14087.

125. Zhu, E.; Zhang, J.; Li, Y.; Yuan, H.; Zhou, J.; Wang, B. Long noncoding RNA Plnc1 controls adipocyte differentiation by regulating peroxisome proliferator-activated receptor gamma. *FASEB J* **2019**, *33*, 2396-2408.
126. Pang, W.J.; Lin, L.G.; Xiong, Y.; Wei, N.; Wang, Y.; Shen, Q.W.; Yang, G.S. Knockdown of PU.1 AS lncRNA inhibits adipogenesis through enhancing PU.1 mRNA translation. *J Cell Biochem* **2013**, *114*, 2500-2512.
127. Zhang, L.; Zhang, D.; Qin, Z.Y.; Li, J.; Shen, Z.Y. The role and possible mechanism of long noncoding RNA PVT1 in modulating 3T3-L1 preadipocyte proliferation and differentiation. *IUBMB Life* **2020**, *72*, 1460-1467.
128. Lin, Y.; Zhang, Y.; Xu, L.; Long, W.; Shan, C.; Ding, H.; You, L.; Zhao, C.; Shi, Z. High expression of an unknown long noncoding RNA RP11-290L1.3 from GDM macrosomia and its effect on preadipocyte differentiation. *Endocr Connect* **2021**, *10*, 191-204.
129. Zhang, T.; Liu, H.; Mao, R.; Yang, H.; Zhang, Y.; Zhang, Y.; Guo, P.; Zhan, D.; Xiang, B.; Liu, Y. The lncRNA RP11-142A22.4 promotes adipogenesis by sponging miR-587 to modulate Wnt5beta expression. *Cell Death Dis* **2020**, *11*, 475.
130. Zhang, D.; Ma, X.; Li, H.; Li, X.; Wang, J.; Zan, L. SERPINE1AS2 regulates intramuscular adipogenesis by inhibiting PAI1 protein expression. *Int J Biol Macromol* **2024**, *275*, 133592.
131. Yi, F.; Yang, F.; Liu, X.; Chen, H.; Ji, T.; Jiang, L.; Wang, X.; Yang, Z.; Zhang, L.H.; Ding, X., et al. RNA-seq identified a super-long intergenic transcript functioning in adipogenesis. *RNA Biol* **2013**, *10*, 991-1001.
132. Yu, X.; Song, M.S.; Rong, P.Z.; Chen, X.J.; Shi, L.; Wang, C.H.; Pang, Q.J. LncRNA SNHG1 modulates adipogenic differentiation of BMSCs by promoting DNMT1 mediated Opg hypermethylation via interacting with PTBP1. *J Cell Mol Med* **2022**, *26*, 60-74.
133. Xu, B.; Gerin, I.; Miao, H.; Vu-Phan, D.; Johnson, C.N.; Xu, R.; Chen, X.W.; Cawthorn, W.P.; MacDougald, O.A.; Koenig, R.J. Multiple roles for the non-coding RNA SRA in regulation of adipogenesis and insulin sensitivity. *PLoS One* **2010**, *5*, e14199.
134. Liu, S.; Xu, R.; Gerin, I.; Cawthorn, W.P.; Macdougald, O.A.; Chen, X.W.; Saltiel, A.R.; Koenig, R.J.; Xu, B. SRA regulates adipogenesis by modulating p38/JNK phosphorylation and stimulating insulin receptor gene expression and downstream signaling. *PLoS One* **2014**, *9*, e95416.
135. Cai, R.; Sun, Y.; Qimuge, N.; Wang, G.; Wang, Y.; Chu, G.; Yu, T.; Yang, G.; Pang, W. Adiponectin AS lncRNA inhibits adipogenesis by transferring from nucleus to cytoplasm and attenuating Adiponectin mRNA translation. *Biochim Biophys Acta Mol Cell Biol Lipids* **2018**, *1863*, 420-432.
136. Li, M.; Sun, X.; Cai, H.; Sun, Y.; Plath, M.; Li, C.; Lan, X.; Lei, C.; Lin, F.; Bai, Y., et al. Long non-coding RNA ADNCR suppresses adipogenic differentiation by targeting miR-204. *Biochim Biophys Acta* **2016**, *1859*, 871-882.
137. Cai, H.; Li, M.; Jian, W.; Song, C.; Huang, Y.; Lan, X.; Lei, C.; Chen, H. A novel lncRNA BADLNCR1 inhibits bovine adipogenesis by repressing GLRX5 expression. *J Cell Mol Med* **2020**, *24*, 7175-7186.

138. Shen, L.; Han, J.; Wang, H.; Meng, Q.; Chen, L.; Liu, Y.; Feng, Y.; Wu, G. Cachexia-related long noncoding RNA, CAAlnc1, suppresses adipogenesis by blocking the binding of HuR to adipogenic transcription factor mRNAs. *Int J Cancer* **2019**, *145*, 1809-1821.
139. Chai, J.; Wang, N.; Chen, L.; Bai, J.; Zhang, J.; Zhang, G.; An, J.; Zhang, T.; Tong, X.; Wu, Y., et al. Identification of a Novel Long Non-Coding RNA G8110 That Modulates Porcine Adipogenic Differentiation and Inflammatory Responses. *Int J Mol Sci* **2023**, *24*.
140. Liu, H.; Li, H.; Jin, L.; Li, G.; Hu, S.; Ning, C.; Guo, J.; Shuai, S.; Li, X.; Li, M. Long Noncoding RNA GAS5 Suppresses 3T3-L1 Cells Adipogenesis Through miR-21a-5p/PTEN Signal Pathway. *DNA Cell Biol* **2018**, *37*, 767-777.
141. Li, M.; Xie, Z.; Wang, P.; Li, J.; Liu, W.; Tang, S.; Liu, Z.; Wu, X.; Wu, Y.; Shen, H. The long noncoding RNA GAS5 negatively regulates the adipogenic differentiation of MSCs by modulating the miR-18a/CTGF axis as a ceRNA. *Cell Death Dis* **2018**, *9*, 554.
142. You, L.; Zhou, Y.; Cui, X.; Wang, X.; Sun, Y.; Gao, Y.; Wang, X.; Wen, J.; Xie, K.; Tang, R., et al. GM13133 is a negative regulator in mouse white adipocytes differentiation and drives the characteristics of brown adipocytes. *J Cell Physiol* **2018**, *233*, 313-324.
143. Huang, Y.; Zheng, Y.; Jin, C.; Li, X.; Jia, L.; Li, W. Long Non-coding RNA H19 Inhibits Adipocyte Differentiation of Bone Marrow Mesenchymal Stem Cells through Epigenetic Modulation of Histone Deacetylases. *Sci Rep* **2016**, *6*, 28897.
144. Li, D.; Liu, Y.; Gao, W.; Han, J.; Yuan, R.; Zhang, M.; Ge, Z. LncRNA HCG11 Inhibits Adipocyte Differentiation in Human Adipose-Derived Mesenchymal Stem Cells by Sponging miR-204-5p to Upregulate SIRT1. *Cell Transplant* **2020**, *29*, 963689720968090.
145. Jiang, Q.; Zhang, S.; Gao, X.; Hu, Y.; Zhang, Y.; Shen, Y.; Jiang, Y.; Huang, Y. Resveratrol Inhibits Proliferation and Differentiation of Porcine Preadipocytes by a Novel LincRNA-ROFM/miR-133b/AdipoQ Pathway. *Foods* **2022**, *11*.
146. Zhang, W.; Raza, S.H.A.; Li, B.; Yang, W.; Khan, R.; Aloufi, B.H.; Zhang, G.; Zuo, F.; Zan, L. LncBNIP3 Inhibits Bovine Intramuscular Preadipocyte Differentiation via the PI3K-Akt and PPAR Signaling Pathways. *J Agric Food Chem* **2024**, *72*, 24260-24271.
147. Xiao, F.; Tang, C.Y.; Tang, H.N.; Wu, H.X.; Hu, N.; Li, L.; Zhou, H.D. Long Non-coding RNA 332443 Inhibits Preadipocyte Differentiation by Targeting Runx1 and p38-MAPK and ERK1/2-MAPK Signaling Pathways. *Front Cell Dev Biol* **2021**, *9*, 663959.
148. Sun, Y.; Cai, R.; Wang, Y.; Zhao, R.; Qin, J.; Pang, W. A Newly Identified LncRNA LncIMF4 Controls Adipogenesis of Porcine Intramuscular Preadipocyte through Attenuating Autophagy to Inhibit Lipolysis. *Animals (Basel)* **2020**, *10*.
149. Ming, Y.; Liu, Z.P. Overexpression of lncRNA-NEF regulates the miR-155/PTEN axis to inhibit adipogenesis and promote osteogenesis. *Kaohsiung J Med Sci* **2021**, *37*, 930-939.
150. Chen, J.; Liu, Y.; Lu, S.; Yin, L.; Zong, C.; Cui, S.; Qin, D.; Yang, Y.; Guan, Q.; Li, X., et al. The role and possible mechanism of lncRNA U90926 in modulating 3T3-L1 preadipocyte differentiation. *Int J Obes (Lond)* **2017**, *41*, 299-308.
151. Huang, X.; Fu, C.; Liu, W.; Liang, Y.; Li, P.; Liu, Z.; Sheng, Q.; Liu, P. Chemerin-induced angiogenesis and adipogenesis in 3 T3-L1 preadipocytes is mediated by lncRNA Meg3 through regulating Dickkopf-3 by sponging miR-217. *Toxicol Appl Pharmacol* **2019**, *385*, 114815.

152. Li, Z.; Jin, C.; Chen, S.; Zheng, Y.; Huang, Y.; Jia, L.; Ge, W.; Zhou, Y. Long non-coding RNA MEG3 inhibits adipogenesis and promotes osteogenesis of human adipose-derived mesenchymal stem cells via miR-140-5p. *Mol Cell Biochem* **2017**, *433*, 51-60.
153. Li, M.; Gao, Q.; Tian, Z.; Lu, X.; Sun, Y.; Chen, Z.; Zhang, H.; Mao, Y.; Yang, Z. MIR221HG Is a Novel Long Noncoding RNA that Inhibits Bovine Adipocyte Differentiation. *Genes (Basel)* **2019**, *11*.
154. Zuo, C.; Pan, Y.; Leng, D.; Chen, X.; Dong, F.; Lin, Z.; Dai, Z.; Wang, Z. Transcriptome analysis of long non-coding RNAs reveals NR_015556 lncRNA is a novel regulator for adipocyte differentiation. *Biochem Biophys Res Commun* **2022**, *601*, 79-85.
155. Pan, Y.; Xie, Z.; Cen, S.; Li, M.; Liu, W.; Tang, S.; Ye, G.; Li, J.; Zheng, G.; Li, Z., et al. Long noncoding RNA repressor of adipogenesis negatively regulates the adipogenic differentiation of mesenchymal stem cells through the hnRNP A1-PTX3-ERK axis. *Clin Transl Med* **2020**, *10*, e227.
156. Shang, G.; Wang, Y.; Xu, Y.; Zhang, S.; Sun, X.; Guan, H.; Zhao, X.; Wang, Y.; Li, Y.; Zhao, G. Long non-coding RNA TCONS_00041960 enhances osteogenesis and inhibits adipogenesis of rat bone marrow mesenchymal stem cell by targeting miR-204-5p and miR-125a-3p. *J Cell Physiol* **2018**, *233*, 6041-6051.
157. Li, X.; Zhang, H.; Wang, Y.; Li, Y.; Wang, Y.; Zhu, J.; Lin, Y. Chi-Circ_0006511 Positively Regulates the Differentiation of Goat Intramuscular Adipocytes via Novel-miR-87/CD36 Axis. *International Journal of Molecular Sciences* **2022**, *23*, 12295.
158. Zhi, F.; Ding, Y.; Wang, R.; Yang, Y.; Luo, K.; Hua, F. Exosomal hsa_circ_0006859 is a potential biomarker for postmenopausal osteoporosis and enhances adipogenic versus osteogenic differentiation in human bone marrow mesenchymal stem cells by sponging miR-431-5p. *Stem Cell Res Ther* **2021**, *12*, 157.
159. Song, X.H.; He, N.; Xing, Y.T.; Jin, X.Q.; Li, Y.W.; Liu, S.S.; Gao, Z.Y.; Guo, C.; Wang, J.J.; Huang, Y.Y., et al. A Novel Age-Related Circular RNA Circ-ATXN2 Inhibits Proliferation, Promotes Cell Death and Adipogenesis in Rat Adipose Tissue-Derived Stromal Cells. *Front Genet* **2021**, *12*, 761926.
160. Zhang, S.; Jiang, E.; Kang, Z.; Bi, Y.; Liu, H.; Xu, H.; Wang, Z.; Lei, C.; Chen, H.; Lan, X. CircRNA Profiling Reveals an Abundant circBDP1 that Regulates Bovine Fat Development by Sponging miR-181b/miR-204 Targeting Sirt1/TRARG1. *Journal of Agricultural and Food Chemistry* **2022**, *70*, 14312-14328.
161. Chen, G.; Wang, Q.; Li, Z.; Yang, Q.; Liu, Y.; Du, Z.; Zhang, G.; Song, Y. Circular RNA CDR1as promotes adipogenic and suppresses osteogenic differentiation of BMSCs in steroid-induced osteonecrosis of the femoral head. *Bone* **2020**, *133*, 115258.
162. Tian, W.; Liu, Y.; Zhang, W.; Nie, R.; Ling, Y.; Zhang, B.; Zhang, H.; Wu, C. CircDOCK7 facilitates the proliferation and adipogenic differentiation of chicken abdominal preadipocytes through the gga-miR-301b-3p/ACSL1 axis. *J Anim Sci Biotechnol* **2023**, *14*, 91.
163. Feng, X.; Zhao, J.; Li, F.; Aloufi, B.H.; Alshammari, A.M.; Ma, Y. Weighted Gene Co-expression Network Analysis Revealed That CircMARK3 Is a Potential CircRNA Affects Fat Deposition in Buffalo. *Front Vet Sci* **2022**, *9*, 946447.

164. Chen, S.; Song, P.; Wang, Y.; Wang, Z.; Xue, J.; Jiang, Y.; Zhou, Y.; Zhao, J.; Tang, L. CircMAPK9 promotes adipogenesis through modulating hsa-miR-1322/FTO axis in obesity. *iScience* **2023**, *26*, 107756.
165. Huang, L.; He, S.; Wang, T.; Long, K.; Ma, B.; Wu, P.; Gong, Y.; Zhong, D.; Yang, Q.; Wu, J., et al. circNDUFA13 stimulates adipogenesis of bone marrow-derived mesenchymal stem cells via interaction with STAT3. *Sci Rep* **2024**, *14*, 19787.
166. Wang, L.; Liang, W.; Wang, S.; Wang, Z.; Bai, H.; Jiang, Y.; Bi, Y.; Chen, G.; Chang, G. Circular RNA expression profiling reveals that circ-PLXNA1 functions in duck adipocyte differentiation. *PLoS One* **2020**, *15*, e0236069.
167. Li, B.; He, Y.; Wu, W.; Tan, X.; Wang, Z.; Irwin, D.M.; Wang, Z.; Zhang, S. Circular RNA Profiling Identifies Novel circPPARA that Promotes Intramuscular Fat Deposition in Pigs. *J Agric Food Chem* **2022**, *70*, 4123-4137.
168. Wu, J.; Zhang, S.; Yue, B.; Zhang, S.; Jiang, E.; Chen, H.; Lan, X. CircRNA Profiling Reveals CircPPARγ Modulates Adipogenic Differentiation via Sponging miR-92a-3p. *Journal of Agricultural and Food Chemistry* **2022**, *70*, 6698-6708.
169. Shen, X.; Tang, J.; Huang, Y.; Lan, X.; Lei, C.; Chen, H. CircRNF111 Contributes to Adipocyte Differentiation by Elevating PPARgamma Expression via miR-27a-3p. *Epigenetics* **2023**, *18*, 2145058.
170. Liu, Y.; Liu, H.; Li, Y.; Mao, R.; Yang, H.; Zhang, Y.; Zhang, Y.; Guo, P.; Zhan, D.; Zhang, T. Circular RNA SAMD4A controls adipogenesis in obesity through the miR-138-5p/EZH2 axis. *Theranostics* **2020**, *10*, 4705-4719.
171. Liu, Y.; Dou, Y.; Qi, K.; Li, C.; Song, C.; Li, X.; Li, X.; Qiao, R.; Wang, K.; Han, X. CircSETBP1 Acts as a MiR-149-5p Sponge to Promote Intramuscular Fat Deposition by Regulating CRTCs. *Journal of Agricultural and Food Chemistry* **2022**, *70*, 12841-12851.
172. Liang, Y.; Zhao, B.; Shen, Y.; Peng, M.; Qiao, L.; Liu, J.; Pan, Y.; Yang, K.; Liu, W. Elucidating the Role of circTIAM1 in Guangling Large-Tailed Sheep Adipocyte Proliferation and Differentiation via the miR-485-3p/PLCB1 Pathway. *International Journal of Molecular Sciences* **2024**, *25*, 4588.
173. Arcinas, C.; Tan, W.; Fang, W.; Desai, T.P.; Teh, D.C.S.; Degirmenci, U.; Xu, D.; Foo, R.; Sun, L. Adipose circular RNAs exhibit dynamic regulation in obesity and functional role in adipogenesis. *Nature Metabolism* **2019**, *1*, 688-703.
174. Wang, J.M.; Lv, J.S.; Liu, K.H.; Li, Y.Y.; Zhu, J.J.; Xiong, Y.; Wang, Y.; Lin, Y.Q. Circ_0011446 Regulates Intramuscular Adipocyte Differentiation in Goats via the miR-27a-5p/FAM49B Axis. *Int J Mol Sci* **2025**, *26*.
175. Hu, C.; Feng, X.; Ma, Y.; Wei, D.; Zhang, L.; Wang, S.; Ma, Y. CircADAMTS16 Inhibits Differentiation and Promotes Proliferation of Bovine Adipocytes by Targeting miR-10167-3p. *Cells* **2023**, *12*, 1175.
176. Ma, Z.; Chen, Y.; Qiu, J.; Guo, R.; Cai, K.; Zheng, Y.; Zhang, Y.; Li, X.; Zan, L.; Li, A. CircBTBD7 inhibits adipogenesis via the miR-183/SMAD4 axis. *Int J Biol Macromol* **2023**, *253*, 126740.
177. Jiang, R.; Li, H.; Yang, J.; Shen, X.; Song, C.; Yang, Z.; Wang, X.; Huang, Y.; Lan, X.; Lei, C., et al. circRNA Profiling Reveals an Abundant circFUT10 that Promotes Adipocyte Proliferation and Inhibits Adipocyte Differentiation via Sponging let-7. *Mol Ther Nucleic Acids* **2020**, *20*, 491-501.

178. Zhu, Y.; Gui, W.; Lin, X.; Li, H. Knock-down of circular RNA H19 induces human adipose-derived stem cells adipogenic differentiation via a mechanism involving the polypyrimidine tract-binding protein 1. *Exp Cell Res* **2020**, *387*, 111753.
179. Li, M.; Li, J.; Ji, M.; An, J.; Zhao, T.; Yang, Y.; Cai, C.; Gao, P.; Cao, G.; Guo, X., et al. CircHOMER1 inhibits porcine adipogenesis via the miR-23b/SIRT1 axis. *FASEB J* **2023**, *37*, e22828.
180. Shen, X.; Tang, J.; Ru, W.; Zhang, X.; Huang, Y.; Lei, C.; Cao, H.; Lan, X.; Chen, H. CircINSR Regulates Fetal Bovine Muscle and Fat Development. *Front Cell Dev Biol* **2020**, *8*, 615638.
181. Zhao, B.; Zhang, H.; Zhao, D.; Liang, Y.; Qiao, L.; Liu, J.; Pan, Y.; Yang, K.; Liu, W. circINSR Inhibits Adipogenic Differentiation of Adipose-Derived Stromal Vascular Fractions through the miR-152/MEOX2 Axis in Sheep. *Int J Mol Sci* **2023**, *24*.
182. Yue, X.; Fan, M.; Liang, Y.; Qiao, L.; Liu, J.; Pan, Y.; Yang, K.; Liu, W. circITGB1 Regulates Adipocyte Proliferation and Differentiation via the miR-23a/ARRB1 Pathway. *International Journal of Molecular Sciences* **2023**, *24*, 1976.
183. Li, H.; Zhang, H.; Dai, Y.; Li, S.; Gu, J.; Wu, R.; Jia, J.; Shen, J.; Zhang, Y.; Li, H., et al. CircITGB5 regulates the proliferation and adipogenic differentiation of chicken intramuscular preadipocytes through the miR-181b-5p/CPT1A axis. *Int J Biol Macromol* **2024**, *283*, 137608.
184. Rong, X.; Li, R.; Gong, T.; Li, H.; Zhao, X.; Cao, G.; Li, M.; Li, B.; Yang, Y.; Guo, X. CircMEF2C(2, 3) modulates proliferation and adipogenesis of porcine intramuscular preadipocytes by miR-383/671-3p/MEF2C axis. *iScience* **2024**, *27*, 109710.
185. Liu, X.; Bai, Y.; Cui, R.; He, S.; Zhao, X.; Wu, K.; Fang, M. Sus_circPAPPA2 Regulates Fat Deposition in Castrated Pigs through the miR-2366/GK Pathway. *Biomolecules* **2022**, *12*.
186. Singaravelu, R.; Quan, C.; Powdrill, M.H.; Shaw, T.A.; Srinivasan, P.; Lyn, R.K.; Alonzi, R.C.; Jones, D.M.; Filip, R.; Russell, R.S., et al. MicroRNA-7 mediates cross-talk between metabolic signaling pathways in the liver. *Sci Rep* **2018**, *8*, 361.
187. Frutos, M.F.; Pardo-Marques, V.; Torrecilla-Parra, M.; Rada, P.; Perez-Garcia, A.; Martin-Martin, Y.; de la Pena, G.; Gomez, A.; Toledano-Zaragoza, A.; Gomez-Coronado, D., et al. "MiR-7 controls cholesterol biosynthesis through posttranscriptional regulation of DHCR24 expression". *Biochim Biophys Acta Gene Regul Mech* **2023**, *1866*, 194938.
188. Nikpay, M.; Beehler, K.; Valsesia, A.; Hager, J.; Harper, M.E.; Dent, R.; McPherson, R. Genome-wide identification of circulating-miRNA expression quantitative trait loci reveals the role of several miRNAs in the regulation of cardiometabolic phenotypes. *Cardiovasc Res* **2019**, *115*, 1629-1645.
189. Tsiloulis, T.; Pike, J.; Powell, D.; Rossello, F.J.; Canny, B.J.; Meex, R.C.; Watt, M.J. Impact of endurance exercise training on adipocyte microRNA expression in overweight men. *FASEB J* **2017**, *31*, 161-171.
190. Zheng, L.; Lv, G.C.; Sheng, J.; Yang, Y.D. Effect of miRNA-10b in regulating cellular steatosis level by targeting PPAR-alpha expression, a novel mechanism for the pathogenesis of NAFLD. *J Gastroenterol Hepatol* **2010**, *25*, 156-163.

191. Ahn, J.; Lee, H.; Jung, C.H.; Ha, T. Lycopene inhibits hepatic steatosis via microRNA-21-induced downregulation of fatty acid-binding protein 7 in mice fed a high-fat diet. *Mol Nutr Food Res* **2012**, *56*, 1665-1674.
192. Kida, K.; Nakajima, M.; Mohri, T.; Oda, Y.; Takagi, S.; Fukami, T.; Yokoi, T. PPARalpha is regulated by miR-21 and miR-27b in human liver. *Pharm Res* **2011**, *28*, 2467-2476.
193. Feng, J.; Li, A.; Deng, J.; Yang, Y.; Dang, L.; Ye, Y.; Li, Y.; Zhang, W. miR-21 attenuates lipopolysaccharide-induced lipid accumulation and inflammatory response: potential role in cerebrovascular disease. *Lipids Health Dis* **2014**, *13*, 27.
194. Calo, N.; Ramadori, P.; Sobolewski, C.; Romero, Y.; Maeder, C.; Fournier, M.; Rantakari, P.; Zhang, F.P.; Poutanen, M.; Dufour, J.F., et al. Stress-activated miR-21/miR-21* in hepatocytes promotes lipid and glucose metabolic disorders associated with high-fat diet consumption. *Gut* **2016**, *65*, 1871-1881.
195. Wang, Y.; Yang, L.Z.; Yang, D.G.; Zhang, Q.Y.; Deng, Z.N.; Wang, K.; Mao, X.J. MiR-21 antagomir improves insulin resistance and lipid metabolism disorder in streptozotocin-induced type 2 diabetes mellitus rats. *Ann Palliat Med* **2020**, *9*, 394-404.
196. Wu, Y.; Zhao, J.; Cui, C.; Zhang, Y.; Zhu, Q.; Han, S.; Yang, C.; Yin, H. MiRNA-21-5p induces chicken hepatic lipogenesis by targeting NFIB and KLF3 to suppress the PI3K/AKT signaling pathway. *J Anim Sci* **2024**, *102*.
197. Li, Y.; Fang, J.; Jiao, Y.; Lv, Q.; Xiao, X.; Zheng, S.; Chen, X.; Song, J.; Zhang, X.; Zhang, L., et al. MiR-21-5p promotes differentiation and reduces oleic acid-induced lipid droplet accumulation in C2C12 myoblasts by targeting FBXO11. *Anim Biosci* **2025**, 10.5713/ab.24.0665.
198. Soh, J.; Iqbal, J.; Queiroz, J.; Fernandez-Hernando, C.; Hussain, M.M. MicroRNA-30c reduces hyperlipidemia and atherosclerosis in mice by decreasing lipid synthesis and lipoprotein secretion. *Nat Med* **2013**, *19*, 892-900.
199. Irani, S.; Pan, X.; Peck, B.C.; Iqbal, J.; Sethupathy, P.; Hussain, M.M. MicroRNA-30c Mimic Mitigates Hypercholesterolemia and Atherosclerosis in Mice. *J Biol Chem* **2016**, *291*, 18397-18409.
200. Fan, J.; Li, H.; Nie, X.; Yin, Z.; Zhao, Y.; Chen, C.; Wen Wang, D. MiR-30c-5p ameliorates hepatic steatosis in leptin receptor-deficient (db/db) mice via down-regulating FASN. *Oncotarget* **2017**, *8*, 13450-13463.
201. Sodi, R.; Eastwood, J.; Caslake, M.; Packard, C.J.; Denby, L. Relationship between circulating microRNA-30c with total- and LDL-cholesterol, their circulatory transportation and effect of statins. *Clin Chim Acta* **2017**, *466*, 13-19.
202. Irani, S.; Iqbal, J.; Antoni, W.J.; Ijaz, L.; Hussain, M.M. microRNA-30c reduces plasma cholesterol in homozygous familial hypercholesterolemic and type 2 diabetic mouse models. *J Lipid Res* **2018**, *59*, 144-154.
203. Yaman, S.O.; Orem, A.; Yucesan, F.B.; Kural, B.V.; Orem, C. Evaluation of circulating miR-122, miR-30c and miR-33a levels and their association with lipids, lipoproteins in postprandial lipemia. *Life Sci* **2021**, *264*, 118585.
204. Rayner, K.J.; Suarez, Y.; Davalos, A.; Parathath, S.; Fitzgerald, M.L.; Tamehiro, N.; Fisher, E.A.; Moore, K.J.; Fernandez-Hernando, C. MiR-33 contributes to the regulation of cholesterol homeostasis. *Science* **2010**, *328*, 1570-1573.

205. Najafi-Shoushtari, S.H.; Kristo, F.; Li, Y.; Shioda, T.; Cohen, D.E.; Gerszten, R.E.; Naar, A.M. MicroRNA-33 and the SREBP host genes cooperate to control cholesterol homeostasis. *Science* **2010**, *328*, 1566-1569.
206. Horie, T.; Ono, K.; Horiguchi, M.; Nishi, H.; Nakamura, T.; Nagao, K.; Kinoshita, M.; Kuwabara, Y.; Marusawa, H.; Iwanaga, Y., et al. MicroRNA-33 encoded by an intron of sterol regulatory element-binding protein 2 (Srebp2) regulates HDL in vivo. *Proc Natl Acad Sci U S A* **2010**, *107*, 17321-17326.
207. Rotllan, N.; Ramirez, C.M.; Aryal, B.; Esau, C.C.; Fernandez-Hernando, C. Therapeutic silencing of microRNA-33 inhibits the progression of atherosclerosis in Ldlr-/- mice--brief report. *Arterioscler Thromb Vasc Biol* **2013**, *33*, 1973-1977.
208. Rayner, K.J.; Sheedy, F.J.; Esau, C.C.; Hussain, F.N.; Temel, R.E.; Parathath, S.; van Gils, J.M.; Rayner, A.J.; Chang, A.N.; Suarez, Y., et al. Antagonism of miR-33 in mice promotes reverse cholesterol transport and regression of atherosclerosis. *J Clin Invest* **2011**, *121*, 2921-2931.
209. Rottiers, V.; Obad, S.; Petri, A.; McGarrah, R.; Lindholm, M.W.; Black, J.C.; Sinha, S.; Goody, R.J.; Lawrence, M.S.; deLemos, A.S., et al. Pharmacological inhibition of a microRNA family in nonhuman primates by a seed-targeting 8-mer antimiR. *Sci Transl Med* **2013**, *5*, 212ra162.
210. Rayner, K.J.; Esau, C.C.; Hussain, F.N.; McDaniel, A.L.; Marshall, S.M.; van Gils, J.M.; Ray, T.D.; Sheedy, F.J.; Goedeke, L.; Liu, X., et al. Inhibition of miR-33a/b in non-human primates raises plasma HDL and lowers VLDL triglycerides. *Nature* **2011**, *478*, 404-407.
211. Li, T.; Francl, J.M.; Boehme, S.; Chiang, J.Y. Regulation of cholesterol and bile acid homeostasis by the cholesterol 7alpha-hydroxylase/steroid response element-binding protein 2/microRNA-33a axis in mice. *Hepatology* **2013**, *58*, 1111-1121.
212. Allen, R.M.; Marquart, T.J.; Albert, C.J.; Suchy, F.J.; Wang, D.Q.; Ananthanarayanan, M.; Ford, D.A.; Baldan, A. miR-33 controls the expression of biliary transporters, and mediates statin- and diet-induced hepatotoxicity. *EMBO Mol Med* **2012**, *4*, 882-895.
213. Xie, Q.; Peng, J.; Guo, Y.; Li, F. MicroRNA-33-5p inhibits cholesterol efflux in vascular endothelial cells by regulating citrate synthase and ATP-binding cassette transporter A1. *BMC Cardiovasc Disord* **2021**, *21*, 433.
214. Davalos, A.; Goedeke, L.; Smibert, P.; Ramirez, C.M.; Warrier, N.P.; Andreo, U.; Cirera-Salinas, D.; Rayner, K.; Suresh, U.; Pastor-Pareja, J.C., et al. miR-33a/b contribute to the regulation of fatty acid metabolism and insulin signaling. *Proc Natl Acad Sci U S A* **2011**, *108*, 9232-9237.
215. Shao, F.; Wang, X.; Yu, J.; Shen, K.; Qi, C.; Gu, Z. Expression of miR-33 from an SREBP2 intron inhibits the expression of the fatty acid oxidation-regulatory genes CROT and HADHB in chicken liver. *Br Poult Sci* **2019**, *60*, 115-124.
216. Choi, S.E.; Fu, T.; Seok, S.; Kim, D.H.; Yu, E.; Lee, K.W.; Kang, Y.; Li, X.; Kemper, B.; Kemper, J.K. Elevated microRNA-34a in obesity reduces NAD+ levels and SIRT1 activity by directly targeting NAMPT. *Aging Cell* **2013**, *12*, 1062-1072.
217. Oda, Y.; Nakajima, M.; Tsuneyama, K.; Takamiya, M.; Aoki, Y.; Fukami, T.; Yokoi, T. Retinoid X receptor alpha in human liver is regulated by miR-34a. *Biochem Pharmacol* **2014**, *90*, 179-187.

218. Fu, T.; Seok, S.; Choi, S.; Huang, Z.; Suino-Powell, K.; Xu, H.E.; Kemper, B.; Kemper, J.K. MicroRNA 34a inhibits beige and brown fat formation in obesity in part by suppressing adipocyte fibroblast growth factor 21 signaling and SIRT1 function. *Mol Cell Biol* **2014**, *34*, 4130-4142.
219. Xu, Y.; Zalzala, M.; Xu, J.; Li, Y.; Yin, L.; Zhang, Y. A metabolic stress-inducible miR-34a-HNF4alpha pathway regulates lipid and lipoprotein metabolism. *Nat Commun* **2015**, *6*, 7466.
220. Xu, Y.; Xu, Y.; Zhu, Y.; Sun, H.; Juguilon, C.; Li, F.; Fan, D.; Yin, L.; Zhang, Y. Macrophage miR-34a Is a Key Regulator of Cholesterol Efflux and Atherosclerosis. *Mol Ther* **2020**, *28*, 202-216.
221. Wang, L.; Sun, M.; Cao, Y.; Ma, L.; Shen, Y.; Velikanova, A.A.; Li, X.; Sun, C.; Zhao, Y. miR-34a regulates lipid metabolism by targeting SIRT1 in non-alcoholic fatty liver disease with iron overload. *Arch Biochem Biophys* **2020**, *695*, 108642.
222. Wang, W.; Li, X.; Ding, N.; Teng, J.; Zhang, S.; Zhang, Q.; Tang, H. miR-34a regulates adipogenesis in porcine intramuscular adipocytes by targeting ACSL4. *BMC Genet* **2020**, *21*, 33.
223. Xu, Y.; Zhu, Y.; Hu, S.; Pan, X.; Bawa, F.C.; Wang, H.H.; Wang, D.Q.; Yin, L.; Zhang, Y. Hepatocyte miR-34a is a key regulator in the development and progression of non-alcoholic fatty liver disease. *Mol Metab* **2021**, *51*, 101244.
224. Wang, L.; Xie, Y.; Chen, W.; Zhang, Y.; Zeng, Y. miR-34a Regulates Lipid Droplet Deposition in 3T3-L1 and C2C12 Cells by Targeting LEF1. *Cells* **2022**, *12*.
225. Kim, J.; Yoon, H.; Ramirez, C.M.; Lee, S.M.; Hoe, H.S.; Fernandez-Hernando, C.; Kim, J. MiR-106b impairs cholesterol efflux and increases Abeta levels by repressing ABCA1 expression. *Exp Neurol* **2012**, *235*, 476-483.
226. Esau, C.; Davis, S.; Murray, S.F.; Yu, X.X.; Pandey, S.K.; Pear, M.; Watts, L.; Booten, S.L.; Graham, M.; McKay, R., et al. miR-122 regulation of lipid metabolism revealed by in vivo antisense targeting. *Cell Metab* **2006**, *3*, 87-98.
227. Song, K.H.; Li, T.; Owsley, E.; Chiang, J.Y. A putative role of micro RNA in regulation of cholesterol 7alpha-hydroxylase expression in human hepatocytes. *J Lipid Res* **2010**, *51*, 2223-2233.
228. Iliopoulos, D.; Drosatos, K.; Hiyama, Y.; Goldberg, I.J.; Zannis, V.I. MicroRNA-370 controls the expression of microRNA-122 and Cpt1alpha and affects lipid metabolism. *J Lipid Res* **2010**, *51*, 1513-1523.
229. Wu, G.Y.; Rui, C.; Chen, J.Q.; Sho, E.; Zhan, S.S.; Yuan, X.W.; Ding, Y.T. MicroRNA-122 Inhibits Lipid Droplet Formation and Hepatic Triglyceride Accumulation via Yin Yang 1. *Cell Physiol Biochem* **2017**, *44*, 1651-1664.
230. Zhang, J.; Wang, Q.; Zhao, X.; Wang, L.; Wang, X.; Wang, J.; Dong, B.; Gong, D. MicroRNA-122 targets genes related to goose fatty liver. *Poult Sci* **2018**, *97*, 643-649.
231. Long, J.K.; Dai, W.; Zheng, Y.W.; Zhao, S.P. miR-122 promotes hepatic lipogenesis via inhibiting the LKB1/AMPK pathway by targeting Sirt1 in non-alcoholic fatty liver disease. *Mol Med* **2019**, *25*, 26.
232. Hu, Y.; Du, G.; Li, G.; Peng, X.; Zhang, Z.; Zhai, Y. The miR-122 inhibition alleviates lipid accumulation and inflammation in NAFLD cell model. *Arch Physiol Biochem* **2021**, *127*, 385-389.

233. Wagschal, A.; Najafi-Shoushtari, S.H.; Wang, L.; Goedeke, L.; Sinha, S.; deLemos, A.S.; Black, J.C.; Ramirez, C.M.; Li, Y.; Tewhey, R., et al. Genome-wide identification of microRNAs regulating cholesterol and triglyceride homeostasis. *Nat Med* **2015**, *21*, 1290-1297.
234. Goedeke, L.; Rotllan, N.; Canfran-Duque, A.; Aranda, J.F.; Ramirez, C.M.; Araldi, E.; Lin, C.S.; Anderson, N.N.; Wagschal, A.; de Cabo, R., et al. MicroRNA-148a regulates LDL receptor and ABCA1 expression to control circulating lipoprotein levels. *Nat Med* **2015**, *21*, 1280-1289.
235. Cheng, L.; Zhu, Y.; Han, H.; Zhang, Q.; Cui, K.; Shen, H.; Zhang, J.; Yan, J.; Prochownik, E.; Li, Y. MicroRNA-148a deficiency promotes hepatic lipid metabolism and hepatocarcinogenesis in mice. *Cell Death Dis* **2017**, *8*, e2916.
236. Wang, N.; He, L.; Lin, H.; Tan, L.; Sun, Y.; Zhang, X.; Danser, A.H.J.; Lu, H.S.; He, Y.; Lu, X. MicroRNA-148a regulates low-density lipoprotein metabolism by repressing the (pro)renin receptor. *PLoS One* **2020**, *15*, e0225356.
237. Wang, L.; Jia, X.J.; Jiang, H.J.; Du, Y.; Yang, F.; Si, S.Y.; Hong, B. MicroRNAs 185, 96, and 223 repress selective high-density lipoprotein cholesterol uptake through posttranscriptional inhibition. *Mol Cell Biol* **2013**, *33*, 1956-1964.
238. Vickers, K.C.; Landstreet, S.R.; Levin, M.G.; Shoucri, B.M.; Toth, C.L.; Taylor, R.C.; Palmisano, B.T.; Tabet, F.; Cui, H.L.; Rye, K.A., et al. MicroRNA-223 coordinates cholesterol homeostasis. *Proc Natl Acad Sci U S A* **2014**, *111*, 14518-14523.
239. Niu, Q.; Wang, T.; Wang, Z.; Wang, F.; Huang, D.; Sun, H.; Liu, H. Adipose-derived mesenchymal stem cell-secreted extracellular vesicles alleviate non-alcoholic fatty liver disease via delivering miR-223-3p. *Adipocyte* **2022**, *11*, 572-587.
240. Shen, B.; Pan, Q.; Yang, Y.; Gao, Y.; Liu, X.; Li, W.; Han, Y.; Yuan, X.; Qu, Y.; Zhao, Z. miR-224 Affects Mammary Epithelial Cell Apoptosis and Triglyceride Production by Downregulating ACADM and ALDH2 Genes. *DNA Cell Biol* **2017**, *36*, 26-33.
241. Wang, C.; Zhao, J.; Feng, X.; Zhao, W.; Ma, R.; Yu, B.; Xue, L.; Wang, H.; Chen, Y.; Zhang, J., et al. bta-miR-224 regulates milk fat metabolism by targeting FABP4 in bovine mammary epithelial cells. *Genomics* **2024**, *116*, 110955.
242. Meiler, S.; Baumer, Y.; Toulmin, E.; Seng, K.; Boisvert, W.A. MicroRNA 302a is a novel modulator of cholesterol homeostasis and atherosclerosis. *Arterioscler Thromb Vasc Biol* **2015**, *35*, 323-331.
243. Hoekstra, M.; van der Sluis, R.J.; Kuiper, J.; Van Berkel, T.J. Nonalcoholic fatty liver disease is associated with an altered hepatocyte microRNA profile in LDL receptor knockout mice. *J Nutr Biochem* **2012**, *23*, 622-628.
244. Gerin, I.; Bommer, G.T.; McCoin, C.S.; Sousa, K.M.; Krishnan, V.; MacDougald, O.A. Roles for miRNA-378/378* in adipocyte gene expression and lipogenesis. *Am J Physiol Endocrinol Metab* **2010**, *299*, E198-206.
245. Carrer, M.; Liu, N.; Grueter, C.E.; Williams, A.H.; Frisard, M.I.; Hulver, M.W.; Bassel-Duby, R.; Olson, E.N. Control of mitochondrial metabolism and systemic energy homeostasis by microRNAs 378 and 378*. *Proc Natl Acad Sci U S A* **2012**, *109*, 15330-15335.

246. Liu, W.; Cao, H.; Ye, C.; Chang, C.; Lu, M.; Jing, Y.; Zhang, D.; Yao, X.; Duan, Z.; Xia, H., et al. Hepatic miR-378 targets p110alpha and controls glucose and lipid homeostasis by modulating hepatic insulin signalling. *Nat Commun* **2014**, *5*, 5684.
247. Zhang, Y.; Li, C.; Li, H.; Song, Y.; Zhao, Y.; Zhai, L.; Wang, H.; Zhong, R.; Tang, H.; Zhu, D. miR-378 Activates the Pyruvate-PEP Futile Cycle and Enhances Lipolysis to Ameliorate Obesity in Mice. *EBioMedicine* **2016**, *5*, 93-104.
248. Zhang, T.; Zhao, X.; Steer, C.J.; Yan, G.; Song, G. A negative feedback loop between microRNA-378 and Nrf1 promotes the development of hepatosteatosis in mice treated with a high fat diet. *Metabolism* **2018**, *85*, 183-191.
249. Sun, C.; Liu, W.; Lu, Z.; Li, Y.; Liu, S.; Tang, Z.; Yan, Y.; Li, Z.; Feng, H.; Zhang, D., et al. Hepatic miR-378 modulates serum cholesterol levels by regulating hepatic bile acid synthesis. *Theranostics* **2021**, *11*, 4363-4380.
250. Dong, J.; He, M.; Li, J.; Pessentheiner, A.; Wang, C.; Zhang, J.; Sun, Y.; Wang, W.T.; Zhang, Y.; Liu, J., et al. microRNA-483 ameliorates hypercholesterolemia by inhibiting PCSK9 production. *JCI Insight* **2020**, *5*.
251. Sandovici, I.; Fernandez-Twinn, D.S.; Campbell, N.; Cooper, W.N.; Sekita, Y.; Zvetkova, I.; Ferland-McCollough, D.; Prosser, H.M.; Oyama, L.M.; Pantaleao, L.C., et al. Overexpression of Igf2-derived Mir483 inhibits Igf1 expression and leads to developmental growth restriction and metabolic dysfunction in mice. *Cell Rep* **2024**, *43*, 114750.
252. Salerno, A.G.; van Solingen, C.; Scotti, E.; Wanschel, A.; Afonso, M.S.; Oldebeken, S.R.; Spiro, W.; Tontonoz, P.; Rayner, K.J.; Moore, K.J. LDL Receptor Pathway Regulation by miR-224 and miR-520d. *Front Cardiovasc Med* **2020**, *7*, 81.
253. Ou, Z.; Wada, T.; Gramignoli, R.; Li, S.; Strom, S.C.; Huang, M.; Xie, W. MicroRNA hsa-miR-613 targets the human LXRalpha gene and mediates a feedback loop of LXRalpha autoregulation. *Mol Endocrinol* **2011**, *25*, 584-596.
254. Zhong, D.; Zhang, Y.; Zeng, Y.J.; Gao, M.; Wu, G.Z.; Hu, C.J.; Huang, G.; He, F.T. MicroRNA-613 represses lipogenesis in HepG2 cells by downregulating LXRalpha. *Lipids Health Dis* **2013**, *12*, 32.
255. Zhao, R.; Feng, J.; He, G. miR-613 regulates cholesterol efflux by targeting LXRalpha and ABCA1 in PPARgamma activated THP-1 macrophages. *Biochem Biophys Res Commun* **2014**, *448*, 329-334.
256. Yao, Y.; Li, Q.; Gao, P.; Wang, W.; Chen, L.; Zhang, J.; Xu, Y. Glucagon-like peptide-1 contributes to increases ABCA1 expression by downregulating miR-758 to regulate cholesterol homeostasis. *Biochem Biophys Res Commun* **2018**, *497*, 652-658.
257. Li, B.R.; Xia, L.Q.; Liu, J.; Liao, L.L.; Zhang, Y.; Deng, M.; Zhong, H.J.; Feng, T.T.; He, P.P.; Ouyang, X.P. miR-758-5p regulates cholesterol uptake via targeting the CD36 3'UTR. *Biochem Biophys Res Commun* **2017**, *494*, 384-389.
258. Ramirez, C.M.; Davalos, A.; Goedeke, L.; Salerno, A.G.; Warrier, N.; Cirera-Salinas, D.; Suarez, Y.; Fernandez-Hernando, C. MicroRNA-758 regulates cholesterol efflux through posttranscriptional repression of ATP-binding cassette transporter A1. *Arterioscler Thromb Vasc Biol* **2011**, *31*, 2707-2714.

259. Halley, P.; Kadakkuzha, B.M.; Faghihi, M.A.; Magistri, M.; Zeier, Z.; Khorkova, O.; Coito, C.; Hsiao, J.; Lawrence, M.; Wahlestedt, C. Regulation of the apolipoprotein gene cluster by a long noncoding RNA. *Cell Rep* **2014**, *6*, 222-230.
260. Qin, W.; Li, X.; Xie, L.; Li, S.; Liu, J.; Jia, L.; Dong, X.; Ren, X.; Xiao, J.; Yang, C., et al. A long non-coding RNA, APOA4-AS, regulates APOA4 expression depending on HuR in mice. *Nucleic Acids Res* **2016**, *44*, 6423-6433.
261. Liu, G.; Zheng, X.; Xu, Y.; Lu, J.; Chen, J.; Huang, X. Long non-coding RNAs expression profile in HepG2 cells reveals the potential role of long non-coding RNAs in the cholesterol metabolism. *Chin Med J (Engl)* **2015**, *128*, 91-97.
262. Zhao, X.Y.; Xiong, X.; Liu, T.; Mi, L.; Peng, X.; Rui, C.; Guo, L.; Li, S.; Li, X.; Lin, J.D. Long noncoding RNA licensing of obesity-linked hepatic lipogenesis and NAFLD pathogenesis. *Nat Commun* **2018**, *9*, 2986.
263. Li, H.; Han, S.; Sun, Q.; Yao, Y.; Li, S.; Yuan, C.; Zhang, B.; Jing, B.; Wu, J.; Song, Y., et al. Long non-coding RNA CDKN2B-AS1 reduces inflammatory response and promotes cholesterol efflux in atherosclerosis by inhibiting ADAM10 expression. *Aging (Albany NY)* **2019**, *11*, 1695-1715.
264. Hennessy, E.J.; van Solingen, C.; Scacalossi, K.R.; Ouimet, M.; Afonso, M.S.; Prins, J.; Koelwyn, G.J.; Sharma, M.; Ramkhelawon, B.; Carpenter, S., et al. The long noncoding RNA CHROME regulates cholesterol homeostasis in primate. *Nat Metab* **2019**, *1*, 98-110.
265. Hu, Y.W.; Yang, J.Y.; Ma, X.; Chen, Z.P.; Hu, Y.R.; Zhao, J.Y.; Li, S.F.; Qiu, Y.R.; Lu, J.B.; Wang, Y.C., et al. A lincRNA-DYNLRB2-2/GPR119/GLP-1R/ABCA1-dependent signal transduction pathway is essential for the regulation of cholesterol homeostasis. *J Lipid Res* **2014**, *55*, 681-697.
266. Li, Y.; Shen, S.; Ding, S.; Wang, L. LincRNA DYN-LRB2-2 upregulates cholesterol efflux by decreasing TLR2 expression in macrophages. *J Cell Biochem* **2018**, *119*, 1911-1921.
267. Li, P.; Yan, X.; Xu, G.; Pang, Z.; Weng, J.; Yin, J.; Li, M.; Yu, L.; Chen, Q.; Sun, K. A novel plasma lncRNA ENST00000416361 is upregulated in coronary artery disease and is related to inflammation and lipid metabolism. *Mol Med Rep* **2020**, *21*, 2375-2384.
268. Cai, C.; Zhu, H.; Ning, X.; Li, L.; Yang, B.; Chen, S.; Wang, L.; Lu, X.; Gu, D. LncRNA ENST00000602558.1 regulates ABCG1 expression and cholesterol efflux from vascular smooth muscle cells through a p65-dependent pathway. *Atherosclerosis* **2019**, *285*, 31-39.
269. Meng, X.D.; Yao, H.H.; Wang, L.M.; Yu, M.; Shi, S.; Yuan, Z.X.; Liu, J. Knockdown of GAS5 Inhibits Atherosclerosis Progression via Reducing EZH2-Mediated ABCA1 Transcription in ApoE(-/-) Mice. *Mol Ther Nucleic Acids* **2020**, *19*, 84-96.
270. Yang, L.; Li, P.; Yang, W.; Ruan, X.; Kiesewetter, K.; Zhu, J.; Cao, H. Integrative Transcriptome Analyses of Metabolic Responses in Mice Define Pivotal LncRNA Metabolic Regulators. *Cell Metab* **2016**, *24*, 627-639.
271. Liu, C.; Yang, Z.; Wu, J.; Zhang, L.; Lee, S.; Shin, D.J.; Tran, M.; Wang, L. Long noncoding RNA H19 interacts with polypyrimidine tract-binding protein 1 to reprogram hepatic lipid homeostasis. *Hepatology* **2018**, *67*, 1768-1783.
272. Liu, J.; Tang, T.; Wang, G.D.; Liu, B. LncRNA-H19 promotes hepatic lipogenesis by directly regulating miR-130a/PPARgamma axis in non-alcoholic fatty liver disease. *Biosci Rep* **2019**, *39*.

273. Wang, H.; Cao, Y.; Shu, L.; Zhu, Y.; Peng, Q.; Ran, L.; Wu, J.; Luo, Y.; Zuo, G.; Luo, J., et al. Long non-coding RNA (lncRNA) H19 induces hepatic steatosis through activating MLXIPL and mTORC1 networks in hepatocytes. *J Cell Mol Med* **2020**, *24*, 1399-1412.
274. Huang, C.; Hu, Y.W.; Zhao, J.J.; Ma, X.; Zhang, Y.; Guo, F.X.; Kang, C.M.; Lu, J.B.; Xiu, J.C.; Sha, Y.H., et al. Long Noncoding RNA HOXC-AS1 Suppresses Ox-LDL-Induced Cholesterol Accumulation Through Promoting HOXC6 Expression in THP-1 Macrophages. *DNA Cell Biol* **2016**, *35*, 722-729.
275. Li, C.; Hu, Z.; Zhang, W.; Yu, J.; Yang, Y.; Xu, Z.; Luo, H.; Liu, X.; Liu, Y.; Chen, C., et al. Regulation of Cholesterol Homeostasis by a Novel Long Non-coding RNA LASER. *Sci Rep* **2019**, *9*, 7693.
276. Lluch, A.; Latorre, J.; Oliveras-Canellas, N.; Fernandez-Sanchez, A.; Moreno-Navarrete, J.M.; Castells-Nobau, A.; Comas, F.; Buxo, M.; Rodriguez-Hermosa, J.I.; Ballester, M., et al. A novel long non-coding RNA connects obesity to impaired adipocyte function. *Mol Metab* **2024**, *90*, 102040.
277. Huang, J.; Chen, S.; Cai, D.; Bian, D.; Wang, F. Long noncoding RNA lncARSR promotes hepatic cholesterol biosynthesis via modulating Akt/SREBP-2/HMGCR pathway. *Life Sci* **2018**, *203*, 48-53.
278. Molina, E.; Chew, G.S.; Myers, S.A.; Clarence, E.M.; Eales, J.M.; Tomaszewski, M.; Charchar, F.J. A Novel Y-Specific Long Non-Coding RNA Associated with Cellular Lipid Accumulation in HepG2 cells and Atherosclerosis-related Genes. *Sci Rep* **2017**, *7*, 16710.
279. Wang, J.; Yang, W.; Chen, Z.; Chen, J.; Meng, Y.; Feng, B.; Sun, L.; Dou, L.; Li, J.; Cui, Q., et al. Long Noncoding RNA lncSHGL Recruits hnRNPA1 to Suppress Hepatic Gluconeogenesis and Lipogenesis. *Diabetes* **2018**, *67*, 581-593.
280. Sallam, T.; Jones, M.C.; Gilliland, T.; Zhang, L.; Wu, X.; Eskin, A.; Sandhu, J.; Casero, D.; Vallim, T.Q.; Hong, C., et al. Feedback modulation of cholesterol metabolism by the lipid-responsive non-coding RNA LeXis. *Nature* **2016**, *534*, 124-128.
281. Han, L.; Huang, D.; Wu, S.; Liu, S.; Wang, C.; Sheng, Y.; Lu, X.; Broxmeyer, H.E.; Wan, J.; Yang, L. Lipid droplet-associated lncRNA LIPTER preserves cardiac lipid metabolism. *Nat Cell Biol* **2023**, *25*, 1033-1046.
282. Lan, X.; Yan, J.; Ren, J.; Zhong, B.; Li, J.; Li, Y.; Liu, L.; Yi, J.; Sun, Q.; Yang, X., et al. A novel long noncoding RNA Lnc-HC binds hnRNPA2B1 to regulate expressions of Cyp7a1 and Abca1 in hepatocytic cholesterol metabolism. *Hepatology* **2016**, *64*, 58-72.
283. Lan, X.; Wu, L.; Wu, N.; Chen, Q.; Li, Y.; Du, X.; Wei, C.; Feng, L.; Li, Y.; Osoro, E.K., et al. Long Noncoding RNA lnc-HC Regulates PPARgamma-Mediated Hepatic Lipid Metabolism through miR-130b-3p. *Mol Ther Nucleic Acids* **2019**, *18*, 954-965.
284. Guo, Y.; Tian, W.; Wang, D.; Yang, L.; Wang, Z.; Wu, X.; Zhi, Y.; Zhang, K.; Wang, Y.; Li, Z., et al. LncHLEF promotes hepatic lipid synthesis through miR-2188-3p/GATA6 axis and encoding peptides and enhances intramuscular fat deposition via exosome. *Int J Biol Macromol* **2023**, *253*, 127061.
285. Li, D.; Guo, L.; Deng, B.; Li, M.; Yang, T.; Yang, F.; Yang, Z. Long non-coding RNA HR1 participates in the expression of SREBP-1c through phosphorylation of the PDK1/AKT/FoxO1 pathway. *Mol Med Rep* **2018**, *18*, 2850-2856.

286. Li, P.; Ruan, X.; Yang, L.; Kiesewetter, K.; Zhao, Y.; Luo, H.; Chen, Y.; Gucek, M.; Zhu, J.; Cao, H. A liver-enriched long non-coding RNA, lncLSTR, regulates systemic lipid metabolism in mice. *Cell Metab* **2015**, *21*, 455-467.
287. Chu, K.; Zhao, N.; Hu, X.; Feng, R.; Zhang, L.; Wang, G.; Li, W.; Liu, L. LncNONMMUG027912 alleviates lipid accumulation through AMPKalpha/mTOR/SREBP1C axis in nonalcoholic fatty liver. *Biochem Biophys Res Commun* **2022**, *618*, 8-14.
288. Shen, X.; Zhang, Y.; Ji, X.; Li, B.; Wang, Y.; Huang, Y.; Zhang, X.; Yu, J.; Zou, R.; Qin, D., et al. Long Noncoding RNA lncRHL Regulates Hepatic VLDL Secretion by Modulating hnRNPU/BMAL1/MTTP Axis. *Diabetes* **2022**, *71*, 1915-1928.
289. Ma, X.; Wang, T.; Zhao, Z.L.; Jiang, Y.; Ye, S. Propofol Suppresses Proinflammatory Cytokine Production by Increasing ABCA1 Expression via Mediation by the Long Noncoding RNA LOC286367. *Mediators Inflamm* **2018**, *2018*, 8907143.
290. Yan, C.; Chen, J.; Chen, N. Long noncoding RNA MALAT1 promotes hepatic steatosis and insulin resistance by increasing nuclear SREBP-1c protein stability. *Sci Rep* **2016**, *6*, 22640.
291. Liu, L.; Tan, L.; Yao, J.; Yang, L. Long non-coding RNA MALAT1 regulates cholesterol accumulation in ox-LDL-induced macrophages via the microRNA-17-5p/ABCA1 axis. *Mol Med Rep* **2020**, *21*, 1761-1770.
292. Huang, P.; Huang, F.Z.; Liu, H.Z.; Zhang, T.Y.; Yang, M.S.; Sun, C.Z. LncRNA MEG3 functions as a ceRNA in regulating hepatic lipogenesis by competitively binding to miR-21 with LRP6. *Metabolism* **2019**, *94*, 1-8.
293. Zhang, L.; Yang, Z.; Trottier, J.; Barbier, O.; Wang, L. Long noncoding RNA MEG3 induces cholestatic liver injury by interaction with PTBP1 to facilitate shp mRNA decay. *Hepatology* **2017**, *65*, 604-615.
294. Sallam, T.; Jones, M.; Thomas, B.J.; Wu, X.; Gilliland, T.; Qian, K.; Eskin, A.; Casero, D.; Zhang, Z.; Sandhu, J., et al. Transcriptional regulation of macrophage cholesterol efflux and atherogenesis by a long noncoding RNA. *Nat Med* **2018**, *24*, 304-312.
295. Li, S.; Yang, S.; Qiu, C.; Sun, D. LncRNA MSC-AS1 facilitates lung adenocarcinoma through sponging miR-33b-5p to up-regulate GPAM. *Biochem Cell Biol* **2021**, *99*, 241-248.
296. Liu, X.; Liang, Y.; Song, R.; Yang, G.; Han, J.; Lan, Y.; Pan, S.; Zhu, M.; Liu, Y.; Wang, Y., et al. Long non-coding RNA NEAT1-modulated abnormal lipolysis via ATGL drives hepatocellular carcinoma proliferation. *Mol Cancer* **2018**, *17*, 90.
297. Wang, X. Down-regulation of lncRNA-NEAT1 alleviated the non-alcoholic fatty liver disease via mTOR/S6K1 signaling pathway. *J Cell Biochem* **2018**, *119*, 1567-1574.
298. Huang-Fu, N.; Cheng, J.S.; Wang, Y.; Li, Z.W.; Wang, S.H. Neat1 regulates oxidized low-density lipoprotein-induced inflammation and lipid uptake in macrophages via paraspeckle formation. *Mol Med Rep* **2018**, *17*, 3092-3098.
299. Sun, Y.; Song, Y.; Liu, C.; Geng, J. LncRNA NEAT1-MicroRNA-140 axis exacerbates nonalcoholic fatty liver through interrupting AMPK/SREBP-1 signaling. *Biochem Biophys Res Commun* **2019**, *516*, 584-590.

300. Chen, X.; Tan, X.R.; Li, S.J.; Zhang, X.X. LncRNA NEAT1 promotes hepatic lipid accumulation via regulating miR-146a-5p/ROCK1 in nonalcoholic fatty liver disease. *Life Sci* **2019**, *235*, 116829.
301. Wang, L.; Xia, J.W.; Ke, Z.P.; Zhang, B.H. Blockade of NEAT1 represses inflammation response and lipid uptake via modulating miR-342-3p in human macrophages THP-1 cells. *J Cell Physiol* **2019**, *234*, 5319-5326.
302. Fan, G.; Zhang, C.; Wei, X.; Wei, R.; Qi, Z.; Chen, K.; Cai, X.; Xu, L.; Tang, L.; Zhou, J., et al. NEAT1/hsa-miR-372–3p axis participates in rapamycin-induced lipid metabolic disorder. *Free Radical Biology and Medicine* **2021**, *167*, 1-11.
303. Hu, Y.W.; Zhao, J.Y.; Li, S.F.; Huang, J.L.; Qiu, Y.R.; Ma, X.; Wu, S.G.; Chen, Z.P.; Hu, Y.R.; Yang, J.Y., et al. RP5-833A20.1/miR-382-5p/NFIA-dependent signal transduction pathway contributes to the regulation of cholesterol homeostasis and inflammatory reaction. *Arterioscler Thromb Vasc Biol* **2015**, *35*, 87-101.
304. Mitchel, K.; Theusch, E.; Cubitt, C.; Dose, A.C.; Stevens, K.; Naidoo, D.; Medina, M.W. RP1-13D10.2 Is a Novel Modulator of Statin-Induced Changes in Cholesterol. *Circ Cardiovasc Genet* **2016**, *9*, 223-230.
305. Dong, X.H.; Lu, Z.F.; Kang, C.M.; Li, X.H.; Haworth, K.E.; Ma, X.; Lu, J.B.; Liu, X.H.; Fang, F.C.; Wang, C.S., et al. The Long Noncoding RNA RP11-728F11.4 Promotes Atherosclerosis. *Arterioscler Thromb Vasc Biol* **2021**, *41*, 1191-1204.
306. Mazar, J.; Zhao, W.; Khalil, A.M.; Lee, B.; Shelley, J.; Govindarajan, S.S.; Yamamoto, F.; Ratnam, M.; Aftab, M.N.; Collins, S., et al. The functional characterization of long noncoding RNA SPRY4-IT1 in human melanoma cells. *Oncotarget* **2014**, *5*, 8959-8969.
307. Chen, G.; Yu, D.; Nian, X.; Liu, J.; Koenig, R.J.; Xu, B.; Sheng, L. LncRNA SRA promotes hepatic steatosis through repressing the expression of adipose triglyceride lipase (ATGL). *Sci Rep* **2016**, *6*, 35531.
308. Yang, L.; Li, T. LncRNA TUG1 regulates ApoM to promote atherosclerosis progression through miR-92a/FXR1 axis. *J Cell Mol Med* **2020**, *24*, 8836-8848.
309. Zhang, L.; Cheng, H.; Yue, Y.; Li, S.; Zhang, D.; He, R. TUG1 knockdown ameliorates atherosclerosis via up-regulating the expression of miR-133a target gene FGF1. *Cardiovasc Pathol* **2018**, *33*, 6-15.
310. Guo, J.; Fang, W.; Sun, L.; Lu, Y.; Dou, L.; Huang, X.; Tang, W.; Yu, L.; Li, J. Ultraconserved element uc.372 drives hepatic lipid accumulation by suppressing miR-195/miR4668 maturation. *Nat Commun* **2018**, *9*, 612.
311. Li, Q.; Wang, Y.; Wu, S.; Zhou, Z.; Ding, X.; Shi, R.; Thorne, R.F.; Zhang, X.D.; Hu, W.; Wu, M. CircACC1 Regulates Assembly and Activation of AMPK Complex under Metabolic Stress. *Cell Metab* **2019**, *30*, 157-173 e157.
312. Cai, H.; Jiang, Z.; Yang, X.; Lin, J.; Cai, Q.; Li, X. Circular RNA HIPK3 contributes to hyperglycemia and insulin homeostasis by sponging miR-192-5p and upregulating transcription factor forkhead box O1. *Endocr J* **2020**, *67*, 397-408.
313. Chen, Q.; Liu, M.; Luo, Y.; Yu, H.; Zhang, J.; Li, D.; He, Q. Maternal obesity alters circRNA expression and the potential role of mmu_circRNA_0000660 via sponging miR_693 in offspring liver at weaning age. *Gene* **2020**, *731*, 144354.

314. Zhang, C.; Chen, K.; Wei, R.; Fan, G.; Cai, X.; Xu, L.; Cen, B.; Wang, J.; Xie, H.; Zheng, S., et al. The circFASN/miR-33a pathway participates in tacrolimus-induced dysregulation of hepatic triglyceride homeostasis. *Signal Transduct Target Ther* **2020**, *5*, 23.
315. Guo, X.Y.; Chen, J.N.; Sun, F.; Wang, Y.Q.; Pan, Q.; Fan, J.G. circRNA_0046367 Prevents Hepatoxicity of Lipid Peroxidation: An Inhibitory Role against Hepatic Steatosis. *Oxid Med Cell Longev* **2017**, *2017*, 3960197.
316. Guo, X.Y.; Sun, F.; Chen, J.N.; Wang, Y.Q.; Pan, Q.; Fan, J.G. circRNA_0046366 inhibits hepatocellular steatosis by normalization of PPAR signaling. *World J Gastroenterol* **2018**, *24*, 323-337.
317. Li, P.; Shan, K.; Liu, Y.; Zhang, Y.; Xu, L.; Xu, L. CircScd1 Promotes Fatty Liver Disease via the Janus Kinase 2/Signal Transducer and Activator of Transcription 5 Pathway. *Dig Dis Sci* **2019**, *64*, 113-122.
318. Refeat, M.M.; Hassan, N.A.; Ahmad, I.H.; Mostafa, E.R.M.; Amr, K.S. Correlation of circulating miRNA-33a and miRNA-122 with lipid metabolism among Egyptian patients with metabolic syndrome. *J Genet Eng Biotechnol* **2021**, *19*, 147.
319. Ying, W.; Riopel, M.; Bandyopadhyay, G.; Dong, Y.; Birmingham, A.; Seo, J.B.; Ofrecio, J.M.; Wollam, J.; Hernandez-Carretero, A.; Fu, W., et al. Adipose Tissue Macrophage-Derived Exosomal miRNAs Can Modulate In Vivo and In Vitro Insulin Sensitivity. *Cell* **2017**, *171*, 372-384 e312.
320. Pan, Y.; Hui, X.; Hoo, R.L.C.; Ye, D.; Chan, C.Y.C.; Feng, T.; Wang, Y.; Lam, K.S.L.; Xu, A. Adipocyte-secreted exosomal microRNA-34a inhibits M2 macrophage polarization to promote obesity-induced adipose inflammation. *J Clin Invest* **2019**, *129*, 834-849.
321. Xihua, L.; Shengjie, T.; Weiwei, G.; Matro, E.; Tingting, T.; Lin, L.; Fang, W.; Jiaqiang, Z.; Fenping, Z.; Hong, L. Circulating miR-143-3p inhibition protects against insulin resistance in Metabolic Syndrome via targeting of the insulin-like growth factor 2 receptor. *Transl Res* **2019**, *205*, 33-43.
322. Wang, X.; He, Y.; Mackowiak, B.; Gao, B. MicroRNAs as regulators, biomarkers and therapeutic targets in liver diseases. *Gut* **2021**, *70*, 784-795.
323. He, Y.; Rodrigues, R.M.; Wang, X.; Seo, W.; Ma, J.; Hwang, S.; Fu, Y.; Trojnar, E.; Matyas, C.; Zhao, S., et al. Neutrophil-to-hepatocyte communication via LDLR-dependent miR-223-enriched extracellular vesicle transfer ameliorates nonalcoholic steatohepatitis. *J Clin Invest* **2021**, *131*.
324. Schmidt, E.; Dhaouadi, I.; Gaziano, I.; Oliverio, M.; Klemm, P.; Awazawa, M.; Mitterer, G.; Fernandez-Rebollo, E.; Pradas-Juni, M.; Wagner, W., et al. LincRNA H19 protects from dietary obesity by constraining expression of monoallelic genes in brown fat. *Nat Commun* **2018**, *9*, 3622.
325. Sun, J.; Ruan, Y.; Wang, M.; Chen, R.; Yu, N.; Sun, L.; Liu, T.; Chen, H. Differentially expressed circulating LncRNAs and mRNA identified by microarray analysis in obese patients. *Sci Rep* **2016**, *6*, 35421.
326. Stapleton, K.; Das, S.; Reddy, M.A.; Leung, A.; Amaram, V.; Lanting, L.; Chen, Z.; Zhang, L.; Palanivel, R.; Deiuliis, J.A., et al. Novel Long Noncoding RNA, Macrophage Inflammation-Suppressing Transcript (MIST), Regulates Macrophage Activation During Obesity. *Arterioscler Thromb Vasc Biol* **2020**, *40*, 914-928.

327. Zhang, F.F.; Liu, Y.H.; Wang, D.W.; Liu, T.S.; Yang, Y.; Guo, J.M.; Pan, Y.; Zhang, Y.F.; Du, H.; Li, L., et al. Obesity-induced reduced expression of the lncRNA ROIT impairs insulin transcription by downregulation of Nkx6.1 methylation. *Diabetologia* **2020**, *63*, 811-824.
328. Song, Y.; Li, H.; Ren, X.; Li, H.; Feng, C. SNHG9, delivered by adipocyte-derived exosomes, alleviates inflammation and apoptosis of endothelial cells through suppressing TRADD expression. *Eur J Pharmacol* **2020**, *872*, 172977.
329. Lo, K.A.; Huang, S.; Walet, A.C.E.; Zhang, Z.C.; Leow, M.K.; Liu, M.; Sun, L. Adipocyte Long-Noncoding RNA Transcriptome Analysis of Obese Mice Identified Lnc-Leptin, Which Regulates Leptin. *Diabetes* **2018**, *67*, 1045-1056.
330. Zhang, X.; Xue, C.; Lin, J.; Ferguson, J.F.; Weiner, A.; Liu, W.; Han, Y.; Hinkle, C.; Li, W.; Jiang, H., et al. Interrogation of nonconserved human adipose lincRNAs identifies a regulatory role of linc-ADAL in adipocyte metabolism. *Sci Transl Med* **2018**, *10*.
331. Liu, Y.; Ji, Y.; Li, M.; Wang, M.; Yi, X.; Yin, C.; Wang, S.; Zhang, M.; Zhao, Z.; Xiao, Y. Integrated analysis of long noncoding RNA and mRNA expression profile in children with obesity by microarray analysis. *Sci Rep* **2018**, *8*, 8750.
332. Pan, J.X. LncRNA H19 promotes atherosclerosis by regulating MAPK and NF-kB signaling pathway. *Eur Rev Med Pharmacol Sci* **2017**, *21*, 322-328.
333. Li, F.P.; Lin, D.Q.; Gao, L.Y. LncRNA TUG1 promotes proliferation of vascular smooth muscle cell and atherosclerosis through regulating miRNA-21/PTEN axis. *Eur Rev Med Pharmacol Sci* **2018**, *22*, 7439-7447.
334. Ye, Z.M.; Yang, S.; Xia, Y.P.; Hu, R.T.; Chen, S.; Li, B.W.; Chen, S.L.; Luo, X.Y.; Mao, L.; Li, Y., et al. LncRNA MIAT sponges miR-149-5p to inhibit efferocytosis in advanced atherosclerosis through CD47 upregulation. *Cell Death Dis* **2019**, *10*, 138.
335. Tao, K.; Hu, Z.; Zhang, Y.; Jiang, D.; Cheng, H. LncRNA CASC11 improves atherosclerosis by downregulating IL-9 and regulating vascular smooth muscle cell apoptosis and proliferation. *Biosci Biotechnol Biochem* **2019**, *83*, 1284-1288.
336. Park, J.G.; Kim, G.; Jang, S.Y.; Lee, Y.R.; Lee, E.; Lee, H.W.; Han, M.H.; Chun, J.M.; Han, Y.S.; Yoon, J.S., et al. Plasma Long Noncoding RNA LeXis is a Potential Diagnostic Marker for Non-Alcoholic Steatohepatitis. *Life (Basel)* **2020**, *10*.
337. Sun, W.; Sun, X.; Chu, W.; Yu, S.; Dong, F.; Xu, G. CircRNA expression profiles in human visceral preadipocytes and adipocytes. *Mol Med Rep* **2020**, *21*, 815-821.
